# Subcellular location of source proteins improves prediction of neoantigens for immunotherapy

**DOI:** 10.15252/embj.2022111071

**Published:** 2022-10-31

**Authors:** Andrea Castro, Saghar Kaabinejadian, Hooman Yari, William Hildebrand, Maurizio Zanetti, Hannah Carter

**Affiliations:** ^1^ Bioinformatics and Systems Biology Program University of California San Diego La Jolla CA USA; ^2^ Department of Microbiology and Immunology University of Oklahoma Health Sciences Center Oklahoma City OK USA; ^3^ Pure MHC LLC Oklahoma City OK USA; ^4^ The Laboratory of Immunology and Department of Medicine University of California San Diego La Jolla CA USA; ^5^ Moores Cancer Center University of California San Diego La Jolla CA USA; ^6^ Department of Medicine, Division of Medical Genetics University of California San Diego La Jolla CA USA

**Keywords:** immunogenicity, Immunotherapy, major histocompatibility complex, neoantigen, subcellular location, Cancer, Computational Biology, Immunology

## Abstract

Antigen presentation via the major histocompatibility complex (MHC) is essential for anti‐tumor immunity. However, the rules that determine which tumor‐derived peptides will be immunogenic are still incompletely understood. Here, we investigated whether constraints on peptide accessibility to the MHC due to protein subcellular location are associated with peptide immunogenicity potential. Analyzing over 380,000 peptides from studies of MHC presentation and peptide immunogenicity, we find clear spatial biases in both eluted and immunogenic peptides. We find that including parent protein location improves the prediction of peptide immunogenicity in multiple datasets. In human immunotherapy cohorts, the location was associated with a neoantigen vaccination response, and immune checkpoint blockade responders generally had a higher burden of neopeptides from accessible locations. We conclude that protein subcellular location adds important information for optimizing cancer immunotherapies.

## Graphical abstract

Large‐scale analyses of peptide immunogenicity reveal that the inclusion of parent protein location improves prediction potential.

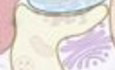

## Introduction

The presence of immunogenic antigens is necessary for neoantigen‐based cancer treatments such as neoantigen vaccines, immune checkpoint blockade, and adoptive T‐cell therapy to be effective. These immunotherapies all depend on cell surface display of tumor‐derived peptides by molecules of the major histocompatibility complex (MHC) mediating immune surveillance by T cells. As such, accurate characterization of the subset of immunogenic neoantigens should improve the design and application of immunotherapy and help identify potential responders.

A number of features have been identified as informative for prioritizing immunogenic neopeptides (i.e., neopeptides that are both displayed and recognized by T cells as foreign) including peptide–MHC stability, agretopicity (Ghorani *et al*, 2018), foreignness (Łuksza *et al*, 2017), hydrophobicity (Chowell *et al*, 2015; Zhou *et al*, 2019; Borden *et al*, 2022; Wells *et al*, 2020), mutation position within the neopeptide (Schmidt *et al*, 2021), and neopeptide RNA abundance (Wells *et al*, 2020; Borden *et al*, 2022). These features capture the potential for a peptide to be effectively presented by cell surface MHC and address characteristics of the neopeptide itself. While initial studies tie these features to immunogenicity, their utility for predicting immunogenicity varies across experiments and cohorts; for example, hydrophobicity was initially associated with increasing T‐cell epitope prediction (Chowell *et al*, 2015), but later found to be unimportant for peptide filtering (Wells *et al*, 2020). Ultimately, current tools still yield many false‐positive neoantigen predictions (Yadav *et al*, 2014; Castro *et al*, 2021), suggesting that the current set of features fails to capture other factors that contribute to T‐cell recognition of peptide‐bound MHC.

The canonical pathways by which peptides are added to the MHC for binding differ for class I (MHC‐I) and class II (MHC‐II), with class I peptides requiring transport to the endoplasmic reticulum via TAP transporters (Wieczorek *et al*, 2017) and class II peptides arriving via endosomes generated through phagocytosis or B‐cell receptor internalization by antigen‐presenting cells (Roche & Furuta, 2015). It stands to reason that these distinct pathways could result in peptides from different proteins being more accessible to MHC‐I versus MHC‐II molecules. Indeed, studies profiling eluted peptide–MHC complexes noted enrichment for peptide origin from intracellular compartments for MHC‐I‐eluted peptides, and for secreted, cell membrane, and extracellular proteins for MHC‐II‐eluted peptides (Bassani‐Sternberg *et al*, 2015; Schellens *et al*, 2015; Pearson *et al*, 2016; Abelin *et al*, 2019). Interestingly, a more recent work found a bias for MHC‐I‐presented peptides from proteins with certain molecular functions such as intracellular structural proteins, while MHC‐II was biased to present membrane transport proteins (preprint: Karnaukhov *et al*, 2021).

These observations imply that proteins in different cellular contexts such as location or molecular function, which are correlated (Lu & Hunter, 2004), can have varying levels of access to MHC‐I or MHC‐II presentation. This could further constrain the landscape of peptides that are presented to T cells during thymic selection and cancer development. In the first case, a tumor mutation that would otherwise be effectively bound and displayed by the MHC may not be presented because peptides from the source protein never reach the MHC. For the second case, effective presentation of self‐antigen during thymic development is required for clonal deletion of corresponding T cells. This pruning of the T‐cell repertoire is essential to prevent inappropriate activity against self; mutations to the AIRE gene that promotes tissue‐specific self‐antigen expression during thymic selection result in widespread, multi‐organ autoimmunity (Xing & Hogquist, 2012). However, if the peptide repertoire available for T‐cell selection is constrained by cellular context, it is conceivable that a portion of the T‐cell repertoire would still be capable of mounting a response against peptides from those hidden cellular contexts.

Based on this reasoning, we hypothesized that the subcellular location of a source protein could influence the immunogenic potential of its derivative peptides. We collected and analyzed data on peptides presented by the MHC and peptides documented to generate immune responses. To determine the extent to which cellular location can predict neopeptide immunogenicity, we trained and evaluated machine learning models on datasets of experimentally tested neopeptides. Finally, we evaluated the source protein subcellular location in the context of immunotherapy response and tumor remodeling by immunotherapy. Together, these analyses support the view that protein subcellular location is a determinant of neopeptide immunogenicity.

## Results

### Certain cellular components are enriched for immunogenic peptides

We performed gene ontology cellular component enrichment analysis on over 380,000 MHC‐I‐ and MHC‐II‐eluted peptides from a diverse set of normal tissues and tumor cell lines (Appendix Fig S1). We confirmed enrichment for MHC‐I‐presented peptides in the cytosol, nucleoplasm, and extracellular exosome components, and for MHC‐II‐presented peptides in the extracellular exosome, region, and space (Appendix Fig S1) as previously described (Bassani‐Sternberg *et al*, 2015; Schellens *et al*, 2015; Pearson *et al*, 2016; Abelin *et al*, 2019). Expression analysis of genes from enriched locations revealed increased expression compared to genes from depleted locations (Appendix Fig S2), although 22% of highly expressed genes were not significantly enriched in eluted peptide–MHC, reaffirming previous findings that gene expression alone does not drive peptide elution (Pearson *et al*, 2016). We also evaluated protein turnover rates from four human cell types including B cells, natural killer cells, hepatocytes, and monocytes (Mathieson *et al*, 2018) as peptides presented by MHC‐I are derived from degraded peptides in the cell (Milner *et al*, 2006). Thus, a high turnover rate could result in more peptides being available for presentation. Instead, we found that overall, proteins from enriched location categories tended to have longer predicted half‐lives (Appendix Fig S3), although the cell types evaluated were limited. To understand the implications of these findings for peptide‐directed T‐cell responses, we next sought to correlate peptide immunogenicity with subcellular origin.

We hypothesized that the effects of protein subcellular localization bias on peptide availability for presentation and T‐cell selection would influence the immunogenicity of tumor neoepitopes. To ultimately test whether the incorporation of peptide parent protein location as a feature could improve the prediction of neopeptide immunogenicity, we first evaluated the effect of protein subcellular location on the likelihood of observing peptides presented by the MHC using peptide elution data.

**Figure 1:**
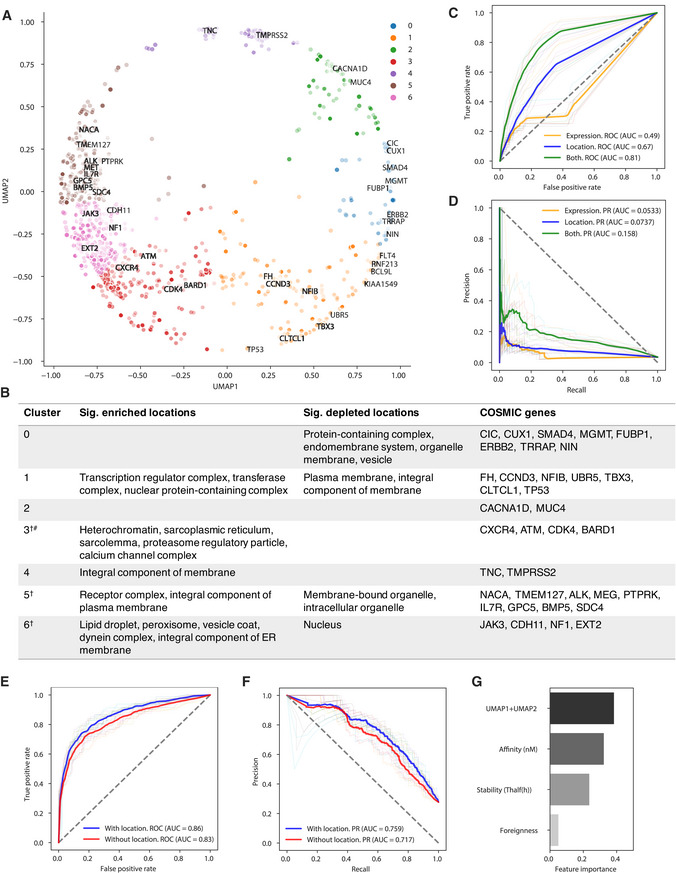
Overview of location features from eluted peptides and T‐cell assayed neoepitopes from IEDB AScatterplot of clustered UMAP location features (Materials and Methods) for source proteins of IEDB T‐cell assayed neoepitopes with COSMIC cancer genes highlighted.BTable annotating location clusters from the scatterplot with highlighted COSMIC cancer genes. ^†^PANTHER GO Slim CC used here for simplified terms. ^#^Many terms so only the top five are shown.C, D(C) Area under the receiver operating characteristic curve (AUROC) and (D) area under the precision–recall curve (AUPRC) for 10‐fold cross‐validation using a Random Forest model to predict protein elution in 721.221 B cells incorporating location, matched expression, or both (Materials and Methods). The faded lines indicate the respective area under the curve for each split.E, F(E) AUROC and (F) AUPRC for 10‐fold cross‐validation using a Random Forest model to predict immunogenicity in IEDB assayed neopeptides incorporating peptide affinity, stability, and foreignness (Materials and Methods) with and without parent protein location features.GBarplot of model feature importance.

Proteins have complex localization patterns; thus, we needed a strategy that could capture whether any location of a protein would contribute peptides that could be eluted from MHC molecules. To take advantage of gene ontology (GO) cellular component annotations, wherein proteins are annotated to multiple locations, we used a recently published set of pre‐trained, 200‐dimensional gene ontology embeddings (Kim *et al*, 2021). This approach represents each GO cellular component in the Poincare ball hyperbolic space. In this space, embedding vectors for multiple GO terms assigned to a single protein can be summed to obtain a single new vector that represents the multiple locations of that protein. We used UMAP to reduce the large 200‐dimensional vectors into two features (see Materials and Methods) allowing more convenient visualization (Fig 1A, Appendix Fig S4) as well as providing a more compact feature set for machine learning. Unsupervised clustering in UMAP space suggested 7 clusters, which showed enrichment for particular cellular components (Fig 1B), confirming that the non‐linear two‐dimensional mapping preserves information about complex protein subcellular location patterns.

To examine the relevance of these location features for predicting peptide elution, we evaluated eluted peptides from 721.221 B cells (*n* = 3,510). Gene expression has also been associated with cell surface presentation (Abelin *et al*, 2017 ). Since we had matched expression data for these eluted peptides (discussed in Appendix Figs S1–S4), we compared location to expression as predictors of whether a protein would be represented in the eluted peptides from cell surface MHC complexes. For a given protein, we labeled it as eluted (*n* = 638) or not (*n* = 17,307) based on the presence of 1 or more peptides mapping to that protein among the 3,510 eluted peptides (Materials and Methods). We trained a random forest model and found that location alone (67% AUC) has significantly improved AUROC compared to expression alone (49% AUC, DeLong's test *P*‐value = 1.34 e‐29). Location and expression features together achieve 81% AUROC and 15% AUPRC, respectively (Fig 1C and D). This supports an independent contribution of location overexpression.

Next, we sought to determine whether subcellular location effects on presentation also translated to effects on immunogenicity. We began by querying the immune epitope database (IEDB; Vita *et al*, 2019) for neoepitopes that were assayed for immunogenicity. After filtering (Materials and Methods), 2,943 neoepitopes remained, of which 813 (27.6%) were reported to elicit a positive T‐cell assay result. Cellular component enrichment analysis of parent proteins (*n* = 325) of immunogenic peptides did not reveal any significantly enriched locations, likely due to the limited sample size. However, these peptides did tend to come from locations where more eluted peptides were observed on average (Appendix Fig S5). Analysis of unique non‐immunogenic parent proteins (*n* = 772) whose peptides passed the minimum MHC‐I‐binding threshold (< 2 netMHCpan percentile rank) showed enrichment for locations including an integral component of the plasma membrane, which are depleted for not only eluted peptide–MHC (Appendix Fig S1) but also some other membrane locations that were enriched for MHC‐bound peptides, such as the endoplasmic and sarcoplasmic reticulum membranes.

We then used our 2D location embeddings to analyze the locations of unique source proteins across studies in the IEDB. We evaluated immunogenic and non‐immunogenic neopeptide source proteins and observed many overlapping locations between the two groups (Appendix Fig S6A and B). However, we still observed that certain locations generate more immunogenic peptides than others (Appendix Fig S6C) and *vice versa*, although these may reflect selection biases involved in choosing the proteins and peptides evaluated for immunogenicity in the various IEDB studies, and could change as available datasets grow.

### Parent protein location improves peptide immunogenicity prediction in multiple datasets

Next, we sought to test whether incorporating location as a feature would improve immunogenicity prediction. We performed 10‐fold cross‐validation using a random forest classifier on the IEDB dataset (Materials and Methods) with and without adding location to a feature set that comprised peptide–MHC‐binding affinity (nM; Jurtz *et al*, 2017), peptide–MHC stability (Rasmussen *et al*, 2016), and foreignness (Łuksza *et al*, 2017; Wells *et al*, 2020). This dataset did not include many MHC‐II peptides and did not provide enough information about MHC‐II alleles to calculate peptide–MHC affinities, therefore we focused on MHC‐I peptides. We found that adding location as a feature improved both the area under the receiver operating characteristic (ROC) curve (Fig 1E) and precision–recall (PR) curve (Fig 1F), and contributed to 38% of the model's predictive power (Fig 1G). As a control, we replaced location features with randomly sampled values ranging from −1 to 1 and found that the AUROC and AUPRC that incorporated these randomized values were greatly decreased (69% AUROC and 37% AUPRC). Next, we examined the predicted differences between the two models by using the median Youden index to classify peptides as immunogenic or not for each model. A total of 254 peptides were differentially classified between the two models, with 134 now classified as immunogenic and 120 not immunogenic in the location model. The reclassified peptides were enriched for true positives and negatives (Fisher's exact OR: 1.74, *P* = 0.04), and true positives included peptides from the cytosol while the true negatives included peptides from the nucleus (Appendix Fig S7). Interestingly, among these newly classified peptides, immunogenic versus non‐immunogenic peptides had significantly higher median GTEx gene expression but similar affinity, stability, and foreignness scores (Appendix Fig S7), suggesting that location may help predict gene expression. As gene expression was correlated with peptide–MHC elution (Appendix Fig S2; Abelin *et al*, 2017), we repeated the analysis; this time including median gene expression obtained from GTEx as a feature. We found that the benefit of including location as a feature persisted, suggesting that location provides information to predict immunogenicity that is distinct from expression (Appendix Fig S8).

**Figure 2:**
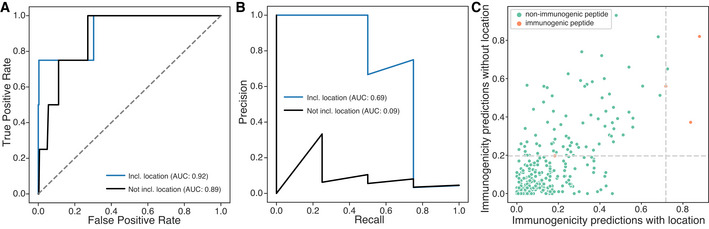
Predicting immunogenicity on the Wells validation dataset A, B(A) Area under the receiver operating characteristic curve (AUROC) (B) and area under the precision–recall curve (AUPRC) for the unseen validation dataset with and without parent protein location features.CScatterplot of the predicted probabilities for unseen test neopeptides to be immunogenic with and without location as a feature. Dashed lines indicate the Youden index for each model, used for optimal threshold predictions. False positives are reduced in the model with location.

We then tested our model on unseen datasets not included in the IEDB database. First, we analyzed around 900 peptides from Wells *et al* (2020) as this dataset represents the largest collection of immunologically tested peptides that we could identify. As these data were designed to benchmark neoantigen prediction algorithms, they were partitioned into a ~ 600 peptide discovery set and a ~ 300 peptide validation set. Initial analysis revealed differences in the distribution of MHC affinity and stability of immunogenic peptides between the IEDB and Wells datasets (Appendix Fig S9), which may be attributable to overall differences in MHC allele frequencies between the two datasets, and inherent differences in affinity and stability across MHC alleles themselves (Paul *et al*, 2013; Appendix Fig S10). We also found that the parent proteins of immunogenic peptides identified by Wells *et al*, originated from locations that were infrequently observed in the IEDB (Appendix Fig S10G), likely reflecting the use of different criteria for selecting proteins/peptides in the Wells study versus studies in IEDB. Interestingly, while a model trained solely on IEDB did not perform as well as a model trained on the discovery partition provided in the Wells study in ROC analysis (AUROC of 89% vs. 93%), it significantly improved the precision–recall curve (AUPRC of 64% vs. 9.2%; Appendix Fig S9). This suggests that filtering candidate neoantigens based on location may significantly reduce false‐positive predictions. To address systematic differences in the feature sets, we trained a new model combining the IEDB with the Wells discovery set. We were able to achieve a higher recall on the test set (AUROC of 92%) while retaining the benefit of reducing false positives (69% AUPRC), shown by fewer non‐immunogenic peptides (green points) falling above the Youden index threshold for the model with location (vertical dashed line) than in the model without location (horizontal dashed line; Fig 2A–C).

Several peptide features including tumor abundance (expression) and agretopicity that were identified in the Wells dataset as being predictive of immunogenicity were not available in the IEDB dataset. Therefore, we trained a separate model on the Wells discovery set alone incorporating these additional features, and tested it on the independent Wells test set. We found that incorporating location improved both the AUROC (89% vs. 67%) and AUPRC (9.6% vs. 7.5%). In this model, the location features contributed 22% of feature importance, just below affinity (Appendix Fig S11). These experiments suggest that location improves the prediction of immunogenic peptides, with the greatest benefit likely coming from a reduction in the number of false‐positive predictions. The large improvement in precision and recall when incorporating the IEDB underscores the benefit of a large training set for capturing the information provided by parent protein location.

We evaluated performance on a second independent dataset of 43 assayed MHC‐I neoepitopes from advanced ovarian cancer patients (Liu *et al*, 2019b) not seen in the IEDB cohort. Of these, only three (6.9%) were validated as immunogenic, further emphasizing the scarcity of true neoantigens. The tested neopeptides once again had a significantly different affinity and stability than the IEDB dataset, and while 16 locations were shared between datasets, these did not include the parent proteins for the three immunogenic peptides (Appendix Fig S12). We ran the model trained on IEDB alone with and without location features, as well as the model trained on both the IEDB and Wells datasets. We observed improved performance with the addition of datasets and incorporation of location (Appendix Fig S13), 45% vs. 65% AUROC and 6.2% vs. 9% AUPRC. Taken together, these findings suggest that immunogenicity prediction benefits from incorporating parent protein subcellular location and can be improved through the aggregation of independent datasets across cancer types.

### Immunotherapy response reflects neoepitope parent protein subcellular location

Most T‐cell‐based immunotherapies, such as neoantigen vaccines and immune checkpoint blockade (ICB), depend on the availability of immunogenic peptides to drive effector T‐cell responses. We speculated that if the parent protein subcellular location constrains the set of mutations in a tumor that could potentially be immunogenic, then we should find associations between location and immunotherapy responses. We evaluated three ways in which location might be apparent in human immunotherapy studies. First, we sought to determine whether the location was a determinant of T‐cell response in a neoantigen vaccine study, then we asked whether neopeptides from locations that were more immunogenic were more likely to be depleted by immunotherapy (immunoediting), and finally, investigated whether location could improve the estimation of the effective neoantigen burden and consequently, stratification of responders and non‐responders to ICB.

We first investigated the association of location with immune response in a neoantigen vaccine study (Sahin *et al*, 2017) and found that parent proteins of neopeptides able to induce a post‐vaccination response (75/125 tested; 120 distinct parent proteins) were enriched for locations previously observed to contain immunogenic peptides from the Wells, Liu (OV), and IEDB neoantigen datasets (Fisher's exact 3.49, *P* = 0.029). Because neoantigen vaccine studies have reported predominantly CD4^+^ T‐cell responses (Ott *et al*, 2017; Sahin *et al*, 2017; Hilf *et al*, 2019; Keskin *et al*, 2019), we also investigated whether vaccine neopeptides associated with an immune response came from locations from which more MHC‐I or MHC‐II peptides were eluted by the HLA ligand atlas. The majority of neopeptides tested (92/125, 73.6%) had parent proteins from which MHC‐I and MHC‐II peptides had been previously eluted. Nineteen neopeptides' parent proteins were only observed to be eluted from MHC‐I and 7 were exclusive to MHC‐II (Appendix Fig S14A). Unlike having parent proteins in a location previously associated with immunogenic peptides, the number of MHC‐eluted peptides from neopeptide parent proteins alone did not correlate with immune response, although there was a weak trend for neopeptide parent proteins exclusive to MHC‐II to have higher numbers of eluted peptides observed (Appendix Fig S14B). Thus, although we observed a correlation between the number of eluted peptides and immunogenicity in general (Appendix Fig S4), the subcellular location may be a more nuanced determinant of immunogenic potential.

In light of an association between parent protein subcellular location and post‐vaccine response, we speculated that tumor clones present pre‐treatment and eliminated during treatment would be more likely to harbor mutations in proteins from immunogenic locations. To further explore this possibility, we evaluated 73 melanoma patients with paired pre‐ and on‐treatment samples to see if there were notable differences between eliminated (present pre‐treatment and not on‐treatment) and persistent (present both pre‐ and on‐treatment) neopeptides (Riaz *et al*, 2017). We focused on responders (*n* = 38, partial/complete response, or > 6 months of stable disease) as these patients should have a relatively intact immune response compared to non‐responders. While responders had a better overall presentation of evaluated neopeptides, neopeptides eliminated on‐treatment did not have significantly better overall MHC allele‐specific presentation compared to neopeptides retained pre‐ and on‐treatment in both responders and non‐responders (Appendix Fig S15), suggesting that neopeptide elimination is not driven solely by affinity or stability in this dataset. However, this analysis is complicated by the non‐independence of mutations that coexist within the same subclones. To investigate further, we examined 12,915 retained neopeptides from responders that were predicted to be presented by MHC‐I (NetMHCpan rank < 2) and, therefore, should have been eliminated by the patient's immune system. We found that eliminated neopeptides tended to be enriched for locations where immunogenic peptides were previously observed (combining the immunogenic peptides from the IEDB, Wells *et al*, Liu *et al*; Fisher's exact OR: 1.08, *P* = 0.09).

**Figure 3:**
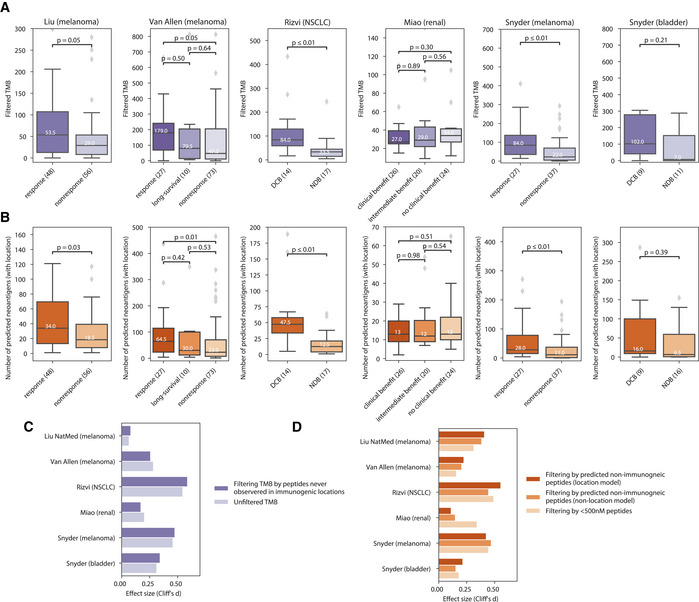
ICB responders carry a higher burden of mutations in proteins from immunogenic locations APredicted neoantigen burden versus response category in immunotherapy cohorts when retaining only mutations in proteins from subcellular locations previously observed to source immunogenic peptides.BPredicted neoantigen burden versus response category in immunotherapy cohorts where neoantigen status is predicted using a model trained on three sources of immunogenic peptide and features including peptide–MHC affinity, stability, and location.C, DBarplots of effect sizes between responders and non‐responders (C) where TMB is filtered to include only mutations from subcellular locations previously observed to source immunogenic peptides and (D) where neoantigen status is predicted using a model trained on three sources of immunogenic peptide and MHC affinity and stability, with and without location. Data information: Panels A and B include the median line, the boxes denote the interquartile range (IQR), whiskers denote the rest of the data distribution, and outliers are denoted by points determined by ± 1.5 * IQR. Sample size indicates number of patients. The Mann–Whitney *U* statistical test was performed.

Next, we studied the potential for parent protein location to improve ICB response stratification. We evaluated cohorts with whole‐exome sequencing data, as well as one profiled using a deep‐sequenced gene panel. We began by looking for the association of previously immunogenic locations with a response status. We found that in four of six evaluated immunotherapy cohorts, including melanoma, non‐small‐cell lung cancer (NSCLC), bladder, and renal cancer patients (Snyder *et al*, 2014, 2017; Rizvi *et al*, 2015; Van Allen *et al*, 2015; Miao *et al*, 2018; Liu *et al*, 2019a), considering only mutations from immunogenic locations in the TMB improved stratification of responders versus non‐responders, as defined by their respective original studies (Fig 3A). To incorporate other major determinants of immunogenicity such as affinity for the MHC, we used a model trained on all datasets with immunogenicity information (IEDB + Wells + Liu (ovarian)) to classify neopeptides in both ICB cohorts as immunogenic or not based on the Youden index of the trained model (Materials and Methods). We found that in four of the six evaluated immunotherapy cohorts, the predicted burden of immunogenic peptides based on location in responders was significantly higher than in non‐responders (Fig 3B). The change in effect size relative to baseline (TMB vs. location filtered TMB, and models with and without location vs. a basic < 500 nm peptide–MHC affinity filter) is shown in Fig 3C and D, respectively. Consistent with the approach of removing false positives, we saw a larger reduction in putative neoantigens in non‐responders than in responders in both analyses (Appendix Fig S16). In most cases, filtering out neopeptides predicted not to be immunogenic widened the gap between responders and non‐responders, supporting the potential of location to improve stratification of patient groups pre‐treatment.

Finally, we analyzed a cohort of 83 diverse tumors treated with immune checkpoint monotherapy that were profiled pre‐treatment with the Foundation Medicine gene panel (Goodman *et al*, 2020). In this cohort, we previously found that presence of at least one presentable driver mutation could further stratify responders and non‐responders in the context of covariates including sex, ethnicity, age, tumor type, TMB, and therapy type. Of the 325 genes on this panel, 40 (12.3%) encoded proteins with subcellular locations from which immunogenic peptides had previously been observed, including *ABL1*, *ALK*, *APC*, *ARAF*, *C11ORF30*, *EMSY*, *CCND3*, *CDK4*, *CDKN1A*, *CDKN2A*, *CREBBP*, *EGFR*, *EZH2*, *FAM46C*, *FGF19*, *FGF3*, *FGF4*, *FUBP1*, *GATA3*, *ID3*, *INPP4B*, *JAK1*, *KDM5C*, *KMT2D*, *MLL2*, *KRAS*, *MAP3K1*, *MDM4*, *MET*, *MYCL*, *MYCL1*, *NPM1*, *NT5C2*, *PALB2*, *PBRM1*, *PTPRO*, *RARA*, *SMO*, *TBX3*, *TET2*, *TIPARP*, and *TP53*.

**Figure 4:**
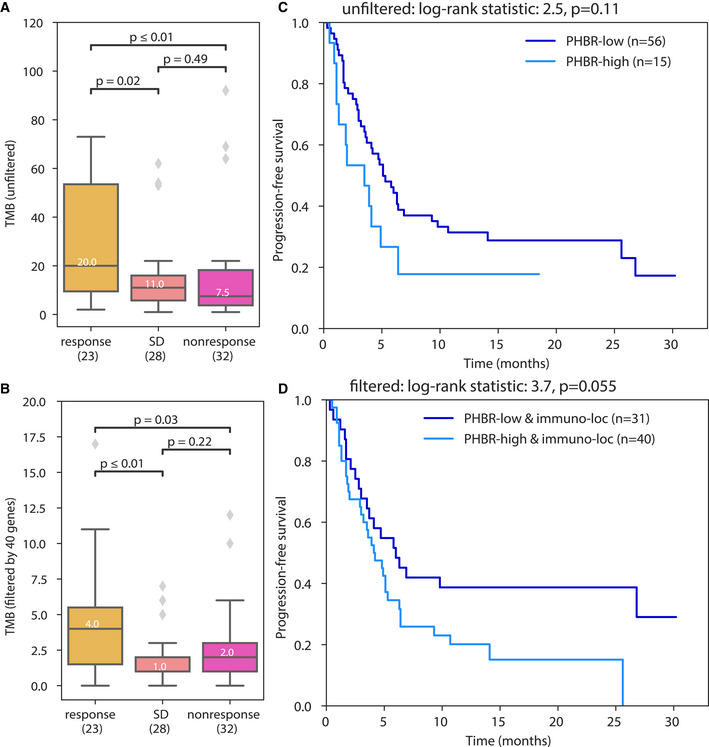
Focusing on immunogenic locations improves response prediction in a gene panel profiled cohort A, BTumor mutation burden focusing on (A) all genes in the gene panel and (B) the 40 genes whose proteins localize to previously observed immunogenic subcellular locations.C, DKaplan–Meier curves showing the effect of the best presented mutation on progression‐free survival (C) using all genes in the panel and (D) using only the 40 genes of interest. Data information: Panels A and B include the median line, the boxes denote the interquartile range (IQR), whiskers denote the rest of the data distribution, and outliers are denoted by points determined by ± 1.5 * IQR. Sample size indicates number of patients. The Mann–Whitney *U* statistical test was performed.

First, we asked whether the burden of somatic mutations in the 40 genes was informative for stratifying patient outcomes. Focusing on mutation burden in proteins from immunogenic locations reduced the total number of mutations under consideration while preserving the potential to distinguish responders from non‐responders and those with stable disease (SD; Fig 4A and B). Second, we asked whether effective presentation of one or more neopeptides from these 40 proteins was a better determinant of outcome than presentation of one or more neopeptides across all proteins in the panel. For this analysis, we focused on the 71 of 83 patients who carried at least one mutation in these 40 genes. While patient MHC genotype‐specific presentation scores (PHBR scores, Marty *et al*, 2017) were able to stratify responders from non‐responders when all proteins were considered (Fig 4C), the stratification improved when we focused on only the 40 proteins from immunogenic locations overall (Fig 4D) and in high TMB patients (Appendix Fig S17). We revisited this analysis using a Cox proportional hazards model with covariates as described previously (Goodman *et al*, 2020), and found that when we focused on the 40 panel genes encoding proteins from immunogenic locations, presentation (PHBR score) was more significantly associated with outcome in high TMB patients, and the model had an improved (lower) Akaike information criterion score (Dataset EV1). Altogether these results support that subcellular location of parent proteins is a determinant of the effective neoantigen burden in the setting of immunotherapy.

## Discussion

While immunotherapy has the potential to generate durable responses (Ledford, 2016), the fraction of patients who respond remains relatively low. Notably, immunotherapy tends to have higher response rates in tumor types with a high burden of somatic mutations, which is thought to be a proxy for having a large number of immunogenic mutations. Mapping the mutations in a tumor genome to the subset that are likely to create immunogenic neoantigens is therefore important to realistically assess the potential for immunotherapy response as well as for designing effective cancer vaccines. Consequently, a variety of metrics have been developed to reveal putative neoantigens in tumor genomes, with the most common being peptide–MHC‐binding affinity, peptide–MHC complex stability, peptide agretopicity, foreignness, and mutation expression. Here, we analyzed peptides from eluted peptide–MHC and found that the subcellular location of proteins also influences which peptides are presented by the MHC. Using a high‐dimensional cellular location embedding that captured multi‐localization mapped to a two‐dimensional representation, we analyzed the implications of parent protein location relative to peptide immunogenicity and immunotherapy response. Immunogenic peptides were biased toward specific subcellular locations and a higher burden of mutations from these regions was associated with more benefit from immunotherapy in multiple cohorts. These findings provide the first evidence that parent protein locations influence both neopeptide presentation and T‐cell recognition and elimination.

We evaluated both the subcellular locations of proteins from which MHC‐I‐ and MHC‐II‐bound peptides originate as well as those associated with peptides labeled as immunogenic based on experimental assays. We note that these locations may not fully overlap. While stable presentation by the MHC is a prerequisite for immunogenicity, it is possible that not all locations from which peptides are sourced generate immunogenic peptides. This is dependent in part on the extent of thymic selection. Furthermore, we note that not all experimental assays used to profile immunogenicity fully recapitulate the dependence on protein location, which could lead to the appearance of some immunogenic peptides coming from regions leading to no peptide presentation by the MHC. Furthermore, there was substantially less information about MHC‐II peptides than MHC‐I, leading to more limited assessment of immunogenicity in locations where peptides are predominantly eluted from MHC‐II. Nonetheless, more MHC‐II presented peptides than MHC‐I presented peptides were associated with vaccine response in multiple vaccine studies (Ott *et al*, 2017; Sahin *et al*, 2017; Hilf *et al*, 2019; Keskin *et al*, 2019).

In general, we speculate that the location constraint could more strongly affect peptide availability for MHC‐I. Peptides from different compartments within the cell may have more variable access to the ER, which depends largely on transport from the cytoplasm by TAP family transporters (Yewdell & Bennink, 1999). Peptides displayed by MHC‐II come mainly but not exclusively from proteins internalized by antigen‐presenting cells. However, MHC‐I and MHC‐II have been found abundantly in exosomes derived from B cells, which may explain the significant enrichment in eluted peptides for both classes I and II (Wubbolts *et al*, 2003; Colombo *et al*, 2014). In addition, the diversity and availability of such proteins could change drastically in the presence of apoptotic or necrotic cells in the tumor immune microenvironment, making proteins from previously unavailable locations more accessible. Cross‐priming may allow some exceptions to location constraints as well (Kurts *et al*, 2010).

These considerations are particularly important in the context of neoantigen vaccines. Effective vaccine design depends on selecting peptides that will induce robust immune responses. Inclusion of peptides that stimulate T‐cell expansion but are not effectively displayed by the MHC at the tumor site creates the risk of generating immunodominance toward ineffective targets (Yewdell & Bennink, 1999). The resulting T‐cell expansions could be dominated by clones incapable of suppressing the tumor, while more relevant clones are outcompeted in competition for antigen on the APCs (Garcia *et al*, 2007), nutrient starved (Kedia‐Mehta & Finlay, 2019), and may become more easily exhausted (Malandro *et al*, 2016). Thus, it may be important to avoid including peptides from parent proteins that are less accessible to the MHC. More stringent constraints on peptide accessibility to MHC‐I might make selection of effective peptides for MHC‐I more challenging than for MHC‐II.

Biases in protein location during thymic selection could render the T‐cell repertoire more sensitive to proteins from certain locations. Therefore, including peptides from these locations could be beneficial. This also leads to the speculation that protein localization changes in tumor cells could alter accessibility to the MHC. If these proteins are less subject to thymic tolerance, they could potentially be more potently immunogenic in the available T‐cell repertoire of adult individuals. One study found that an inverted form of melanoma antigen with altered localization, Melan‐A, was recognized by T cells while the native orientation and a variant expressed in the cytosol were not (Rimoldi *et al*, 2001). Although alterations in localization signals are reportedly rare (Laurila & Vihinen, 2009; Wang & Li, 2014), differences in trafficking could be more common (Tzeng & Wang, 2016). For example, we found that some mitochondrial regions were depleted for immunogenic proteins, however, mitochondrial‐derived vesicles may provide a pathway to the MHC to proteins from these regions (Matheoud *et al*, 2016).

We note several limitations to our study. The pre‐trained location embeddings were based on characteristics of normal cells, and will reflect any biases or gaps present in the gene ontology (Gaudet & Dessimoz, 2017). Furthermore, many proteins map to multiple locations (Thul *et al*, 2017) and have multiple associated cellular component terms. In this study, we weighted each component equally, but it is likely that some locations may be predominant or transient. Immunogenicity is based on experimental assays in the IEDB performed on 325 proteins by various groups using various assays. These proteins could reflect selection bias. Similarly, locations associated with MHC‐eluted peptides may reflect the specific alleles that were profiled. In addition, MHC‐II datasets may be biased toward B cells, whereas differences in internalization mechanisms among antigen‐presenting cell types such as dendritic cells or macrophages could create differences in which proteins are more accessible.

Despite these limitations, we found that incorporating protein location into analysis of immunotherapy cohorts was helpful in several ways. We used location to revise the effective neoantigen burden in tumors and better stratify potential for immunotherapy response, although the best performance was observed in tumor types similar to the training data, namely melanoma, lung, and bladder, as well as datasets with higher overlap in the locations of the source proteins studied. Studying the effects of location in the context of tumor immunoediting is further made difficult by patterns of co‐segregating mutation, and subclone‐specific mechanisms of immune evasion can confound the association with neoantigen characteristics. More insight may be gained from future single‐cell studies where it is possible to define the clonal architecture of tumors and determine which mutations coexist within the same clones. Indeed, preprint: Mehrabadi *et al* (2021) found that location bias of mutated proteins correlated with immunoediting of specific tumor subclones in a murine model of melanoma. Location information was also beneficial in a cohort that was profiled with a gene panel, suggesting that this information could still be relevant for the more limited data commonly generated in clinical settings. Thus, we conclude that protein subcellular location contributes to shaping the tumor–immune interface and can potentially be leveraged to improve the effective application of immunotherapies.

## Materials and Methods

### GO analysis

Gene ontology enrichment analysis was performed using GOATOOLS (https://github.com/tanghaibao/goatools; Klopfenstein *et al*, 2018) using the standard parameters, and retaining enriched or depleted results if the Benjamini–Hochberg corrected *P*‐value was < 0.05 (Dataset EV2).

### Abelin 2019 peptides

Peptides were obtained from the published data from Abelin *et al* (2019). Peptides were mapped to parent UniProt sequences and filtered out if they mapped to multiple parent proteins. A total of 69,653/76,561 (90.7%) peptides were uniquely mapped to one parent protein sequence.

### Isolation and purification of HLA‐DR‐bound peptides

The human B‐cell lymphoblastoid cell lines 721.221, JThom (9004), OLL (9100), and SPACHECO (9072) were grown in complete RPMI 1640 medium (Gibco) supplemented with 10% fetal bovine serum (FBS; Gibco/Invitrogen Corp). The HeLa cell line was grown in DMEM/F12K (Gibco) supplemented with 10% fetal bovine serum (FBS; Gibco/Invitrogen Corp). The cells were grown in large‐scale cultures in roller bottles and the cell viability was maintained at > 90% throughout the experiments. To induce HLA Class II surface expression, HeLa cells were treated with IFNɣ (500 U/ml) for 72 h after which the cells were harvested, washed twice with ice‐cold PBS, and spun down at 2,500 *g* for 10 min. The cell pellets were snap frozen in LN_2_ and stored at −80 until downstream processing. All cell lines were subjected to high‐resolution sequence‐based HLA typing (HLA‐A, ‐B, ‐C, DR, DP, and DQ) for authentication prior to large‐scale culture and data collection.

HLA‐DR molecules were purified from the cells by affinity chromatography using the anti‐human HLA‐DR antibody (clone L243) coupled to CNBr‐activated Sepharose 4 Fast Flow (Amersham Pharmacia Biotech, Orsay, France) as described previously (Kaabinejadian *et al*, 2022). Briefly, frozen cell pellets were pulverized using Retsch Mixer Mill MM400, resuspended in lysis buffer comprised of Tris pH 8.0 (50 mM), Igepal, 0.5%, NaCl (150 mM), and complete protease inhibitor cocktail (Roche, Mannheim, Germany). Lysates were centrifuged in an Optima XPN‐80 ultracentrifuge (Beckman Coulter, IN, USA) and filtered supernatants were loaded on immunoaffinity columns. After a minimum of three passages, columns were washed sequentially with a series of wash buffers (Purcell *et al*, 2019) and were eluted with 0.2 N acetic acid. The HLA was denatured, and the peptides were isolated by adding glacial acetic acid and heat. The mixture of peptides and HLA‐DR was subjected to reverse‐phase high‐performance liquid chromatography (RP‐HPLC).

### Fractionation of the HLA/peptide mixture by RP‐HPLC

Reverse‐phase high‐performance liquid chromatography was used to reduce the complexity of the peptide mixture eluted from the affinity column. First, the eluate was dried under vacuum using a CentriVap concentrator (Labconco, Kansas City, Missouri, USA). The solid residue was dissolved in 10% acetic acid and fractionated using a Paradigm MG4 instrument (Michrom BioResources, Auburn, California, USA). An acetonitrile (ACN) gradient was run at pH 2 using a two‐solvent system. Solvent A contained 2% ACN in water, and solvent B contained 5% water in ACN. Both solvent A and solvent B contained 0.1% trifluoroacetic acid (TFA). The column was pre‐equilibrated at 2% solvent B. Then, the sample was loaded at a flow rate of 120 μl/min and a two‐segment gradient was run at 160 μl/min flow rate as described in detail in Kaabinejadian *et al* (2022). Fractions were collected in 2 min intervals using a Gilson FC 203B fraction collector (Gilson, Middleton, Wisconsin, USA), and the ultra‐violet (UV) absorption profile of the eluate was recorded at 215 nm wavelength.

### Nano‐LC–MS/MS analysis

Peptide‐containing HPLC fractions were dried, resuspended in a solvent composed of 10% acetic acid, 2% ACN, and iRT peptides (Biognosys, Schlieren, Switzerland) as internal standards. Fractions were applied individually to an Eksigent nanoLC 415 nanoscale RP‐HPLC (AB Sciex, Framingham, Massachusetts, USA), including a 5‐mm‐long, 350 μm of internal diameter Chrom XP C18 trap column with 3 μm particles and 120 Å pores, and a 15‐cm‐long ChromXP C18 separation column (75 μm internal diameter) packed with the same medium (AB Sciex, Framingham, Massachusetts, USA). An ACN gradient was run at pH 2.5 using a two‐solvent system. Solvent A was 0.1% formic acid in water, and solvent B was 0.1% formic acid in 95% ACN in water. The column was pre‐equilibrated at 2% solvent B. Samples were loaded at 5 μl/min flow rate onto the trap column and run through the separation column at 300 nl/min with two linear gradients: 10–40% B for 70 min, followed by 40–80% B for 7 min.

The column effluent was ionized using the nanospray III ion source of an AB Sciex TripleTOF 5600 quadrupole time‐of‐flight mass spectrometer (AB Sciex, Framingham, MA, USA) with the source voltage set to 2,400 V. Information‐dependent analysis (IDA) method was used for data acquisition (Kaabinejadian *et al*, 2022). PeakView Software version 1.2.0.3 (AB Sciex, Framingham, MA, USA) was used for data visualization.

### Peptide identification and source protein information

Peptide sequences were identified using PEAKS Studio 10.5 software (Bioinformatics Solutions, Waterloo, Canada). A database composed of SwissProt Homo sapiens (taxon identifier 9606) and iRT peptide sequences was used as the reference for database search. Variable post‐translational modifications (PTM) including acetylation, deamination, pyroglutamate formation, oxidation, sodium adducts, phosphorylation, and cysteinylation were included in database search. Identified peptides were further filtered at a false discovery rate (FDR) of 1% using PEAKS decoy‐fusion algorithm.

### Cellular component location embedding

Gene ontology (GO) cellular component (CC) annotations for all UniProt protein IDs was obtained from uniprot.org. Pre‐trained 200‐dimensional vectors for 64,649 GO terms were obtained from Kim *et al* (2021). Vectors were mapped to UniProt IDs and summed if a UniProt ID had more than one associated GO CC term. UMAP dimensionality reduction (preprint: McInnes *et al*, 2018) using the “hyperboloid” metric was applied, then mapped to the Poincare disk model. The resulting two values for each protein were used as features.

### Prediction of elution using location and expression features

*Dataset*: Eluted peptides from 721.221 B cells (*n* = 3,510) with matched expression (discussed in Appendix Figs S1–S4).

*Method*: We retained only one parent protein for observed/eluted peptides, resulting in *n* = 638 positive labels and *n* = 17,307 negative labels (the remaining proteins for which we had expression values) for eluted proteins. We trained a random forest model using these data with the parameters described in the “Random forest model” section (see below) to account for an imbalanced dataset.

### IEDB data

Peptides were selected from the Immune Epitope Database and Analysis Resource (www.iedb.org; Vita *et al*, 2019) on July 16, 2021, using filters “Epitope Structure: Linear Sequence,” “Included Related Structures: neo‐epitope,” “No B cell Assays,” “No MHC assays,” “MHC Restriction Type: Class I,” “Host: Homo sapiens (human)” and “Include Positive Assays,” and “Include Negative Assays” (T‐cell assays). This resulted in 3,754 peptides. Peptides whose “Assay Antigen Antigen Description” sequence did not match “Epitope Description” sequence were filtered, resulting in 3,521 peptides. Peptides were also filtered out if they did not have an associated UniProt ID found in the “Related Object Parent Protein IRI” field, resulting in 3,367 peptides. Peptides were additionally removed if they were not in the UniProt CC file (downloaded on April 17, 2020, from uniprot.org by selecting all human peptides and choosing “Gene ontology (cellular component)” in the column selection; see *Cellular Component Location Embedding*), resulting in 3,125 peptides. Peptides were dropped if they did not have a specific associated HLA allele (e.g., Allele Name = “HLA class I” or Allele Name = “HLA‐A2”) or if they were not a simple linear sequence (e.g., ILCETCLIV + AIB (C3, C6)). This resulted in 2,943 peptides. Affinity, stability, and foreignness features were calculated as described in “Validation Data.”

Hex plots: Parent protein locations were plotted for each unique protein and immunogenic state for each study (i.e., if peptides from Protein A had positive and negative tests, the parent protein would be retained twice, once in each immunogenic category).

Clustering of UMAP location features: We clustered the UMAP location values using a K‐means model specifically designed for the hyperbolic space (Popoff). We used the elbow method to select seven clusters. We then annotated each cluster with gene ontology (GO) cellular components using default parameters (Ashburner *et al*, 2000; Mi *et al*, 2019; Gene Ontology Consortium, 2021).

### Random forest model

The RandomForestClassifier from sklearn v0.24.2 was trained using random state 2021. The StratifiedKFold function was used to perform the 10‐fold splits, also using random state 2021. The Youden indices for each fold were obtained by taking the threshold that had the greatest TPR‐FPR (i.e., greatest area under the curve). The median Youden index was used to classify peptides as immunogenic or not for downstream analyses.

### Wells data

Experimentally validated peptides were obtained from published appendix tables S4 and S7 in Wells *et al* (2020). Peptides were mapped to parent proteins by iterating through all UniProt proteins and looking for a match to any peptide with a wildcard in the given mutated position (e.g., Python code to find the wild‐type peptide corresponding to “FLCEILRSMSI” with mutated position 10: re.findall(r'? = (“FLCEILRSM.I”)), protein_sequence). Ten peptides without a mutated position were excluded. A total of 584 (97.6%) of neopeptide sequences were mapped to one unique parent wild‐type sequence. Peptides with matched wild‐type sequences mapping to multiple UniProt IDs were dropped. Missing foreignness or agretopicity scores were re‐calculated using the methods described in Wells *et al* The resulting 558 peptides (Dataset EV3) from the discovery set were used to train a Random Forest classifier using sklearn (v0.24.2).

### Wells validation data

The trained model was tested on the 310 peptides validation dataset from Wells *et al*, as well as 43 peptides from ovarian tumors (Liu *et al*, 2019b). As these datasets did not include all features from the discovery dataset, NetMHCstabpan (v1.0; Rasmussen *et al*, 2016) was used to predict peptide–MHC stability, NetMHCpan (v4.0; Jurtz *et al*, 2017) was used to predict peptide–MHC‐binding affinity, and the antigen.garnish package (https://github.com/andrewrech/antigen.garnish) was used to calculate foreignness as described in Luksa, Wells. Finally, agretopicity was calculated by taking the ratio of mutant‐to‐wild‐type–binding affinity.

### Validation immunotherapy cohorts

For cohorts with provided neoepitope data analyzed in respective original manuscripts (Snyder *et al*, 2014, 2017; Rizvi *et al*, 2015; Van Allen *et al*, 2015), we re‐calculated affinity with NetMHCpan (v4.0), stability with NetMHCstabpan (v1.0), and foreignness with the antigen.garnish package as described above, and annotated location. For cohorts without neoepitope data provided (Hugo *et al*, 2017; Miao *et al*, 2018; Liu *et al*, 2019), we performed the same calculations for all HLA alleles and designated the best presented neopeptide for each mutation as the neoepitope. We utilized the original labels of responder/non‐responder from each respective manuscript without extra grouping. For example, we kept the three separate groupings from the Van Allen cohort: “responders,” “long‐term survival with no clinical benefit,” and “nonresponders.”

## Author contributions

**Andrea Castro:** Conceptualization; data curation; software; formal analysis; validation; investigation; visualization; methodology; writing – original draft; writing – review and editing. **Saghar Kaabinejadian:** Resources; formal analysis; validation; methodology. **Hooman Yari:** Resources; data curation; formal analysis; validation; methodology. **William Hildebrand:** Resources; supervision; funding acquisition; project administration. **Maurizio Zanetti:** Resources; supervision; funding acquisition; methodology; writing – original draft; project administration; writing – review and editing. **Hannah Carter:** Resources; supervision; funding acquisition; visualization; methodology; writing – original draft; project administration; writing – review and editing.

## Disclosure and competing interests statement

SK is an employee at Pure MHC, LLC.

## Supporting information



Appendix

Dataset EV1

Dataset EV2

Dataset EV3

## Data Availability

The codes generated in this study have been deposited on GitHub (https://github.com/cartercompbio/Ploc).
