# Supplementary material for: Subcellular location of source proteins improves prediction of neoantigens for immunotherapy
Source: EMBO J. 2022 Oct 31;41(24):e111071. doi: 10.15252/embj.2022111071 (PMC9753441; doi:10.15252/embj.2022111071)
Supplement: Supplementary file 1 — Appendix [file EMBJ-41-e111071-s002.pdf]

Table of Contents:

Appendix Figure S1: Overview of enrichment or depletion of cellular components in multiple datasets.
Appendix Figure S2: Correlation of gene expression and eluted peptide location.
Appendix Figure S3: Relationship between protein turnover and elution for the top 20 most frequently enriched or depleted cellular components across evaluated tissues or cell lines.
Appendix Figure S4: Hexplot of UMAP location embeddings for all unique UniProt proteins with reviewed status and unique gene names.
Appendix Figure S5: Boxplot comparing the frequency of eluted peptides versus immunogenicity.
Appendix Figure S6: Hexplot visualization of embedded location for neopeptides from the IEDB.
Appendix Figure S7: Comparison of location and other peptide features between immunogenic and non-immunogenic peptides from the IEDB.
Appendix Figure S8: Random forest model including GTEx median gene expression as a variable.
Appendix Figure S9: Comparison of the IEDB and Wells *et al*. datasets.
Appendix Figure S10: Overview of MHC characteristics for immunogenic peptides in the IEDB and Wells datasets.
Appendix Figure S11: AUROC and AUPRC plots for the model trained on the Wells discovery dataset and tested on the Wells test dataset.
Appendix Figure S12: Comparison of the IEDB and Liu *et al*. datasets.
Appendix Figure S13: Testing pretrained models on the unseen Liu ovarian dataset.
Appendix Figure S14: Analysis of neopeptide vaccine parent protein MHC elution patterns.
Appendix Figure S15: Comparison of neopeptide characteristics in the Riaz *et al*. dataset.
Appendix Figure S16: Fraction of putative neoantigens removed after filtering by location in two analyses.
Appendix Figure S17: Kaplan Meier curves showing the effect of the best presented mutation on progression free survival.

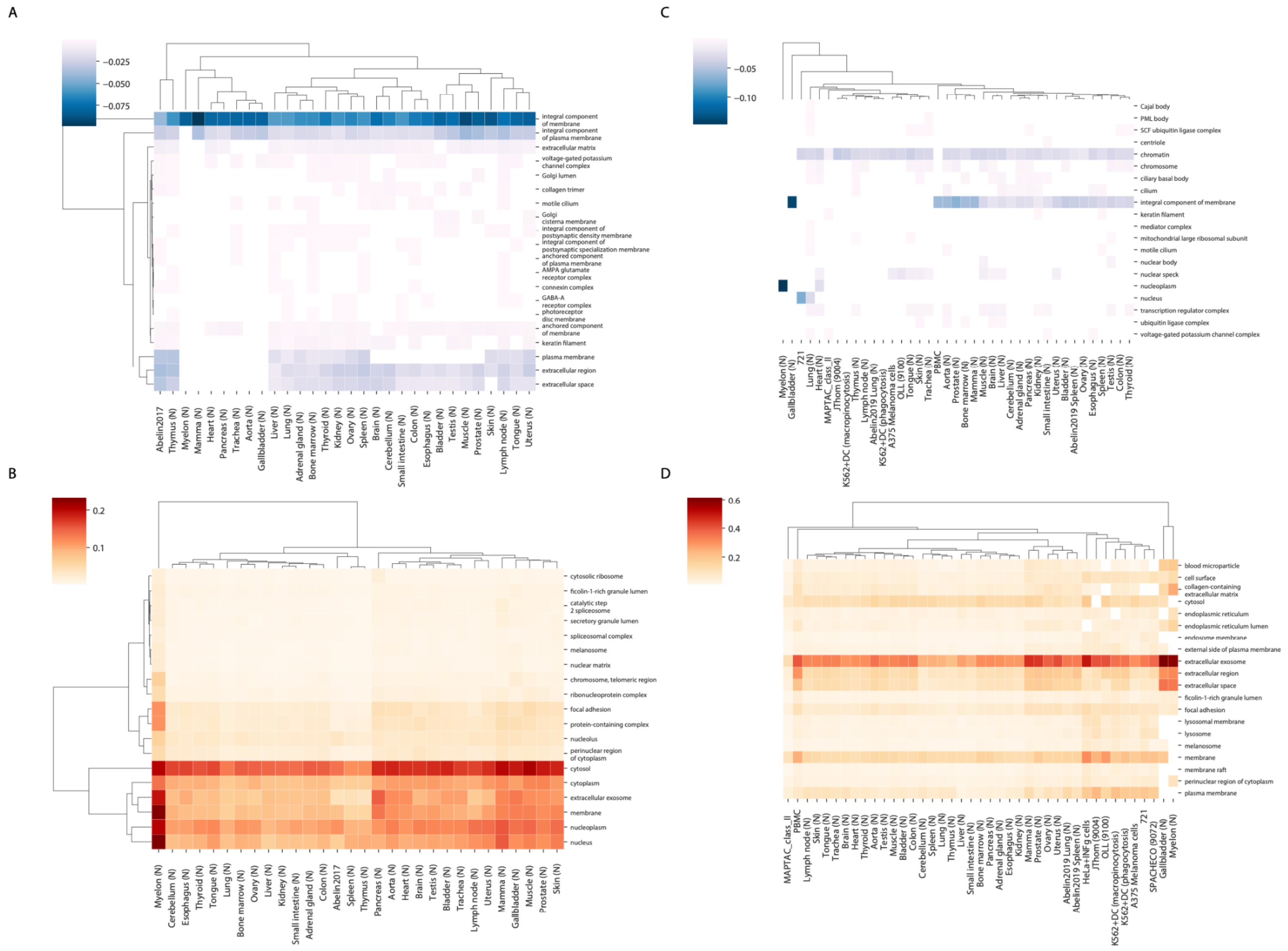

Appendix Figure S1. Overview of enrichment or depletion of cellular components in multiple datasets. (A) Clustermap of the top 20 cellular components depleted in eluted peptide-MHC (pMHC) class I from normal and cell lines. (B) Clustermap of 21 cellular components enriched in eluted pMHC-I complexes across all evaluated normal tissues, indicated by “(N)” (Marcu et al., 2021) and evaluated cell lines (Abelin et al., 2017). The color indicates the difference in study vs population enrichment. Clustermaps of (C) depleted and (D) enriched cellular components for eluted pMHC-II from 721.221, JThom (9004), OLL (9100), and SPACHECO (9072) B cells, HeLa cells stimulated with IFN-γ, (Abelin et al., 2019), and (Abelin et al., 2019; Marcu et al., 2021).

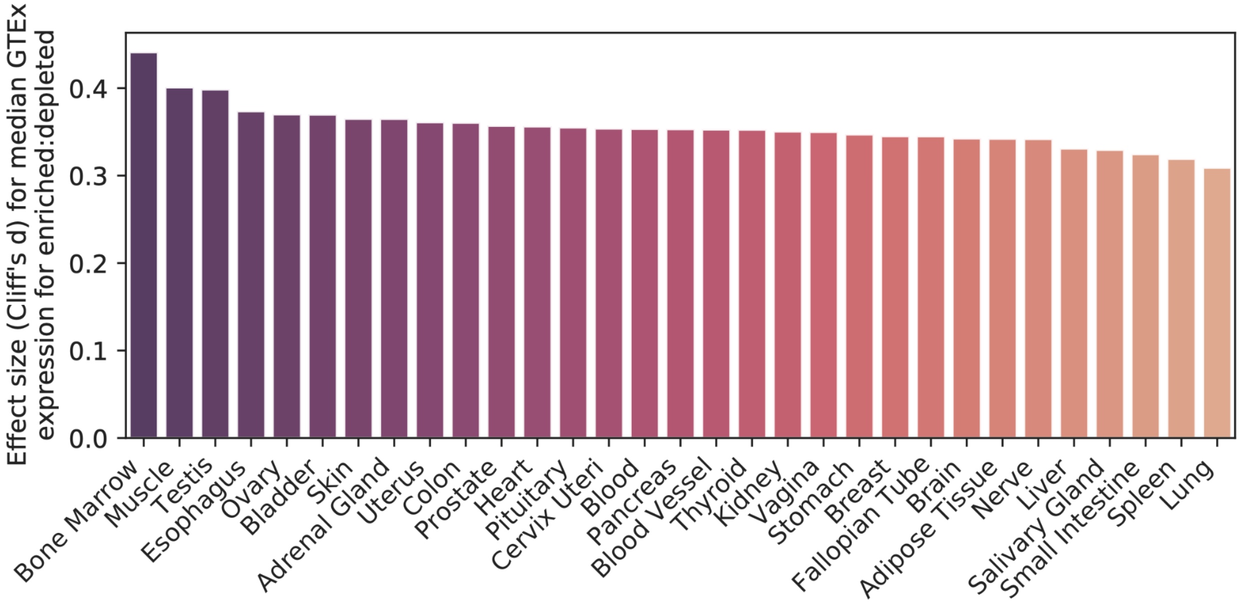

Appendix Figure S2. Correlation of gene expression and eluted peptide location. Barplot for each tissue showing the effect size comparing the median GTEx gene expression for enriched over depleted genes. All comparisons show that genes in enriched locations have higher median gene expression than genes in depleted locations.

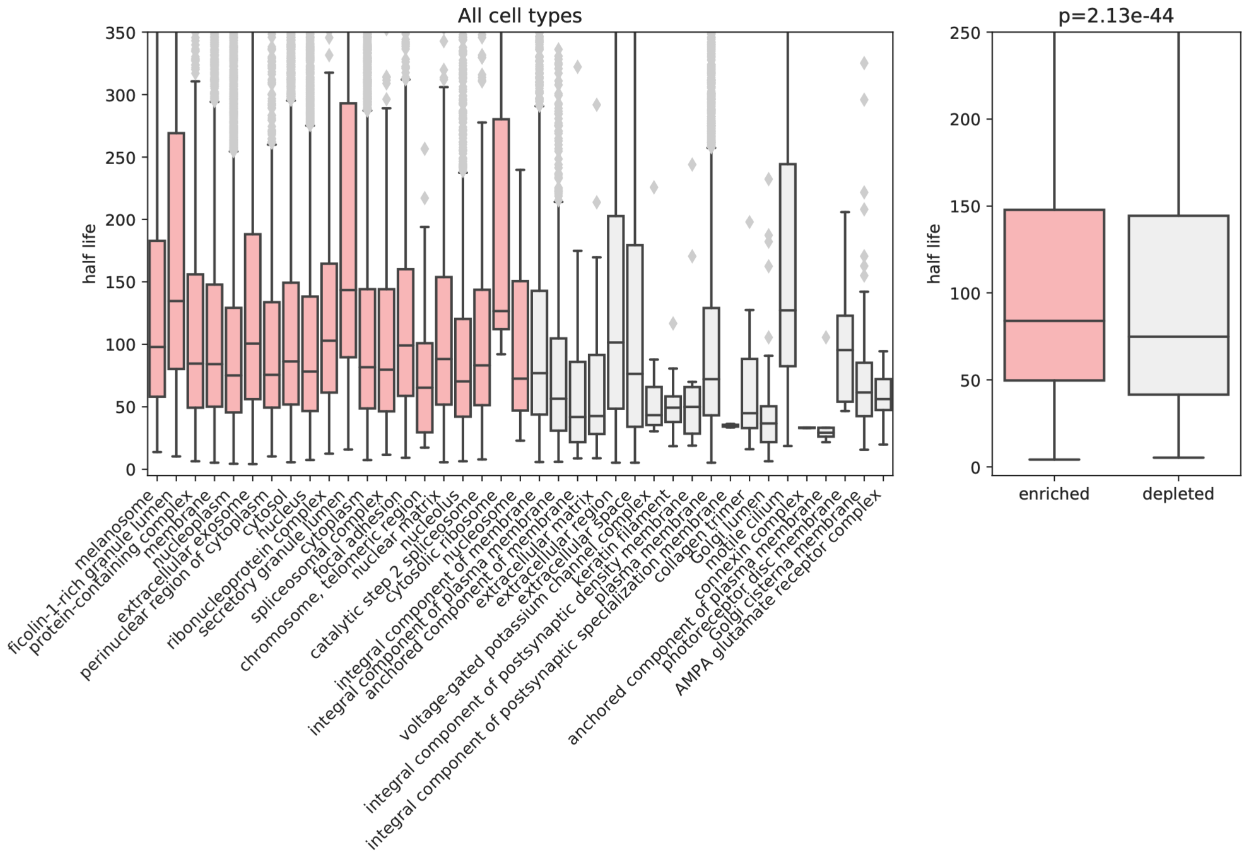

Appendix Figure S3. Relationship between protein turnover and elution for the top 20 most frequently enriched or depleted cellular components across evaluated tissues or cell lines. The Mann-Whitney U test was used to compare statistical significance.

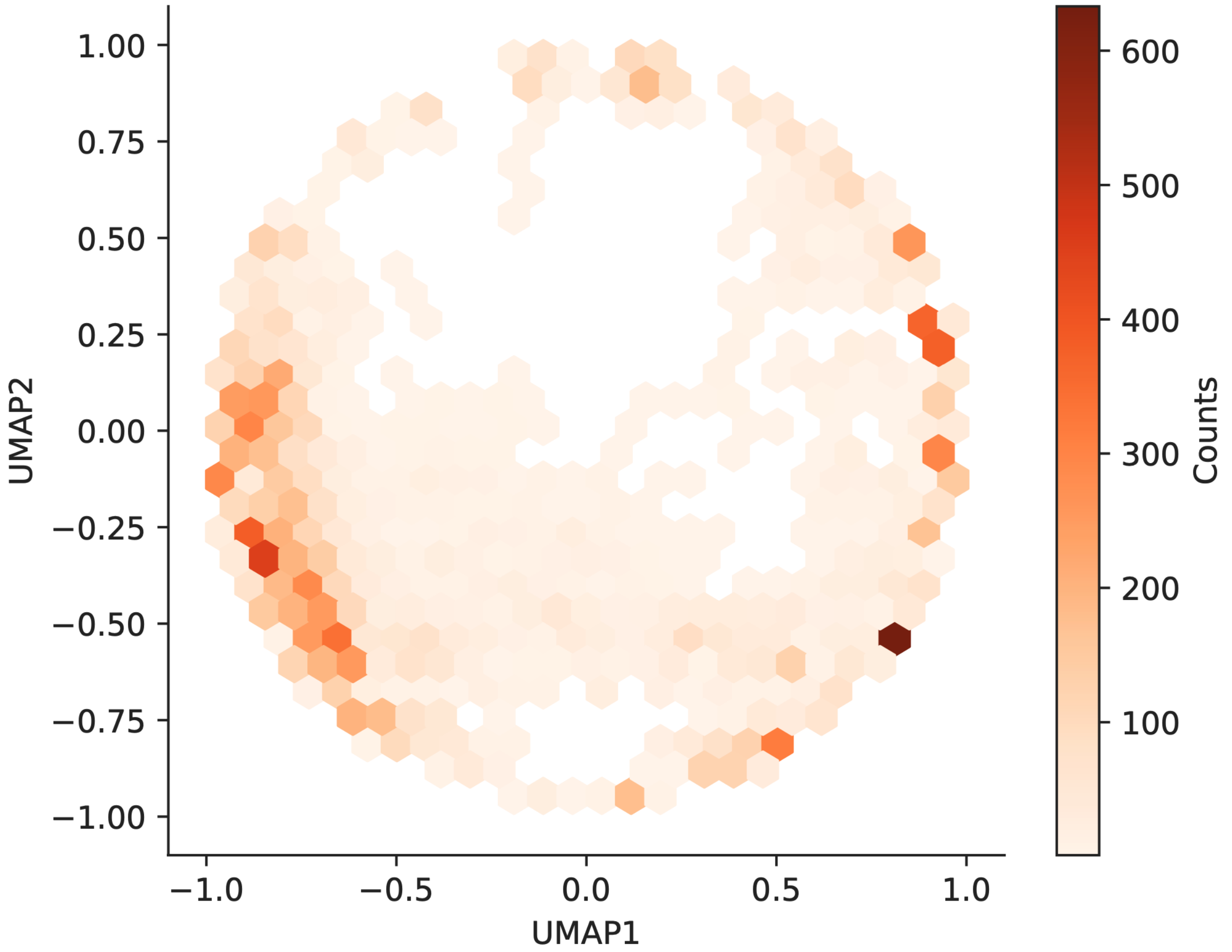

Appendix Figure S4. Hexplot of UMAP location embeddings for all unique UniProt proteins with reviewed status and unique gene names.

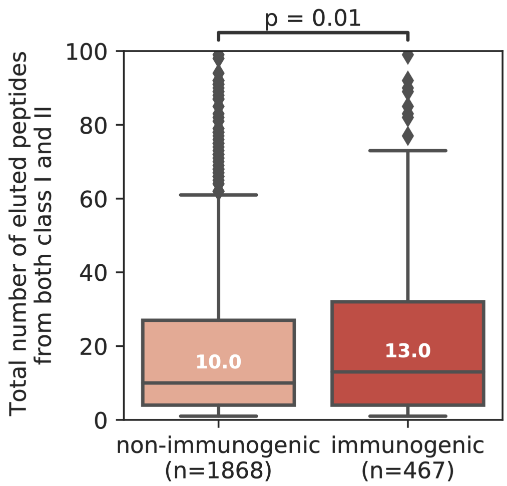

Appendix Figure S5. Boxplot comparing the frequency of eluted peptides versus immunogenicity assay results for proteins that have been evaluated for class I immunogenicity in the IEDB.

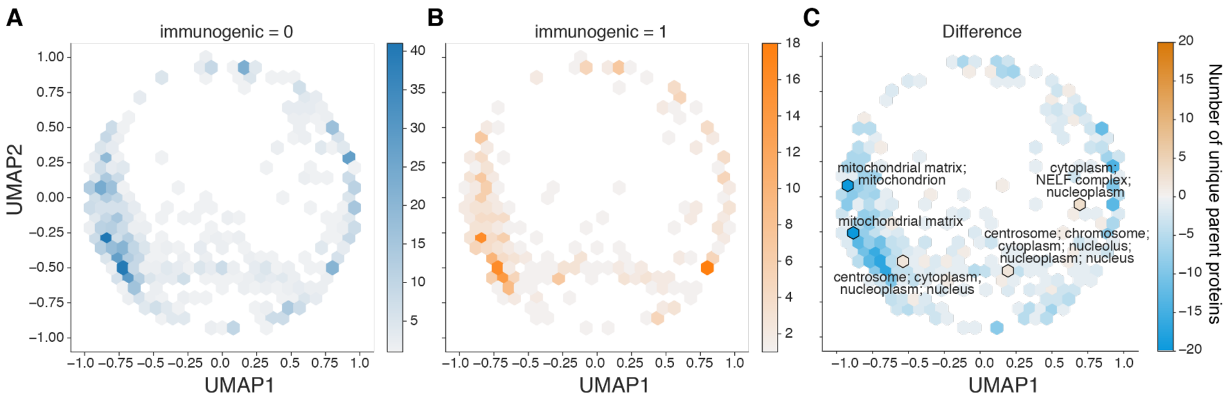

Appendix Figure S6. Hexplots of embedded location for (A) non-immunogenic (blue) and (B) immunogenic (orange) peptides. (C) Hexplot depicting the difference between immunogenic and non-immunogenic hexplots in A and B. Orange indicates more immunogenic peptides, and blue indicates more non-immunogenic peptides.

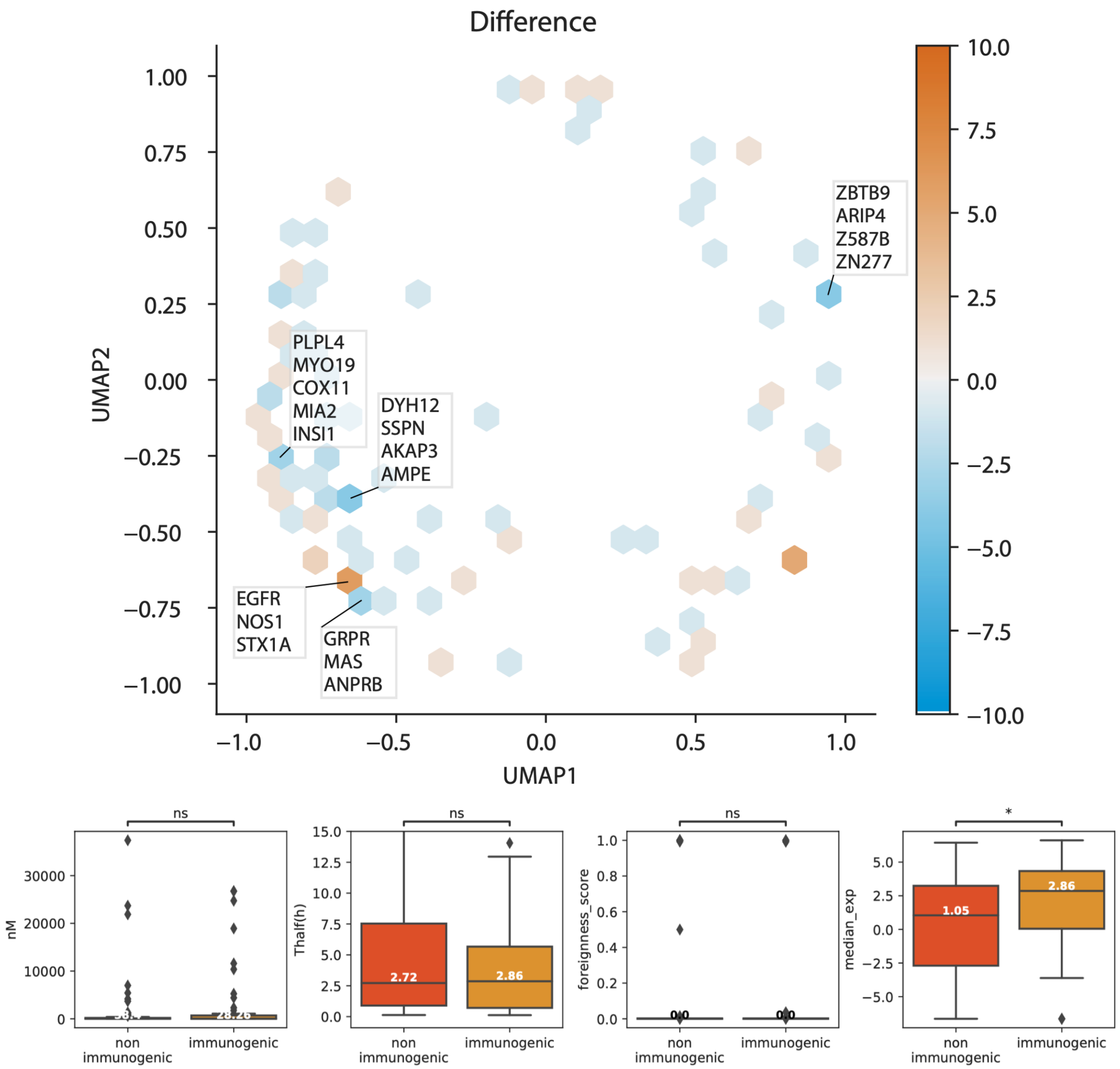

Appendix Figure S7. (Top) Overview of differentially classified peptides between the models with and without location as a feature. Orange indicates locations with more immunogenic peptides compared to non-immunogenic peptides and vice versa. Locations with more than 3 immunogenic or non-immunogenic genes are highlighted. (Bottom) Boxplots comparing peptide-MHC affinity (nM), stability (Thalf (h)), foreignness scores, and median GTEx expression distributions between non-immunogenic (red) and immunogenic (gold) peptides. The Mann-Whitney U statistical test was performed.

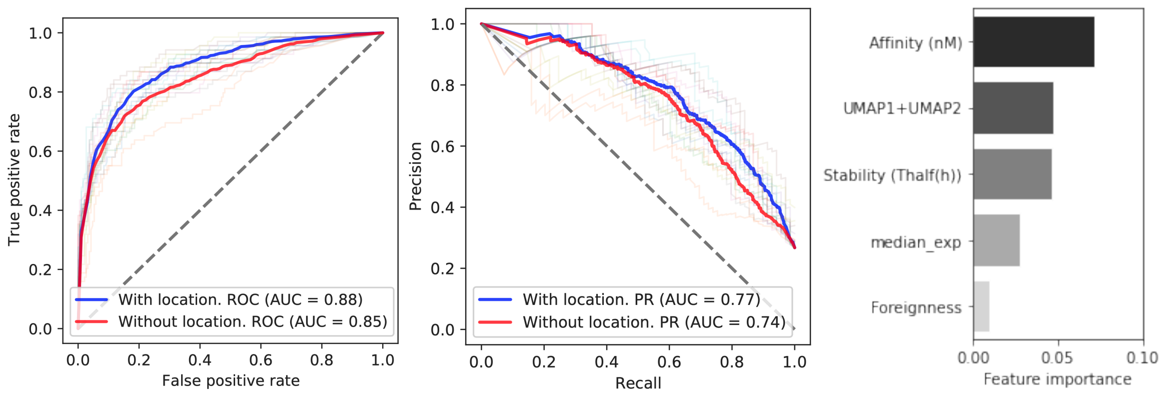

Appendix Figure S8. (Left) Area under the receiver operating characteristic curve (AUROC) and (Middle) area under the precision recall curve (AUPRC) for 10-fold cross validation using a Random Forest model incorporating median GTEx gene expression, peptide affinity, stability, and foreignness (Methods) with and without parent protein location features. (Right) Barplot denoting respective feature importances for the model.

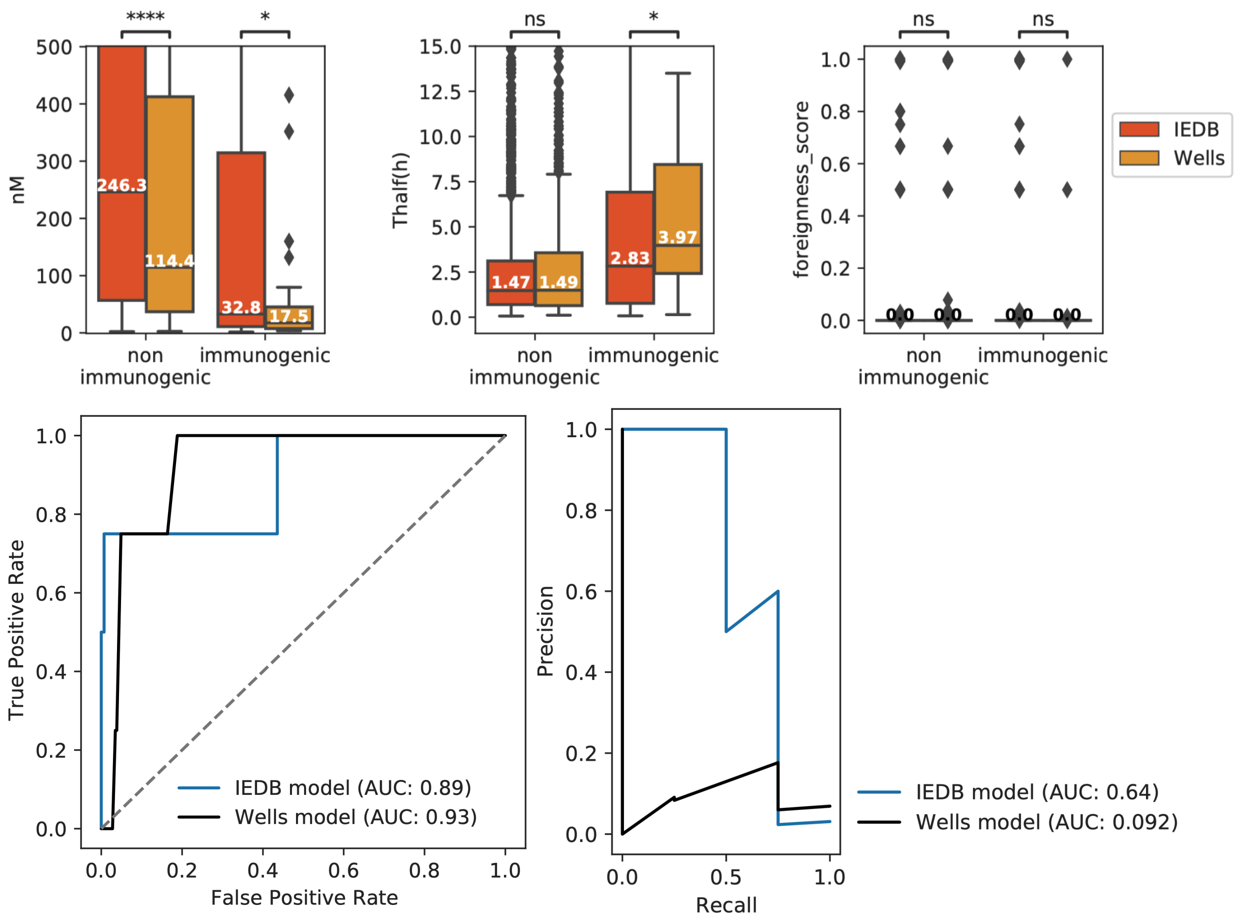

Appendix Figure S9. Comparison of the IEDB and Wells *et al*. datasets. (Top) Boxplots comparing affinity (measured in nM), stability (measured by half life), and foreignness stratified by immunogenicity. The Mann-Whitney U test was used to compare statistical significance. (Bottom panel) Area under the receiver operating characteristic and precision recall curves using the random forest model trained on the IEDB dataset and Wells discovery set to test on the Wells test set.

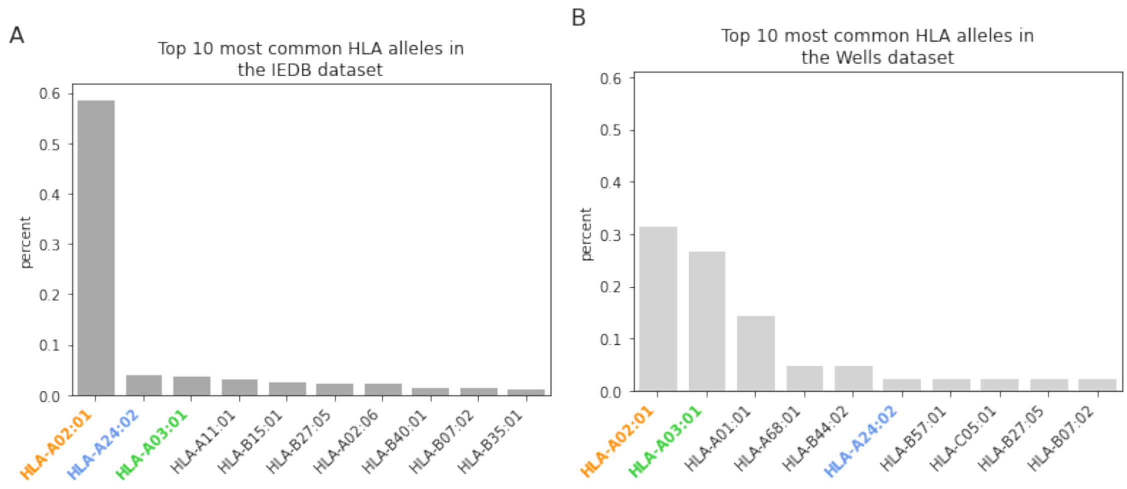

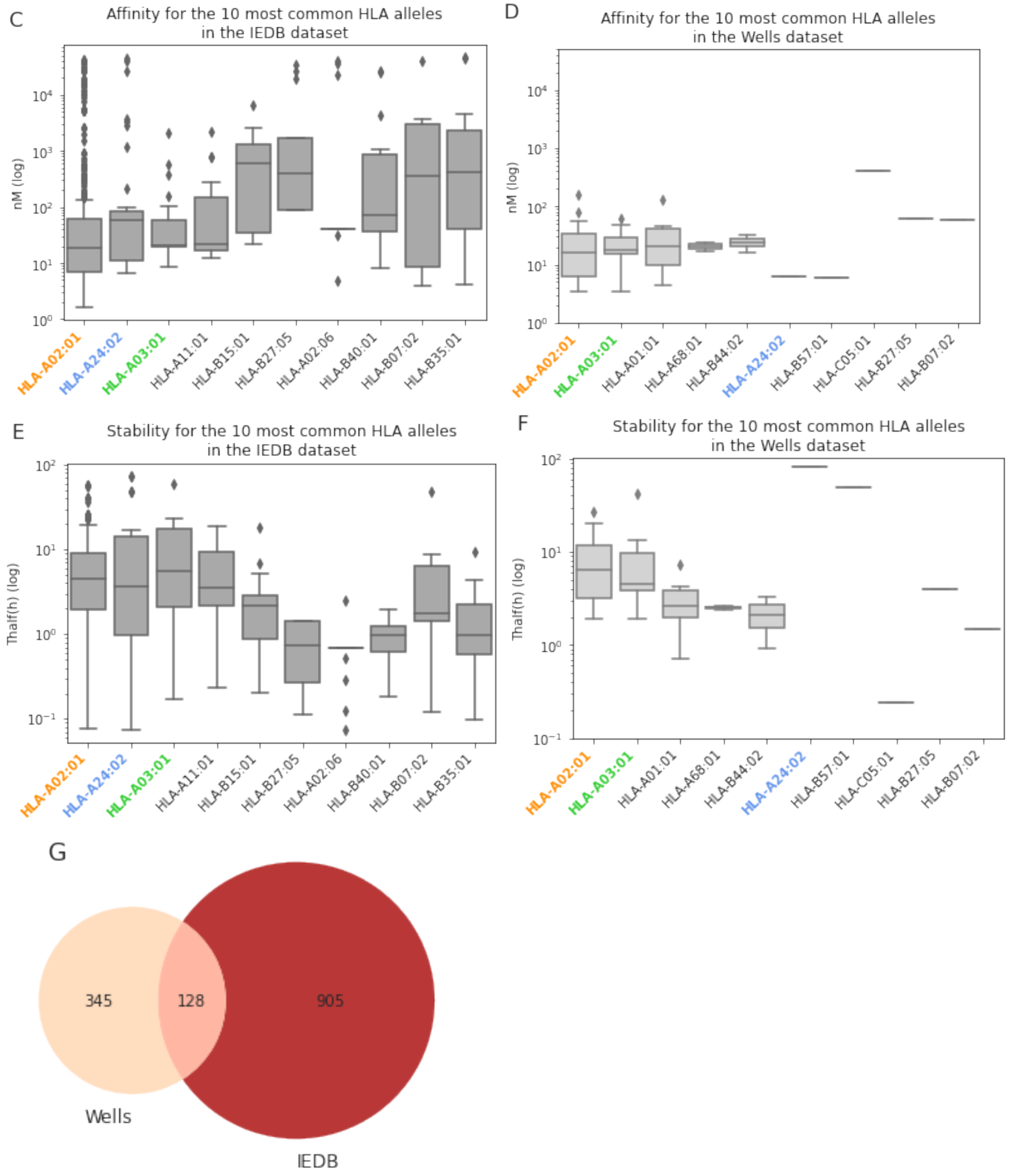

Appendix Figure S10. Overview of MHC characteristics for immunogenic peptides in the IEDB (left column) and Wells datasets (right column). Barplots denoting the frequencies of the 10 most common HLA alleles in the (A) IEDB and (B) Wells dataset that present immunogenic peptides. Boxplots showing the distribution of affinity (log nM) for these top 10 HLA alleles in the (C) IEDB and (D) Wells dataset. Boxplots showing the distribution of stability (log Thalf(h)) for these top 10 HLA alleles in the (E) IEDB and (F) Wells dataset. (G) Venn diagram showing the intersection of shared locations for parent proteins of peptides evaluated in the IEDB and Wells datasets.

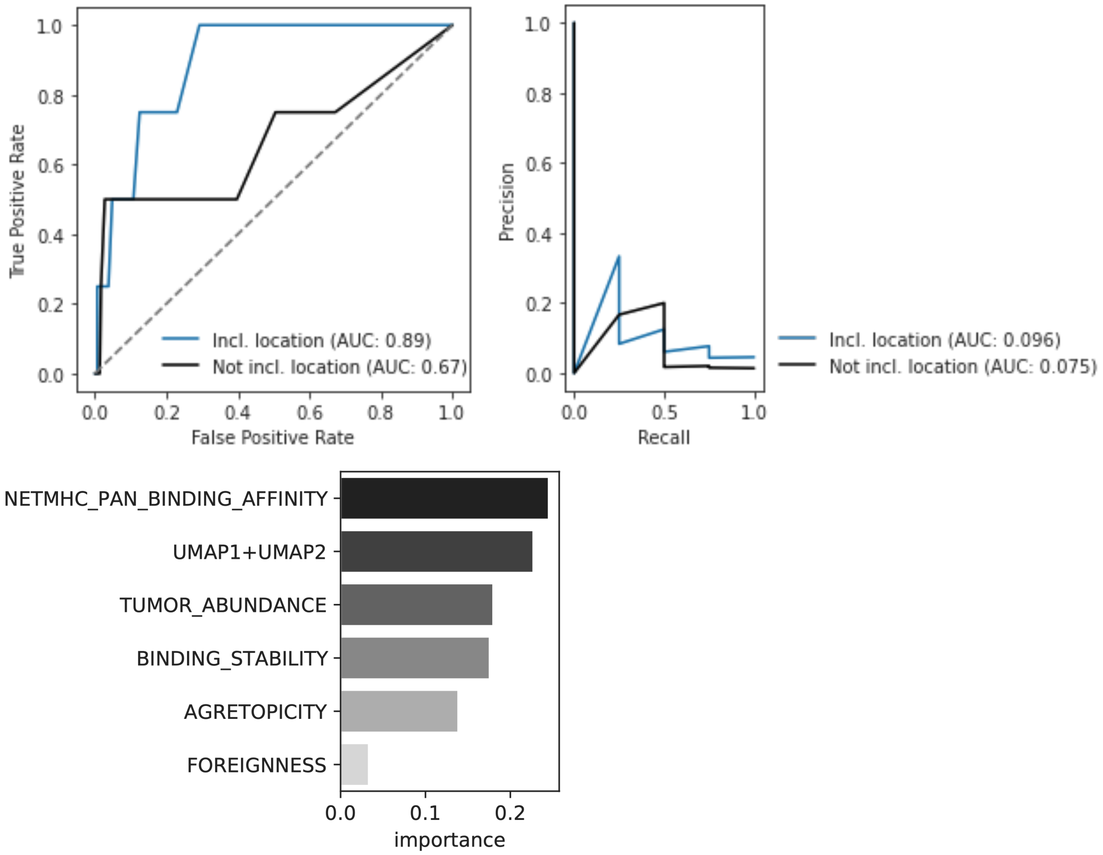

Appendix Figure S11. AUROC and AUPRC plots for the model trained on the Wells discovery dataset and tested on the Wells test dataset. Features include peptide affinity, stability, tumor abundance, agretopicity, and foreignness. Barplot denoting feature importance for the model.

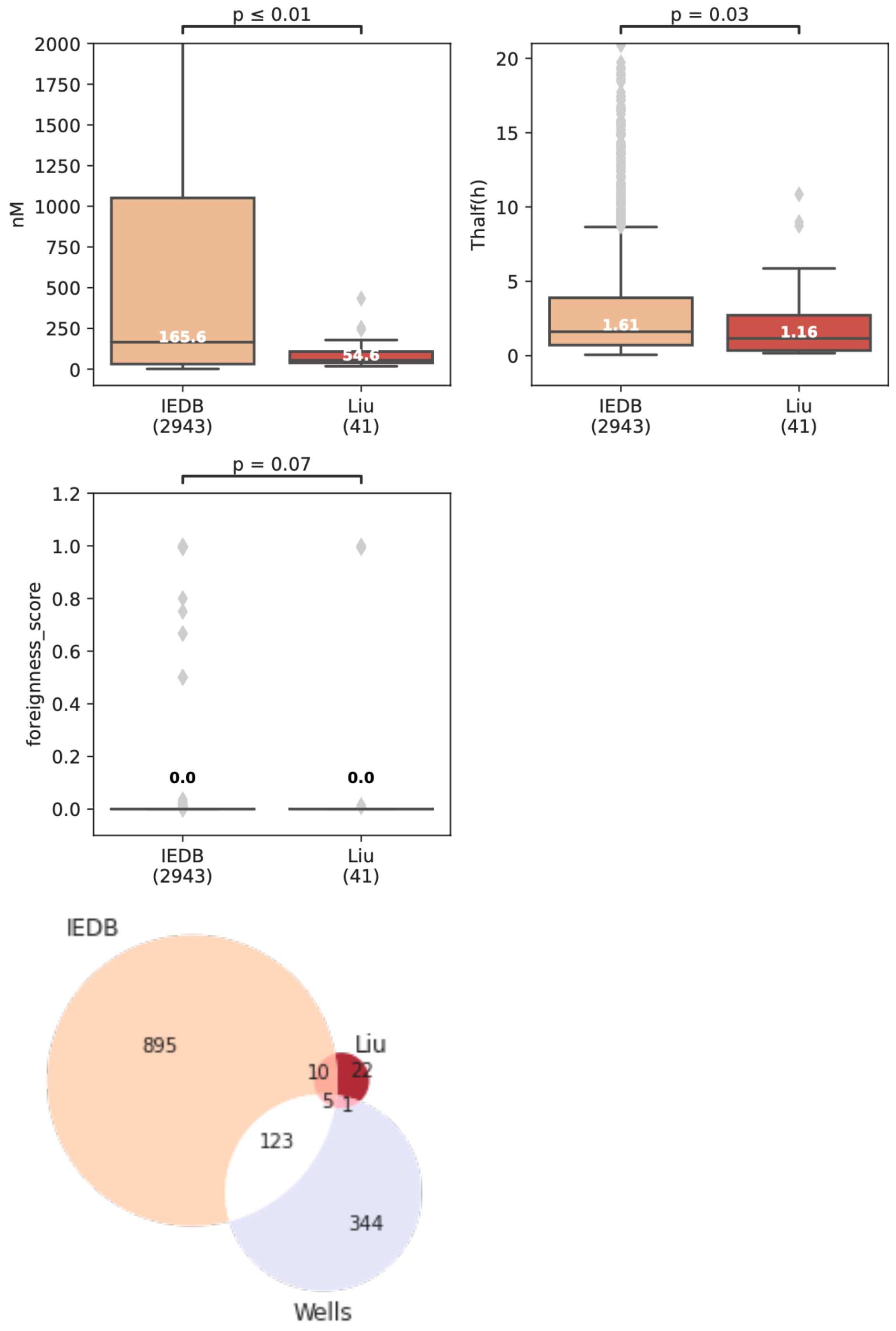

Appendix Figure S12. Comparison of the IEDB and Liu *et al*. datasets. Boxplots comparing affinity (measured in nM), stability (measured by half life), and foreignness. The Mann-Whitney U test was used to compare statistical significance. The Venn diagram shows the overlapping unique locations. All overlapping locations were non-immunogenic in Liu.

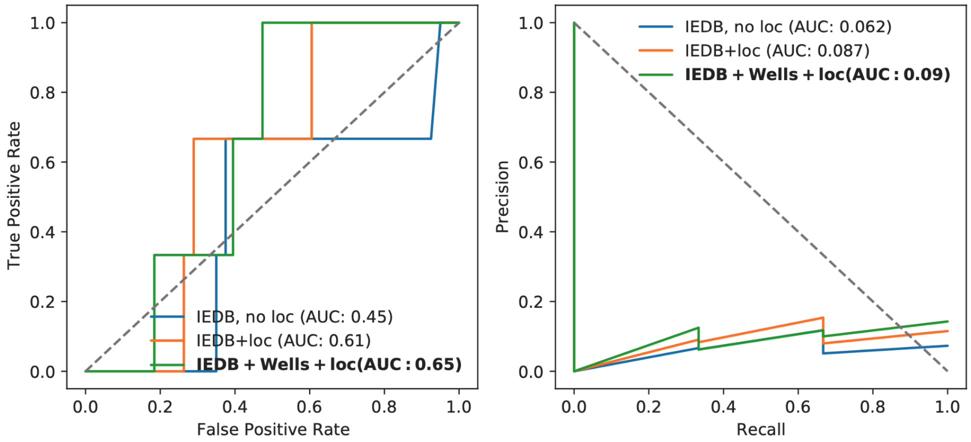

Appendix Figure S13. Testing pretrained models on the unseen Liu ovarian dataset. (Left) AUROC and (right) AUPRC curves for the IEDB model without location, with location, and aggregated model with IEDB, Wells et al., and location.

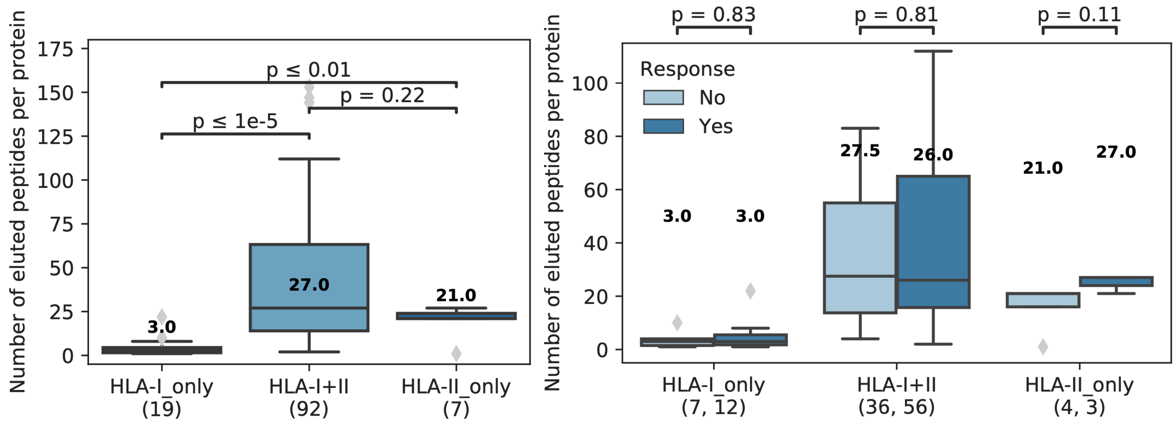

Appendix Figure S14. Analysis of neopeptide vaccine parent protein MHC elution patterns. A) Boxplots showing the number of eluted peptides in the HLA ligand atlas associated with the parent proteins of the 125 neopeptides evaluated by Sahin *et al*. The majority were from proteins from which peptides were found in both MHC-I and MHC-II eluted complexes. Parent proteins exclusive to MHC-I tended to have lower eluted peptide counts than parent proteins exclusive to MHC-II. B) Boxplots showing the number of eluted peptides as in panel A, but further divided according to whether the The number of MHC eluted peptides was not associated with post-vaccination response.

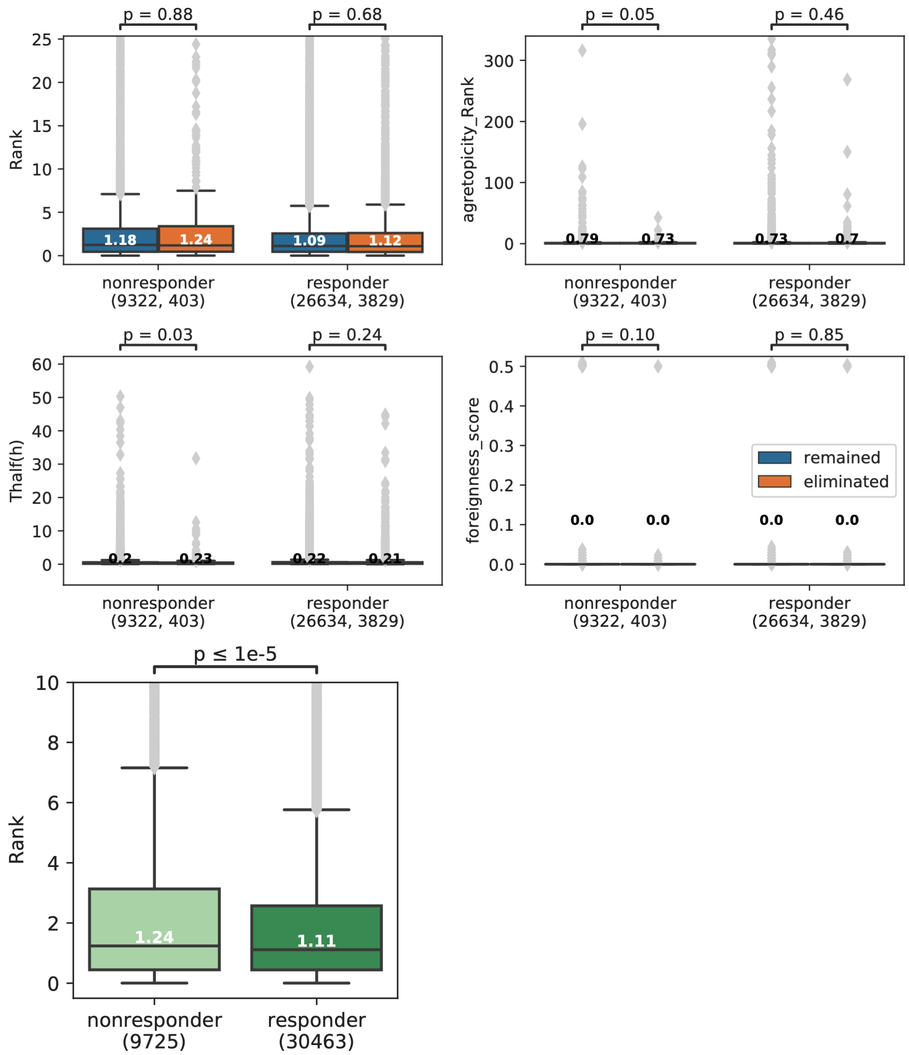

Appendix Figure S15. Comparison of neopeptide characteristics in the Riaz *et al*. dataset. (Top) Boxplots comparing affinity, agretopicity, stability, and foreignness between eliminated versus remaining neopeptides for both responders and nonresponders. (Bottom) Comparison of neopeptide affinity between responders and nonresponders.

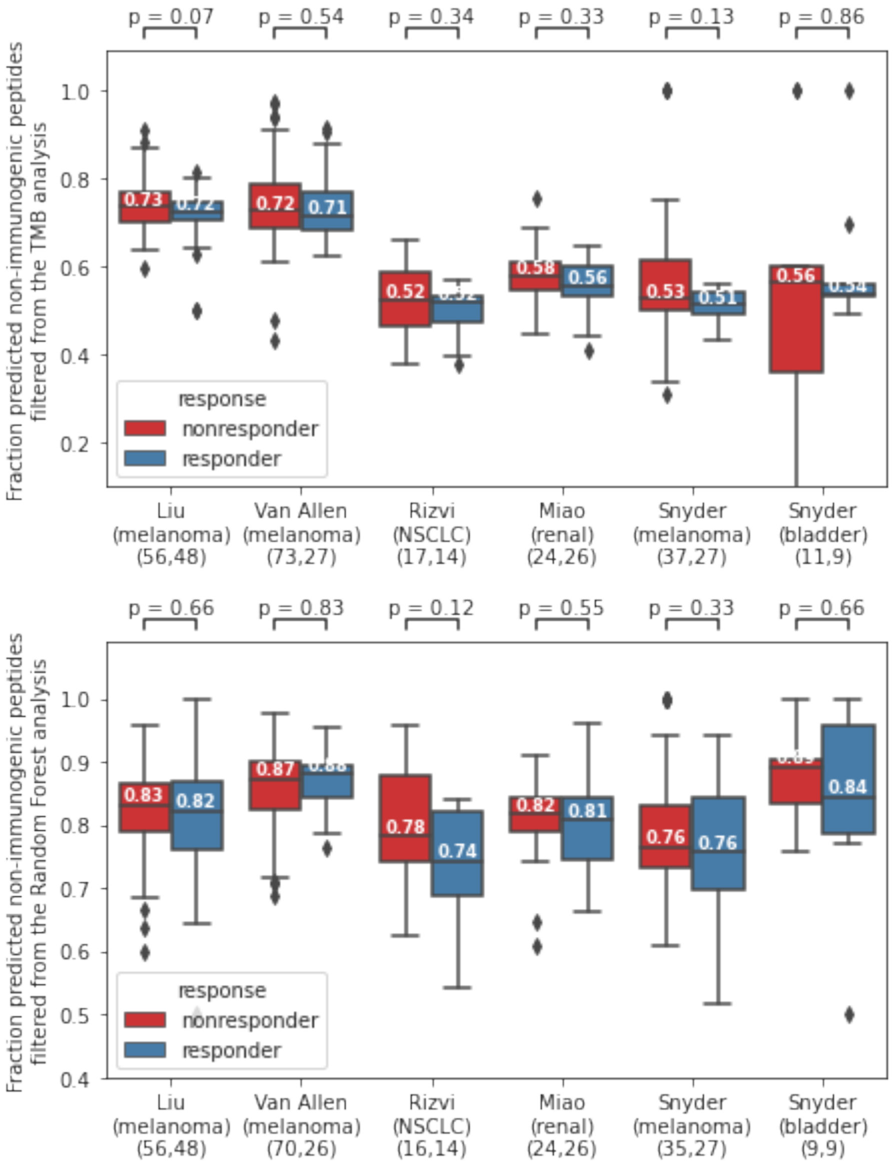

Appendix Figure S16. (Top) Boxplot comparing the distributions of the fraction of neoepitopes whose source proteins were not observed to come from previously observed immunogenic locations, and were filtered out between responders and non-responders. (Bottom) Boxplot comparing the distributions of the fraction of Random Forest predicted non-immunogenic peptides that were filtered out between responders and non-responders. The Mann-Whitney U test was used to calculate statistical significance between responders and non-responders. Multiple testing correction was not performed.

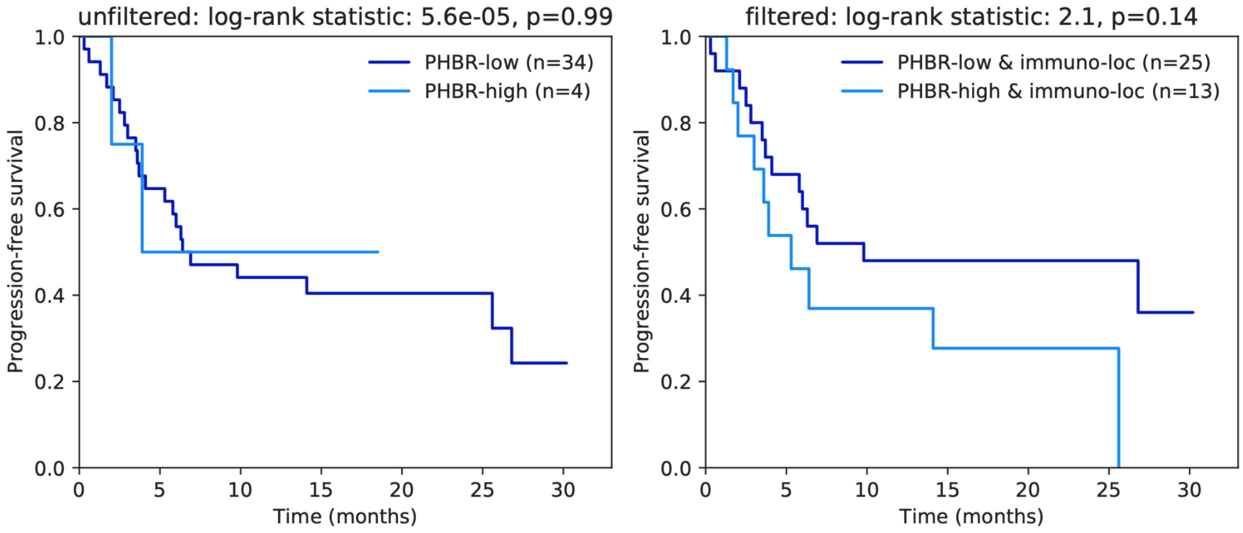

Appendix Figure S17. Kaplan Meier curves showing the effect of the best presented mutation on progression-free survival (C) using all genes in the panel and (D) using only the 40 genes of interest for high TMB patients.
