# Supplementary material for: Subcellular location of source proteins improves prediction of neoantigens for immunotherapy
Source: EMBO J. 2022 Oct 31;41(24):e111071. doi: 10.15252/embj.2022111071 (PMC9753441; doi:10.15252/embj.2022111071)
Supplement: Supplementary file 5 [file EMBJ-41-e111071-s001.pdf]

THE EMBO JOURNAL

Subcellular location of source proteins improves prediction of neoantigens for immunotherapy

Andrea Castro, Saghar Kaabinejadian, Hooman Yari, William Hildebrand, Maurizio Zanetti, and Hannah Carter
DOI: 10.15252/embj.2022111071

Corresponding author: Hannah Carter (hkcarter@health.ucsd.ed)

Review Timeline:

Submission Date:	16th Mar 22
Editorial Decision:	25th Apr 22
Revision Received:	3rd Aug 22
Editorial Decision:	16th Sep 22
Revision Received:	20th Sep 22
Accepted:	26th Sep 22

Editor: Kelly Anderson

Transaction Report:

(Note: With the exception of the correction of typographical or spelling errors that could be a source of ambiguity, letters and reports are not edited. Depending on transfer agreements, referee reports obtained elsewhere may or may not be included in this compilation. Referee reports are anonymous unless the Referee chooses to sign their reports.)

Dear Dr. Castro,

Thank you for submitting your manuscript for consideration by the EMBO Journal. It has now been seen by three referees whose comments are shown below.

Given the referees' recommendations, I would like to invite you to submit a revised version of the manuscript, addressing the comments of all three reviewers. I should add that it is EMBO Journal policy to allow only a single round of revision, and acceptance of your manuscript will therefore depend on the completeness of your responses in this revised version. It would be good to discuss your plan for revisions and I will be available to do so by zoom or email in the next couple weeks.

When preparing your letter of response to the referees' comments, please bear in mind that this will form part of the Review Process File, and will therefore be available online to the community. For more details on our Transparent Editorial Process, please visit our website: https://www.embopress.org/page/journal/14602075/authorguide#transparentprocess

We generally allow three months as standard revision time. As a matter of policy, competing manuscripts published during this period will not negatively impact on our assessment of the conceptual advance presented by your study. However, we request that you contact the editor as soon as possible upon publication of any related work, to discuss how to proceed. Should you foresee a problem in meeting this three-month deadline, please let us know in advance and we may be able to grant an extension. I have also attached a guide for revisions to this email.

Thank you for the opportunity to consider your work for publication. I look forward to your revision.

Yours sincerely,

Kelly M Anderson, PhD
Editor
The EMBO Journal
k.anderson@embojournal.org

Further information is available in our Guide For Authors: https://www.embopress.org/page/journal/14602075/authorguide

We realize that it is difficult to revise to a specific deadline. In the interest of protecting the conceptual advance provided by the work, we recommend a revision within 3 months (24th Jul 2022). Please discuss the revision progress ahead of this time with the editor if you require more time to complete the revisions. Use the link below to submit your revision:

Link Not Available

Referee #1:

The manuscript by Castro et al. presents compelling evidence for further consideration of the subcellular location of the protein encoded by MHC class I-restricted neoantigens in predicting immunogenicity and response to immunotherapy. This manuscript addresses a significant problem in that current computational methods are not able to accurately predict which neoantigens will elicit T cell responses. Neoantigen prioritization is critical for advancing immunotherapy of cancer. Protein subcellular location is known to impact the processing, availability for MHC loading and presentation of peptides; however, subcellular location has not been investigated for a contribution to predicting neoantigen immunogenicity. The question being explored is of interest to the fields of cancer and immunology.
Castro et al. demonstrate differences in the subcellular location for known immunogenic vs. non-immunogenic neoantigens and improvement in prediction of neoantigen immunogenicity with inclusion of the location characteristic. Patients responding to immune checkpoint inhibition have an increase in mutations in genes encoding proteins from immunogenic locations. Location may improve prediction of response to immunotherapy; there are small but statistically significant differences in some datasets. The major weaknesses are that it is not clear how much of the contribution of subcellular localization is due to expression (as multiple recent papers have demonstrated the importance of RNA expression of the variant in the tumor in predicting immunogenicity) and the lack of completeness and use of different approaches to investigate prediction of response to immune checkpoint inhibition in different datasets.
Major comments:
1. In Suppl Figure 1, how can you justify that the enrichment vs. depletion is due to subcellular location rather than expression given that the expression of the enriched peptides is always higher than the expression of the depleted proteins according to Suppl Figure 2? The statement that 62% of highly expressed genes were not significantly enriched does help explain this, but it

might be helpful to show whether combining expression and subcellular location improves prediction of which peptides are eluted. Are subcellular location and expression independent predictors of whether a peptide will be eluted? Note that in the event that subcellular location is not independent from RNA expression, subcellular location could be helpful in neoantigen prioritization, as neoantigen prioritization could be performed solely from WES and not require RNAseq.
2. What does the correlation of expression and subcellular location look like for the data shown in Figure 1 and Suppl Figure 7? This may also strengthen the authors claim that "location provides distinct information than expression".
3. It would be useful to include the feature importance plot for Suppl Figure 7 to see the relative importance of expression compared to location. If location shows increased importance compared to expression, this would strengthen the authors' claims.
4. As gene expression of the variant in the tumor is anticipated to be of greater importance than gene expression in normal tissues in predicting response to immune checkpoint inhibition (in the absence of neoantigen vaccine), it would be helpful to evaluate the effect of gene expression of the variant in the tumor vs subcellular location in Figure 3.
5. For Figure 3 in the Van Allen cohort, why were long survival patients separated out from responders? Does the significance of the comparison remain if the long survival group is added in with responders? It would seem counterintuitive if including the long survival patients took away the significance.
6. Why are the predictions done in different ways for Figure 3A and 3B? For completeness, can the data be presented for Liu with the model including location and for Van Allen only retaining "mutations in proteins from subcellular locations previously observed to source immunogenic peptides"?
Minor comments:
1. Suggest inclusion of additional citations for prior work investigating the importance of characteristics in neoantigen prioritization.
o Peptide:MHC stability: PMID: 35304420
o RNA expression of mutated gene in tumor: PMID: 35304420
o Hydrophobicity: PMID: 25831525, PMID: 31666118, PMID: 35304420, Wells, 2020
2. Suppl Figure 1B indicates that an "N" should be present to identify normal tissues, but no "N" is present.
3. The data being plotted in Suppl Figure 4 is unclear. Please expand the legend and add a y-axis label.
4. Suppl Figure 6: please add explanation for the lower plots.
5. For Suppl Figure 13, the text refers to persistent and eliminated neopeptides; please clarify how persistence or elimination of peptides is being determined.
6. The github repository is currently not accessible, though this may be intentional. Please change prior to publication.

Referee #2:

In this manuscript, Castro et al aim to study the impact of the subcellular location of a source protein on the immunogenic potential of its derivative peptides. They investigated peptides presented by the MHC and peptides documented to generate immune responses in various datasets. They find spatial biases in these peptides. They trained and evaluated machine learning models on datasets of neopeptides. The studies showed the trends and correlations between neoantigen source peptide location and immunogenicity and cancer immunotherapy response/outcomes. Authors conduct extensive analysis with various methods and datasets. The finding is interesting and novel. However, the authors have not convincingly proved a causal effect for location in immunogenicity/immunotherapy response. As authors note there are various limitations and possible bias. Some of the underlying potential biases that the authors note in their discussion could be driving the correlations that they discover.
Major
- We appreciate that the authors note that a limitation of this study are the possible sources of bias that could result in the correlation/trends presented. Much of work is based on the analysis of the available data that the authors have had no control.
-Figure 1A-1C. The hexplot visualization and the analysis that produced it is confusing. Does the difference in number of unique parent proteins between immunogenic and non-immunogenic in figure 1C correlate with the total number of parent proteins with a given ontology? Perhaps consider a normalized value to ensure that there is not spurious bias in 1C. If the individual comparisons between non-immunogenic and immunogenic protein locations were not significant, there should also be statistics to help the reader understand the significance of the differences in Fig 1C.
- Supplementary figure 4 is not clearly explained. What is the "total" on the vertical axis referring to? Not clear what authors are trying to show with this figure.
- It is unclear how to derive meaning from the analysis of the second independent dataset of 43 MHC-I neoepitopes when there are only 3 immunogenic peptides.
- Did the authors consider how mutations in proteins can affect the location of proteins? Some mutations drive protein expression to be nuclear, excluded from the nucleus, localized to proteasomes, etc. The authors noted this phenomenon in the discussion RE Melan-A, but is this information incorporated into the dataset?
- Did the authors consider other sources of protein subcellular location annotation?
- Re: "a cohort of 83 diverse tumors treated with immune checkpoint monotherapy that were profiled pre-treatment with the Foundation Medicine gene panel" Not sure how to interpretate these data because of the lack of clinical context. A such small cohort with diverse cancers which can have vastly different response/resistant to different immunotherapies (targeting CTLA-4, PD-1, PD-L1,among others) and different intrinsic cutoff high TMB

Minor
- Some statements are probably not very accurate; one example is the open statement of the Introduction "Accurate prediction of immunogenic neopeptides is crucial for the effective application of neoantigen-based cancer treatments such as neoantigen vaccines (probably, yes?), immune checkpoint blockade (probably, no?), and adoptive T cell therapy (yes/no?) ". Another example is the open statement of the Discussion "While immunotherapy has generated more durable responses than targeted therapies... "some targeted therapies are very durable...this sentence might not even essential for the discussion.
- What are the "previously observed immunogenic locations" at the top of page 8? Please clearly define it.
- Figure 4B, vertical axis label says "unfilted", should this be "unfiltered"
- The ordering of the plots in figure 4 is confusing. Considering putting both filtered analyses in the same row and the unfiltered analyses in the other row.
-page 3 " FInally, we evaluated source protein subcellular" should be finally....

Referee #3:

Source protein subcellular location is a novel feature to improve prediction of neoantigens for immunotherapy
Summary:
The study at hand investigates whether constraints on peptide accessibility to the MHC due to protein subcellular locations are associated with potential for peptide immunogenicity. The respective analyses have been performed on the level of MHC presentation as well as the immune response. In a first step, biases in the subcellular location could be shown for peptides eluted from MHC class I and II, following evaluations and modelling for predicting immunogenicity and immunotherapy response. The study starts with enrichment analyses for MHC presented peptides in differing components for MHC-I and MHC-II. Enrichment for MHC I and II eluted proteins such as the cytosol, nucleoplasm, and extracellular exosome components (MHC-I presented peptides) as well as extracellular proteins for MHC-II could therefore be confirmed (as shown before by Abelin et al, 2019; Bassani-Sternberg et al, 2015; Pearson et al. 2016). Interestingly, proteins from enriched locations tended to have longer predicted half-lives in this study- this might have an extensive impact on the final immune response. At this point of the study, an analysis on IEDB immunogenicity data could not reveal significantly enriched locations for immunogenic peptides aside a tendency of more eluted peptides on average.

The second part of the study investigates the possibility to use the parent protein location as predictor (one feature among others) for immunogenicity in several datasets. The impact of the location could be shown via a random forest model by adding this location information to an immune-response predictive feature set that comprised peptide-MHC binding affinity (Jurtz et al, 2017), peptide-MHC stability (Rasmussen et al, 2016), and foreignness (Luksza et al, 2017; Wells et al, 2020). The impact of location within the machine learning model could be shown within IEDB data but also by using the data from Wells et al. 2020 as test set. Apart from the location, there is the interesting insight here that even though the location correlated with expression, a benefit of including the location persisted even when the expression was also considered, showing that these two factors exhibit distinct information.
As third part of the analyses, associations between protein subcellular location and immunotherapy responses have been investigated. Starting with questioning the determinants for postvaccine response, an analysis showed that neoepitopes being able to induce a post-vaccination response were found to be enriched for the observed immunogenic locations. The majority of neoepitopes had parent proteins being eluted from both MHC-I and MHC-II, but the number of MHC eluted peptides from neopeptide parent proteins alone did not correlate with the response. While responders (melanoma) of immunotherapy had a better overall presentation of evaluated neopeptides, the investigation of location specific depletion revealed that eliminated neoepitopes did not have significantly better overall MHC allele specific presentations compared to retained neoepitopes. However, a tendency could be observed for enrichment of locations where immunogenic peptides have been previously observed.
Finally, the potential for parent protein location inclusion to improve ICB response stratification was analyzed as next step: Here, in one of two studies (Liu et al 2019) where mutation burden was associated with response, it could be shown that responders had a higher burden of proteins from locations where immunogenic responses have been previously observed. Those mutations from immunogenic locations as putative neoantigens still had a significantly higher burden of neoantigens relative to non-responders. Using those studies with immunogenicity information to train a model for classification of the neoepitopes in the two ICB cohorts into immunogenic or not could again shows that the prediction worked successfully, improved by location, supporting the potential of using the location to improve patient stratification pretreatment. Finally, including a score based on 40 panel genes from immunogenic locations, presentation was more significantly associated with outcome in high tumor mutation burden patients, concluding that in the setting of immunotherapy, the location is a determinant of the effective neoantigen burden.

Major concerns essential to be addressed to support the conclusions:
• The results about the capture of complex location patterns are not clearly described: In the first part, the gene ontology-based embedding has been used to do a UMAP dimensionality reduction. It is stated that this has been done for visualization and machine learning without more detailed explanation. There is only a reference to a figure, and the respective representation is not clearly explained. The outcome and use of this approach, as well as the conclusion are not entirely clear. A statement

regarding the insights should be given.
• The reduction of false positives is not evaluated in depth, and not shown in all datasets: The are statements about the differences of the datasets that are not followed up closely enough.
1. There are differences in the distribution of MHC affinity and stability of immunogenic peptides between the datasets that are extensive. An investigation about the level of affinity within the immunogenic IEDB data subset would be insightful (see also Figure 8a).
2. There is the observation that the origin of the peptides identified in Wells et al were infrequently observed in IEDB. The overlap and gap in numbers of proteins would be interesting, and how these facts impact the results. The analyses would also provide more insights with a more extensive description about distributions and overlaps, and analyses about the observed effects. The circumstance of reducing false positives within the Wells test as test set would be important to evaluate also in other datasets.
• The Investigation of the association between neoepitope parent protein and response (in the last part of the study) has been executed in only two studies- and while both studies have been performed in melanoma cohorts, an association could be found only in one study. Further analyses possibly including more cohorts should be done here to come to a clearer conclusion. After this insight about the different outcome, further analyses on the second cohort are described that include procedures such as the removal of predictions by affinity scores which was not part of the procedure before. The reason for this procedure is not clearly described and needs to be justified clearer.

Minor concerns that should be addressed:
• In the introduction, there is the following statement about other studies: "with non-standardized, and sometimes controversial, incorporation of other peptide characteristics such as peptide-MHC stability"- A more detailed explanation might be given why these characteristics are controversial and in which way these described features such as foreignness or mutation positions could be standardized according to the authors opinion. While mentioned in the introduction as problem, the authors do not address this in the following analyses.
• Similar, "the utility of these features for predicting immunogenicity varies across experiments and cohorts and ultimately"-It is stated that the presented features often vary if applied to other studies. As this is also the case in this study, this should be addressed in more detail (e.g., for the mentioned Wells vs IEDB distributions, or the reduction of false negatives).
• There is the interesting observation in the enrichment analyses that genes from enriched locations showed increased expression- but most of highly expressed genes were not significantly enriched in eluted pMHC. It could be insightful to investigate these circumstances in more detail.
• In the first part of the study, it is stated that for the Wells dataset, false positive predictions could be significantly reduced with location. It should be investigated in more detail what the differences are between the datasets regarding this outcome.
• The features differ between models (such as agretopicity is sometimes but not always included) without clear explanations about the differences and when the respective feature has been used.
• The thread is not always clear. Sometimes an impact of one feature to another is described, following a statement about the reverse direction being not necessarily the same, or the evaluation has been adapted. In these cases, an explanation would provide clarity. There are many side steps that could be explained better (reasoning of methods and sometimes details of results) which makes it hard to read and easily understand the text.

Referee #1:

The manuscript by Castro et al. presents compelling evidence for further consideration of the subcellular location of the protein encoded by MHC class I-restricted neoantigens in predicting immunogenicity and response to immunotherapy. This manuscript addresses a significant problem in that current computational methods are not able to accurately predict which neoantigens will elicit T cell responses. Neoantigen prioritization is critical for advancing immunotherapy of cancer. Protein subcellular location is known to impact the processing, availability for MHC loading and presentation of peptides; however, subcellular location has not been investigated for a contribution to predicting neoantigen immunogenicity. The question being explored is of interest to the fields of cancer and immunology.

Castro et al. demonstrate differences in the subcellular location for known immunogenic vs. non-immunogenic neoantigens and improvement in prediction of neoantigen immunogenicity with inclusion of the location characteristic. Patients responding to immune checkpoint inhibition have an increase in mutations in genes encoding proteins from immunogenic locations. Location may improve prediction of response to immunotherapy; there are small but statistically significant differences in some datasets. The major weaknesses are that it is not clear how much of the contribution of subcellular localization is due to expression (as multiple recent papers have demonstrated the importance of RNA expression of the variant in the tumor in predicting immunogenicity) and the lack of completeness and use of different approaches to investigate prediction of response to immune checkpoint inhibition in different datasets.

We thank the reviewer for the helpful comments and suggestions that resulted in further evidence supporting that subcellular location is distinct from expression as well as improving the clarity of the manuscript.

<u>Major comments</u>:

1. In Suppl Figure 1, how can you justify that the enrichment vs. depletion is due to subcellular location rather than expression given that the expression of the enriched peptides is always higher than the expression of the depleted proteins according to Suppl Figure 2? The statement that 62% of highly expressed genes were not significantly enriched does help explain this, but it might be helpful to show whether combining expression and subcellular location improves prediction of which peptides are eluted. Are subcellular location and expression independent predictors of whether a peptide will be eluted? Note that in the event that subcellular location is not independent from RNA expression, subcellular location could be helpful in neoantigen prioritization, as neoantigen prioritization could be performed solely from WES and not require RNAseq.

To examine whether location and expression were independent predictors of peptide elution, we used a dataset of eluted peptides from 721.221 B cells (n=3510) where we had matched gene expression (discussed in Supplementary Figure 1-4). Specifically, we sought to predict whether at least one peptide for a particular protein was found among the MHC-eluted peptides based on expression, location or the combination of both. The 3510 eluted peptides mapped to n=638

proteins for which there was corresponding gene expression data, leaving n=17,307 proteins for which we had gene expression but for which no peptides were detected. Thus we had n=638 positive and n=17,307 negative examples where a peptide was or was not eluted for a particular protein. We trained a random forest model using the same parameters as in Figure 1 to account for an imbalanced dataset (10-fold stratified cross-validation, shuffling, and balanced class weight). Location alone had significantly better AUROC (67% AUC) compared to expression alone (49% AUC) (DeLong's test p-value=1.34e-29). Location and expression together achieved an 81% AUROC and 15% AUPRC (**Figure R1**). This supports an independent contribution of location over expression. We have updated the manuscript to include this result as Figure 1C-D.

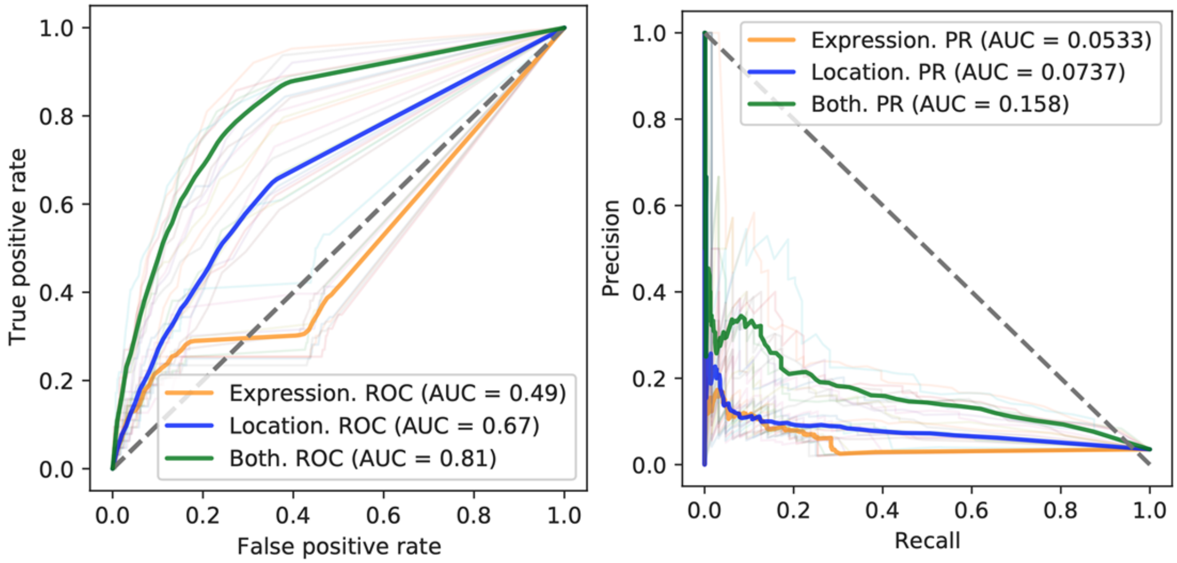

Figure R1. Overview of cross-validation results for the random forest model to predict peptide elution. (Left) Area under the receiver operating characteristic and (Right) area under the precision recall curve using expression as a feature (red), location (blue) or the combination (green). The faded lines indicate the respective area under the curve for each individual cross-validation partition while the darker lines indicate the average across partitions.

2. What does the correlation of expression and subcellular location look like for the data shown in Figure 1 and Suppl Figure 7? This may also strengthen the authors claim that "location provides distinct information than expression".

To quantify the correlation with expression and subcellular location both overall and for immunogenic versus non-immunogenic peptides, we analyzed expression with respect to location-derived clusters of parent proteins. We clustered the Poincare mapped location values using a K-means model specifically for the hyperbolic space (Popoff, 2018). We used the elbow method to select 7 clusters (**Figure R2A**) and plotted the relation to gene expression for each cluster (**Figure R2B**). We annotated each cluster with gene ontology (GO) cellular components and observed distinct differences in the cellular components represented by each cluster (**Table R1**). For example, cluster 5 was enriched for the nucleus and depleted for both extracellular

space and cell membrane, while cluster 6 was enriched for the extracellular space, cell membrane and endoplasmic reticulum.

We next evaluated gene expression levels within each cluster. We observed varying levels of gene expression across different locations, with many pairwise differences reaching statistical significance even after multiple testing correction (**Figure R2B**). Gene expression was higher in general in clusters 2 and 6 (enriched for cytosolic and extracellular proteins respectively) while levels were lowest in cluster 4 (no clear enrichment) and 5 (enriched for nucleus). Thus, we do see some correlation between expression and subcellular location that is consistent with previous reports (Abelin *et al*, 2017; Uhlén *et al*, 2015), though the distributions of gene expression show a considerable overlap across locations (**Figure R2B**).

We next looked at the association of gene expression with immunogenicity within locations and observed 3 clusters with significantly different gene expression for immunogenic versus non-immunogenic neopeptides. In two clusters (3,4) immunogenicity positively associated with expression while in one cluster (0) immunogenicity was inversely correlated with expression. The remaining four clusters did not have a significant association between immunogenicity and gene expression. Thus, while expression levels at different subcellular locations can differ, the distribution of expression levels are overlapping and the association of expression with immunogenicity is not uniform across locations. This further supports that location provides distinct information from expression.

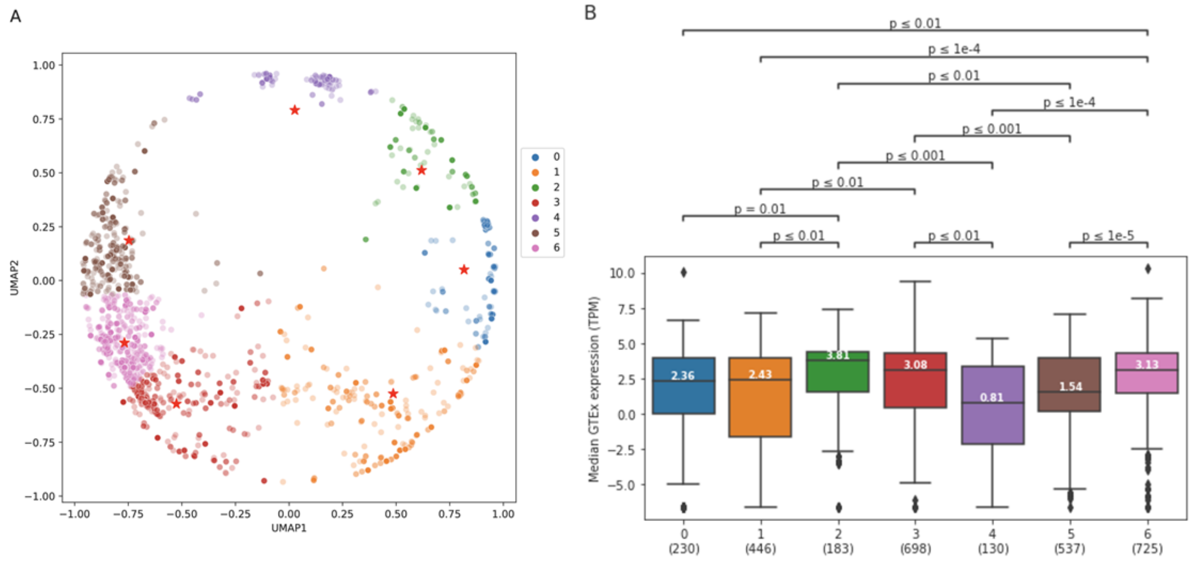

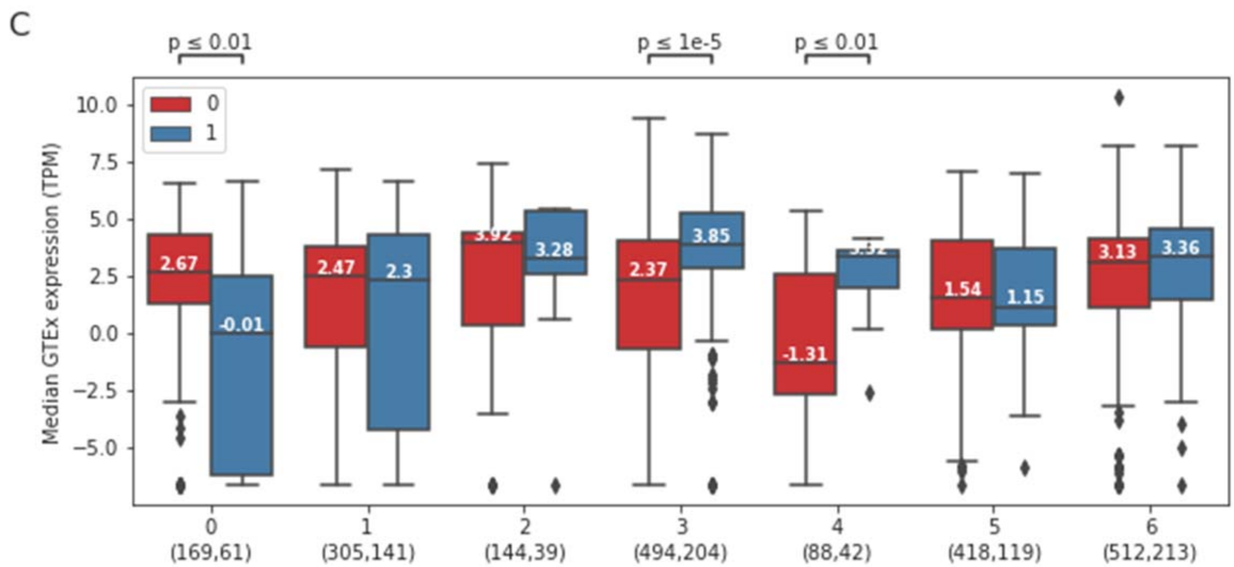

Figure R2. (A) Seven clusters after K-means clustering. Centroids are marked with red stars. (B) Boxplot correlating gene expression (TPM) with the seven location clusters. (C) Boxplot correlating gene expression with immunogenicity (blue) in the seven location clusters. All p-values are calculated with the Mann-Whitney U statistical test with Bonferroni multiple testing correction.

Table R1. Significant enrichment or depletion of the seven location clusters with general location terms used by DeepLoc.

**Cluster**	**Nucleus**	**Cytoplasm (Cytosol)**	**Extracellular**	**Mitochondrion**	**Cell membrane (Plasma membrane)**	**Endoplasmic reticulum**	**Golgi apparatus**	**Lysosome/Vacuole**
0								
1	depleted	enriched		enriched	enriched	enriched	enriched	enriched
2		enriched			enriched	enriched	enriched	
3					enriched			
4								
5	enriched		depleted		depleted			
6			enriched		enriched	enriched		

3. It would be useful to include the feature importance plot for Suppl Figure 7 to see the relative importance of expression compared to location. If location shows increased importance compared to expression, this would strengthen the authors' claims.

We thank the reviewer for this suggestion. We observe that location has greater feature importance than median gene expression (**Figure R3**), and we have updated Supplementary Figure 7 to include a panel with the feature importances.

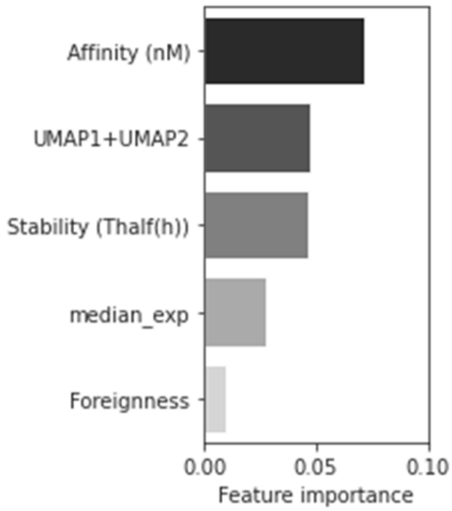

Figure R3. Barplot showing the Random Forest feature importance values corresponding to Supplementary Figure 7.

4. As gene expression of the variant in the tumor is anticipated to be of greater importance than gene expression in normal tissues in predicting response to immune checkpoint inhibition (in the absence of neoantigen vaccine), it would be helpful to evaluate the effect of gene expression of the variant in the tumor vs subcellular location in Figure 3.

We thank the reviewer for this suggestion.

As the majority of our training data did not have matched gene expression, we could not directly evaluate the model with tumor gene expression for the analyses in Fig 3. In an effort to compare gene expression vs subcellular location in the immunotherapy cohorts with RNA profiling available (Table R2), we quantified the variant allele specific expression using bamreadcount (https://github.com/genome/bam-readcount) and filtered out mutations/neopeptides that did not have at least 5 RNA reads supporting the variant allele (Table R2). We observe that after taking gene expression into account and filtering out non-expressed mutations, our results remain the same. We see that for the majority of cohorts (3 out of 4), filtering out mutations with parent proteins never before seen in immunogenic locations improves TMB-based stratification and filtering out neopeptides predicted to be immunogenic using the Random Forest model trained on experimentally validated immunogenic data improves stratification between responders and nonresponders in the majority of cohorts.

Table R2. Overview of validation immunotherapy datasets evaluated while accounting for RNA expression.

**Dataset**	**Number of**	**Number of**	**Effect sizes between responders**

	**patients with matched RNA sequencing**	**mutations/neoepitopes dropped after filtering for >5 supporting reads**	**and nonresponders after filtering** TMB: with loc | without loc RF: with loc | without | 500nM
Snyder 2014 NEJM	None	N/A	N/A
Rizvi 2015 Science	None	N/A	N/A
Snyder 2017 PLOS Med	26/29	22,387 neopeptides total, median 470 per patient	TMB: **0.34** | 0.31 Random Forest: **0.21** | 0.15 | 0.18
Miao 2018 Science	30/70	1,925 mutations, median 56 per patient	TMB: **0.26** | 0.19 Random Forest: **0.83** | **0.83** | 0.74
Van Allen 2015 Science	40/110	12,208 mutations total, median 185 per patient	TMB: 0.10 | **0.23** Random Forest: **0.93** | 0.92 | 0.87
Liu 2019 Nature Med	122/144	42,843 mutations total, median 139 per patient	TMB: **0.08** | 0.06 Random Forest: 0.48 | **0.52** | 0.35

* For cohorts with majority matched RNA data, we filtered mutations and calculated effect sizes for the entire cohort (Snyder 2017, Liu 2019). For the rest of the cohorts with limited matched RNA, we calculated effect sizes for only patients with RNA (Miao 2018, Van Allen 2015)

5. For Figure 3 in the Van Allen cohort, why were long survival patients separated out from responders? Does the significance of the comparison remain if the long survival group is added in with responders? It would seem counterintuitive if including the long survival patients took away the significance.

We separated out the long-term survival patients in the Van Allen cohort following the original manuscript's three groupings (e.g. minimal or no clinical benefit, long-term survival with no clinical benefit, and clinical benefit). The long-term survival patients were originally considered as a separate subgroup as they had early progression on ipilimumab but overall survival exceeded 2 years (Van Allen *et al*, 2015). We have updated the text after adding additional immunotherapy cohorts as suggested by Reviewer 3 (below) and added additional definitions in the Methods:

- We found that in four out of six evaluated immunotherapy cohorts including melanoma, non-small cell lung cancer (NSCLC), bladder, and renal cancer patients (Liu *et al*, 2019; Van Allen *et al*, 2015; Rizvi *et al*, 2015; Miao *et al*, 2018; Snyder *et al*, 2014, 2017), considering only mutations from immunogenic locations in the TMB calculation improved stratification of responders versus non-responders, as defined in their respective original studies (**Figure 3A, C**).

- (Methods) We utilized the original labels of responder/non-responder from each respective manuscript without extra grouping. For example, we kept the three separate groupings from the Van Allen cohort: “responders”, “long-term survival with no clinical benefit”, and “nonresponders”.

We added the long-term survival with no clinical benefit group to the clinical benefit group and still observe a significant relationship between response and predicted neoantigen burden, with the greatest stratification in the model that incorporates location features (effect size 0.185) (**Figure R4**).

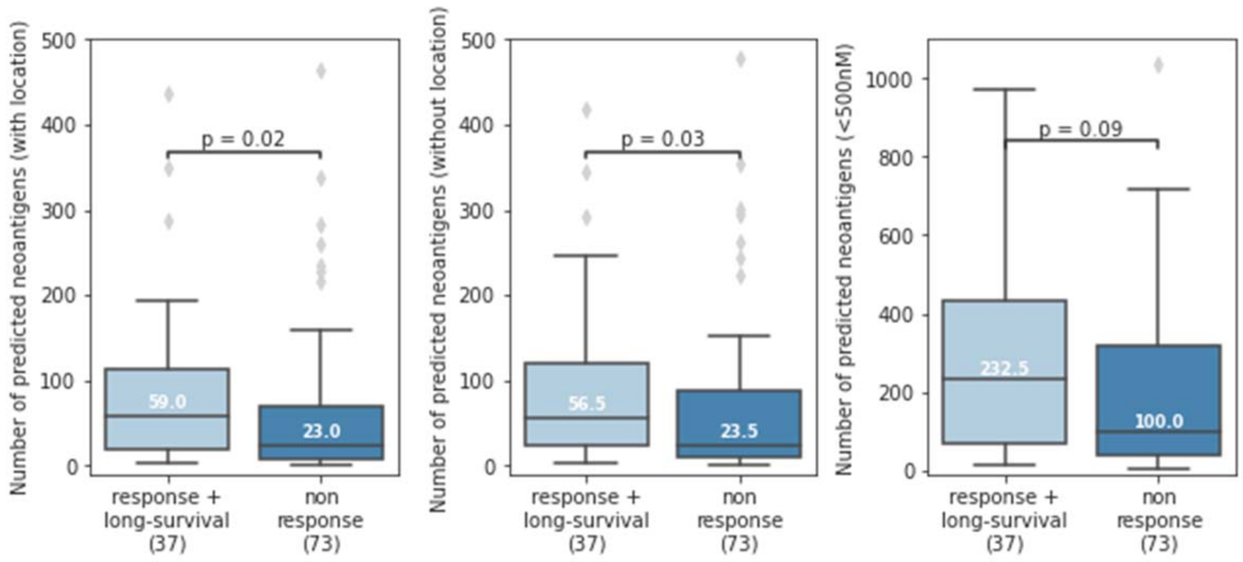

Figure R4. Boxplots comparing predicted neoantigen burden between responders + long-term survival patients versus nonresponders where neoantigen status is predicted using a model trained on 3 sources of immunogenic peptide and MHC affinity, stability and agretopicity, (A) with location, (B) without location, and (C) simply filtering out neopeptides with binding affinity >=500nM as described in the original manuscript. Effect sizes were calculated using Cliff’s D: (A) 0.185 (B) 0.167 and (C) 0.124.

6. Why are the predictions done in different ways for Figure 3A and 3B? For completeness, can the data be presented for Liu with the model including location and for Van Allen only retaining "mutations in proteins from subcellular locations previously observed to source immunogenic peptides"?

For consistency, we have updated Figure 3 to include boxplots for both location and predictive-model based filtering as well as overview figures describing effect sizes relative to a baseline of affinity based filtering (<500nM) for Liu and Van Allen cohorts as well as four additional immunotherapy cohorts we have now incorporated (listed in **Table R3**).

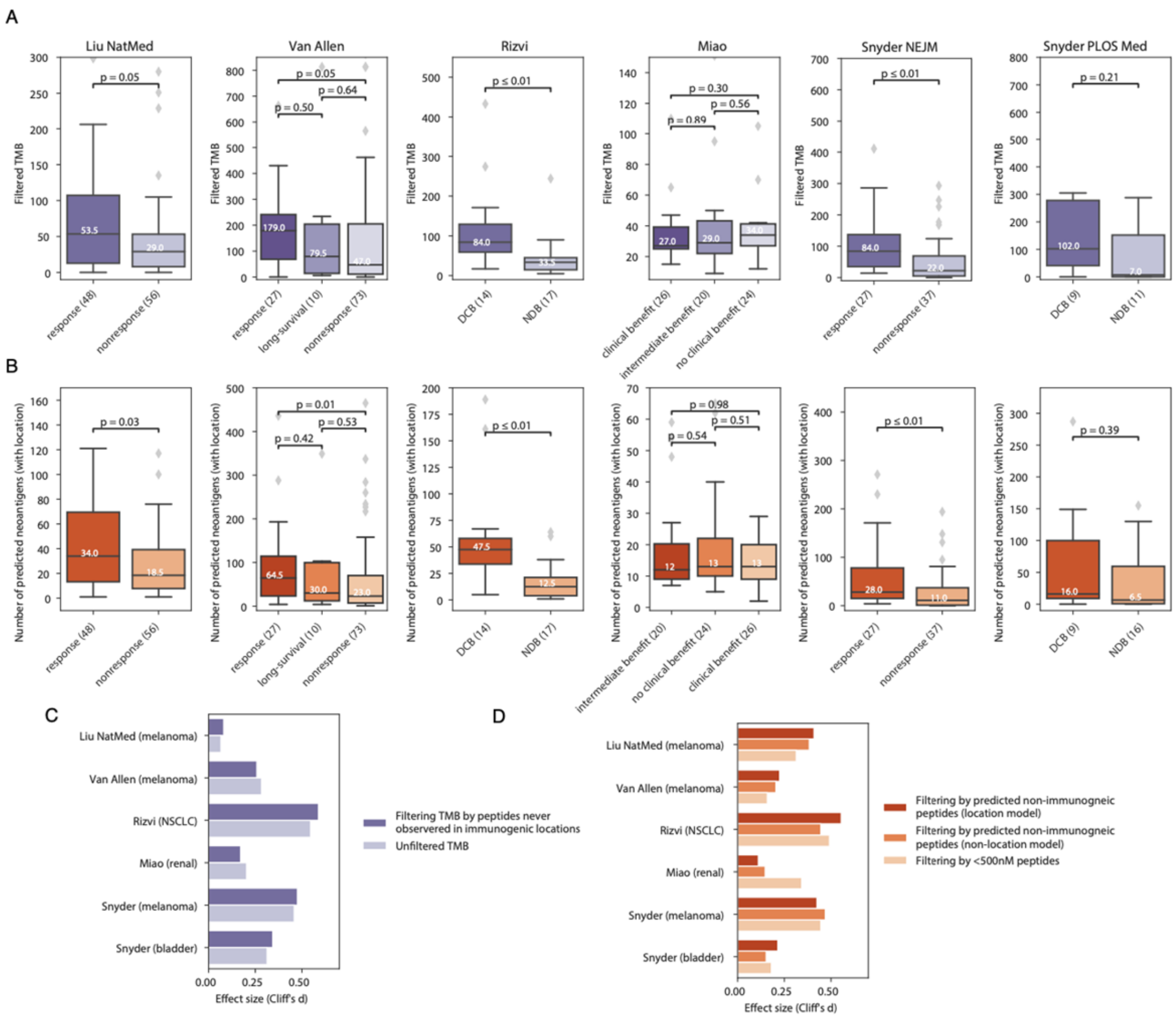

Updated Figure 3. ICB responders carry a higher burden of mutations in proteins from immunogenic locations. (A) Predicted neoantigen burden versus response category in immunotherapy cohorts when retaining only mutations in proteins from subcellular locations previously observed to source immunogenic peptides. (B) Predicted neoantigen burden versus response category in immunotherapy cohorts where neoantigen status is predicted using a model trained on 3 sources of immunogenic peptide and features including peptide MHC affinity, stability, and location. Barplots of effect sizes between responders and nonresponders (C) where TMB is filtered to include only mutations from subcellular locations previously observed to source immunogenic peptides and (D) where neoantigen status is predicted using a model trained on 3 sources of immunogenic peptide and MHC affinity and stability, with and without location.

<u>Minor comments</u>:

1. Suggest inclusion of additional citations for prior work investigating the importance of characteristics in neoantigen prioritization.

- Peptide:MHC stability: PMID: 35304420
- RNA expression of mutated gene in tumor: PMID: 35304420
- Hydrophobicity: PMID: 25831525, PMID: 31666118, PMID: 35304420, Wells, 2020

We thank the reviewer and have incorporated these suggested references in the introduction.

2. Suppl Figure 1B indicates that an "N" should be present to identify normal tissues, but no "N" is present.

We have updated the labels to now include an “N” to indicate normal tissues.

3. The data being plotted in Suppl Figure 4 is unclear. Please expand the legend and add a y-axis label.

We have updated the y-axis label to be “Total number of eluted peptides from both class I and II”

4. Suppl Figure 6: please add explanation for the lower plots.

We have added additional text for Supplementary Figure 6:

- “(Bottom) Boxplots comparing peptide-MHC affinity (nM), stability (Thalf (h)), foreignness scores, and median GTEx expression distributions between non-immunogenic (red) and immunogenic (gold) peptides. The Mann-Whitney U statistical test was performed.”

5. For Suppl Figure 13, the text refers to persistent and eliminated neopeptides; please clarify how persistence or elimination of peptides is being determined.

We thank the reviewer for this comment. We have modified the text (see also below) to clarify that “persistent” neopeptides means neopeptides that were present in the pre- and on-treatment samples for respective patients, and eliminated neopeptides were initially found in the pre-treatment samples, but not the on-treatment samples.

- “Since we found an association between parent protein subcellular location and post-vaccine response, we speculated that tumor clones present pre-treatment and eliminated during treatment would be more likely to harbor mutations in proteins from immunogenic locations. To further explore this possibility, we evaluated 73 melanoma patients with paired pre- and on-treatment samples to see if there were notable differences between eliminated (present pre-treatment and not on-treatment) and persistent (present both pre- and on-treatment) neopeptides (Riaz et al, 2017). We focused on responders (n=38, partial/complete response, or >6 months of stable disease) as these patients should have a relatively intact immune response compared to non-responders. While responders had better overall presentation of evaluated neopeptides, neopeptides eliminated on-treatment did not have significantly better

overall MHC allele-specific presentation compared to neopeptides retained pre- and on-treatment in both responders and nonresponders (**Supplementary Figure 13**), suggesting that neopeptide elimination is not driven solely by affinity or stability in this dataset."

6. The github repository is currently not accessible, though this may be intentional. Please change prior to publication.

The GitHub repo will be made publicly accessible prior to publication.

Referee #2:

In this manuscript, Castro et al aim to study the impact of the subcellular location of a source protein on the immunogenic potential of its derivative peptides. They investigated peptides presented by the MHC and peptides documented to generate immune responses in various datasets. They find spatial biases in these peptides. They trained and evaluated machine learning models on datasets of neopeptides. The studies showed the trends and correlations between neoantigen source peptide location and immunogenicity and cancer immunotherapy response/outcomes. Authors conduct extensive analysis with various methods and datasets. The finding is interesting and novel. However, the authors have not convincingly proved a causal effect for location in immunogenicity/immunotherapy response. As authors note there are various limitations and possible bias. Some of the underlying potential biases that the authors note in their discussion could be driving the correlations that they discover.

We thank the reviewer for many helpful comments that have strengthened the manuscript.

Major

- We appreciate that the authors note that a limitation of this study are the possible sources of bias that could result in the correlation/trends presented. Much of work is based on the analysis of the available data that the authors have had no control.

-Figure 1A-1C. The hexplot visualization and the analysis that produced it is confusing. Does the difference in number of unique parent proteins between immunogenic and non-immunogenic in figure 1C correlate with the total number of parent proteins with a given ontology? Perhaps consider a normalized value to ensure that there is not spurious bias in 1C. If the individual comparisons between non-immunogenic and immunogenic protein locations were not significant, there should also be statistics to help the reader understand the significance of the differences in Fig 1C.

We thank the reviewer for highlighting the potential issue with the interpretation of the hexplot visualization. We have moved the hexplots to the supplement and adopted a visualization that we hope is clearer. Specifically, we simply plotted each protein as an individual point. This avoids the indiscriminate groupings and counts produced by the hexplot that may have been influenced by the number of parent proteins studied at each location. We have updated the main text to remove the potentially misleading interpretation of the hexplots:

- We then used our 2D location embeddings to analyze the locations of unique source proteins across studies in the IEDB. We evaluated immunogenic and non-immunogenic neopeptide source proteins and observed many overlapping locations between the two groups (**Supplementary Figure 6A,B**). However, we still observed certain combinations of locations with more immunogenic peptides than not (**Supplementary Figure 6C**) and vice versa, though these may reflect selection biases involved in choosing the proteins and peptides evaluated for immunogenicity in the various IEDB studies and could evolve as available datasets grow.

We clustered IEDB source proteins according to their UMAP location features, selecting 7 clusters based on the elbow method, and annotated which proteins were COSMIC cancer genes (revised Figure 1). We performed cellular component enrichment analysis for each cluster, and found that different clusters were enriched or depleted for specific cellular components, demonstrating that the new representation preserves information about similar localization patterns of proteins.

To address whether the difference in the number of unique immunogenic parent proteins correlates with the total proteins, we evaluated whether the number of immunogenic parent proteins with identical GO cellular component terms is correlated with the total number of parent proteins with those terms. We found only a weak positive correlation (**Figure R5**).

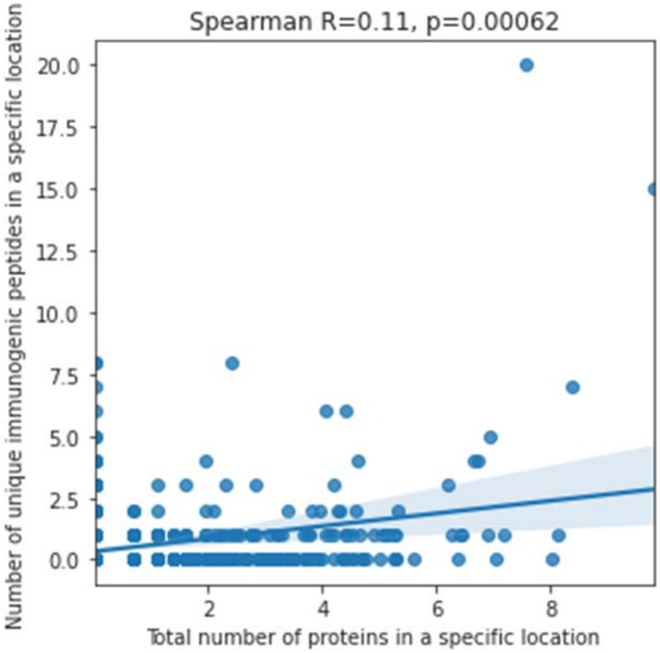

Figure R5. Scatterplot comparing the number of unique immunogenic peptides for a given location versus the total number of parent proteins in the same location. The Spearman R statistical test was used to calculate correlation and p-value.

We have now emphasized in the manuscript that the set of proteins/peptides with experimental findings in the IEDB represents a biased selection as these targets were studied for various reasons and not drawn at random from the proteome.

- Supplementary figure 4 is not clearly explained. What is the "total" on the vertical axis referring to? Not clear what authors are trying to show with this figure.

We have updated the y-axis label to “Total number of eluted peptides from both class I and II” to better match the description in the caption and the text.

- It is unclear how to derive meaning from the analysis of the second independent dataset of 43 MHC-I neoepitopes when there are only 3 immunogenic peptides.

Our intention with the analysis of 43 neoepitopes was to demonstrate the potential for generalization as experimentally validated neoantigen datasets grow. This limited dataset also helps illustrate the scarcity of truly immunogenic neoepitopes. We have updated the main text to emphasize this point:

- "Of these, only 3 (6.9%) were validated as immunogenic, further emphasizing the scarcity of true neoantigens. … Taken together, these findings suggest that immunogenicity prediction benefits from incorporating parent protein subcellular location and can be improved through aggregation of independent datasets across cancer types."

- Did the authors consider how mutations in proteins can affect the location of proteins? Some mutations drive protein expression to be nuclear, excluded from the nucleus, localized to proteasomes, etc. The authors noted this phenomenon in the discussion RE Melan-A, but is this information incorporated into the dataset?

We thank the reviewer for this comment. Though some localization differences caused by mutations have been described (Vieyra *et al*, 2003; Drikos *et al*, 2021), there is no comprehensive, experimentally-derived database of somatic mutations causing localization changes. To identify cases where peptide source proteins are predicted to have different localization as a result of a somatic mutation, we used DeepLoc (Almagro Armenteros *et al*, 2017) to predict subcellular localization on wildtype versus mutated peptide sequences. DeepLoc uses amino acid sequence to predict the following localizations: Nucleus, Cytoplasm, Extracellular, Mitochondrion, Cell membrane, Endoplasmic reticulum, Chloroplast, Golgi apparatus, Lysosome/Vacuole and Peroxisome.

We found that there were very few predicted differences in localization as a result of mutation - overall around 1% of mutations in each dataset (**Table R3, % different location column**) - and in most cases, for the wildtype sequence, the probability of the two locations was very similar (**Median/mean probability difference column**). Of the mutations predicted to cause differential localization, only one occured in a peptide that was annotated as immunogenic in the IEDB, and a minority were in peptides predicted to be immunogenic based on the Random Forest model in the immunotherapy datasets (**Number of immunogenic/predicted immunogenic peptides affected column**).

Table R3. DeepLoc analysis on the various immunotherapy and peptide datasets, detailing what percent of mutations affected the localization of the affected source protein, and how many localization changes affected immunogenic or predicted immunogenic peptides.

Dataset	% different location	Median/mean probability difference	Top location changes	Number of immunogenic (or predicted immunogenic*) peptides affected

IEDB	12/1587 (0.76%)	Median: -0.0045; Mean: -0.0037	Cytoplasm → Nucleus (6)<br>ER → Cell membrane (3)<br>Mitochondrion → ER (2)<br>ER → Peroxisome (1)	1/12 (8%)
Van Allen 2015 Science	155/18,592 (0.83%)	Median: 0.0005; Mean: 0.0057	Nucleus → Cytoplasm (27)<br>Cell membrane->ER (24)<br>Cytoplasm → Nucleus (24)<br>ER → Cell membrane (13)	19/155 (12%)*
Liu 2019 Nature Med	295/22,972 (1.2%)	Median: 0.0008; Mean: 0.001	Cytoplasm → Nucleus (53)<br>Nucleus → Cytoplasm (48)<br>Cell membrane → ER (43)<br>ER → Golgi apparatus (15)	64/295 (21%)*
Snyder 2014 NEJM	102/7620 (1.3%)	Median: -0.0001; Mean: 0.0058	Cytoplasm → Nucleus (16)<br>Nucleus → Cytoplasm (14)<br>Cell membrane → ER (11)<br>ER → Cell membrane (9)	32/102 (31%)*
Rizvi 2015 Science	24/2652 (0.9%)	Median: -0.0055, Mean: -0.0059	Nucleus → Cytoplasm (5)<br>Cytoplasm → Nucleus (3)<br>Cell membrane → ER (3)<br>Golgi apparatus → ER(3)	8/24 (33%)*
Snyder 2017 PLOS Med	93/7599 (1.2%)	Median: -0.0005, Mean: 0.007	Cytoplasm → Nucleus (16)<br>Nucleus → Cytoplasm (14)<br>Cell membrane → ER (11)<br>ER → Cell membrane (7)	1/93 (1%)*
Miao 2018 Science	41/3117 (1.3%)	Median: 0.0026, Mean: -0.0019	Cytoplasm → Nucleus (9)<br>Cytoplasm → Plastid (7)<br>Nucleus → Cytoplasm (7)<br>Cell membrane → ER (4)	4/41 (9.7%)*

- Did the authors consider other sources of protein subcellular location annotation?

We thank the reviewer for this question. We also considered location annotations from the Human Protein Atlas, UniProt subcellular location annotations, and DeepLoc. The Human Protein Atlas provides a spreadsheet of subcellular localizations that uses main locations from GO ontologies. UniProt subcellular location annotations are more free-text but correlate largely with the provided GO cellular component terms. We considered using DeepLoc to limit dependence on existing characterizations of protein subcellular localizations, *e.g.* in the scenario where novel mutations may alter localization. However, DeepLoc only provides 10 low resolution locations. Therefore, as there was a published method to create high dimensional embeddings for GO cellular component ontologies, we decided to take advantage of this method and dataset.

- Re: "a cohort of 83 diverse tumors treated with immune checkpoint monotherapy that were profiled pre-treatment with the Foundation Medicine gene panel" Not sure how to interpretate these data because of the lack of clinical context. A such small cohort with diverse cancers which can have vastly different response/resistant to different immunotherapies (targeting CTLA-4, PD-1, PD-L1,among others) and different intrinsic cutoff high TMB

We thank the reviewer for this comment. In our previous analysis of this cohort (Goodman *et al*, 2020), we found that TMB correlated with response even when we accounted for various confounding factors including sex, ethnicity, age, tumor type, TMB and monotherapy/combination therapy. The Goodman et al study also showed that incorporating information about MHC-I presentation of mutations in the panel genes could further stratify high TMB tumors according to response, even in the context of the covariates, suggesting that TMB can be further filtered to improve stratification given relevant information about putative neoantigens. In the present study, we were interested in assessing the potential clinical utility of incorporating source protein localization because the Foundation Medicine gene panel is widely used across a variety of tumor types in the clinic, and the ~300 genes used in the panel have been selected due to their driving roles in cancer. While our analysis suggests that a subset of these genes can be removed to reduce noise and improve stratification of responders versus nonresponders, we agree that this analysis would need to be studied across more cohorts. We have updated the main text to include more context:

- Finally, we analyzed a cohort of 83 diverse tumors treated with immune checkpoint monotherapy that were profiled pre-treatment with the Foundation Medicine gene panel (Goodman *et al*, 2020). In this cohort, we previously found that presence of at least one presentable driver mutation could further stratify responders and nonresponders in the context of covariates including sex, ethnicity, age, tumor type, TMB and therapy type.

<u>Minor</u>

- Some statements are probably not very accurate; one example is the open statement of the Introduction "Accurate prediction of immunogenic neopeptides is crucial for the effective application of neoantigen-based cancer treatments such as neoantigen vaccines (probably,

yes?), immune checkpoint blockade (probably, no?), and adoptive T cell therapy (yes/no?) ". Another example is the open statement of the Discussion "While immunotherapy has generated more durable responses than targeted therapies... "some targeted therapies are very durable...this sentence might not even essential for the discussion.

We thank the reviewer for these suggestions. We have modified the opening statement to emphasize that presence of immunogenic antigen is required for effective immunotherapy treatment and that characterizing immunogenic neopeptides could improve treatment application and identification of potential responders.

- “Presence of immunogenic antigen is necessary for neoantigen-based cancer treatments such as neoantigen vaccines, immune checkpoint blockade, and adoptive T cell therapy to be effective. These immunotherapies all depend on cell surface display of tumor-derived peptides by molecules of the major histocompatibility complex (MHC) mediating immune surveillance by T cells. As such, accurate characterization of the subset of immunogenic neoantigens should improve the design and application of immunotherapy and help identify potential responders.”

At the suggestion of the reviewer we have removed the comparison to targeted responses in the Discussion:

- “While immunotherapy has the ability to generate durable responses, the fraction of patients that respond remains relatively low.“

- What are the "previously observed immunogenic locations" at the top of page 8? Please clearly define it.

We have updated the text for clarity:

- “locations previously observed to contain immunogenic peptides from the Wells, Liu (OV) and IEDB neoantigen datasets”

- Figure 4B, vertical axis label says "unfilted", should this be "unfiltered"

We have corrected the typo.

- The ordering of the plots in figure 4 is confusing. Considering putting both filtered analyses in the same row and the unfiltered analyses in the other row.

We have updated the ordering to have unfiltered be the top row, and filtered be the bottom row.

-page 3 " FInally, we evaluated source protein subcellular" should be finally....

We have corrected the typo.

Referee #3:

Summary:
The study at hand investigates whether constraints on peptide accessibility to the MHC due to protein subcellular locations are associated with potential for peptide immunogenicity. The respective analyses have been performed on the level of MHC presentation as well as the immune response. In a first step, biases in the subcellular location could be shown for peptides eluted from MHC class I and II, following evaluations and modelling for predicting immunogenicity and immunotherapy response.

The study starts with enrichment analyses for MHC presented peptides in differing components for MHC-I and MHC-II. Enrichment for MHC I and II eluted proteins such as the cytosol, nucleoplasm, and extracellular exosome components (MHC-I presented peptides) as well as extracellular proteins for MHC-II could therefore be confirmed (as shown before by Abelin et al, 2019; Bassani-Sternberg et al, 2015; Pearson et al. 2016). Interestingly, proteins from enriched locations tended to have longer predicted half-lives in this study- this might have an extensive impact on the final immune response. At this point of the study, an analysis on IEDB immunogenicity data could not reveal significantly enriched locations for immunogenic peptides aside a tendency of more eluted peptides on average.

The second part of the study investigates the possibility to use the parent protein location as predictor (one feature among others) for immunogenicity in several datasets. The impact of the location could be shown via a random forest model by adding this location information to an immune-response predictive feature set that comprised peptide-MHC binding affinity (Jurtz et al, 2017), peptide-MHC stability (Rasmussen et al, 2016), and foreignness (Luksza et al, 2017; Wells et al, 2020). The impact of location within the machine learning model could be shown within IEDB data but also by using the data from Wells et al. 2020 as test set. Apart from the location, there is the interesting insight here that even though the location correlated with expression, a benefit of including the location persisted even when the expression was also considered, showing that these two factors exhibit distinct information.

As third part of the analyses, associations between protein subcellular location and immunotherapy responses have been investigated. Starting with questioning the determinants for postvaccine response, an analysis showed that neoepitopes being able to induce a post-vaccination response were found to be enriched for the observed immunogenic locations. The majority of neoepitopes had parent proteins being eluted from both MHC-I and MHC-II, but the number of MHC eluted peptides from neopeptide parent proteins alone did not correlate with the response. While responders (melanoma) of immunotherapy had a better overall presentation of evaluated neopeptides, the investigation of location specific depletion revealed that eliminated neoepitopes did not have significantly better overall MHC allele specific presentations compared to retained neoepitopes. However, a tendency could be observed for enrichment of locations where immunogenic peptides have been previously observed.
Finally, the potential for parent protein location inclusion to improve ICB response stratification was analyzed as next step: Here, in one of two studies (Liu et al 2019) where mutation burden

was associated with response, it could be shown that responders had a higher burden of proteins from locations where immunogenic responses have been previously observed. Those mutations from immunogenic locations as putative neoantigens still had a significantly higher burden of neoantigens relative to non-responders. Using those studies with immunogenicity information to train a model for classification of the neoepitopes in the two ICB cohorts into immunogenic or not could again shows that the prediction worked successfully, improved by location, supporting the potential of using the location to improve patient stratification pretreatment. Finally, including a score based on 40 panel genes from immunogenic locations, presentation was more significantly associated with outcome in high tumor mutation burden patients, concluding that in the setting of immunotherapy, the location is a determinant of the effective neoantigen burden.

We thank the reviewer for the thorough summary of our work and for many helpful comments that improve the clarity of the manuscript.

Major concerns essential to be addressed to support the conclusions:

• The results about the capture of complex location patterns are not clearly described: In the first part, the gene ontology-based embedding has been used to do a UMAP dimensionality reduction. It is stated that this has been done for visualization and machine learning without more detailed explanation. There is only a reference to a figure, and the respective representation is not clearly explained. The outcome and use of this approach, as well as the conclusion are not entirely clear. A statement regarding the insights should be given.

In the revised manuscript we sought to better justify the need for an embedding-based representation of complex protein localization and revised Figure 1 to better describe the utility of the approach. Specifically, we discuss that GO-based embeddings can be summed to account for multiple locations, and that reducing the 200D vectors into a more compact feature set is useful both for visualization and to provide a smaller feature set to avoid the curse of dimensionality for machine learning that nonetheless effectively captures location information (Figure 1A-B).

We have also added additional analyses that help demonstrate the utility in using the UMAP location features. Following suggestions from Reviewer 1, we used the location features to predict protein elution from MHC-I, and compared performance to that of gene expression (results included in the **revised Figure 1C-D**). We find that the location features are more informative than expression features in predicting elution, and that the combination of location and expression achieve 81% area under the curve.

A

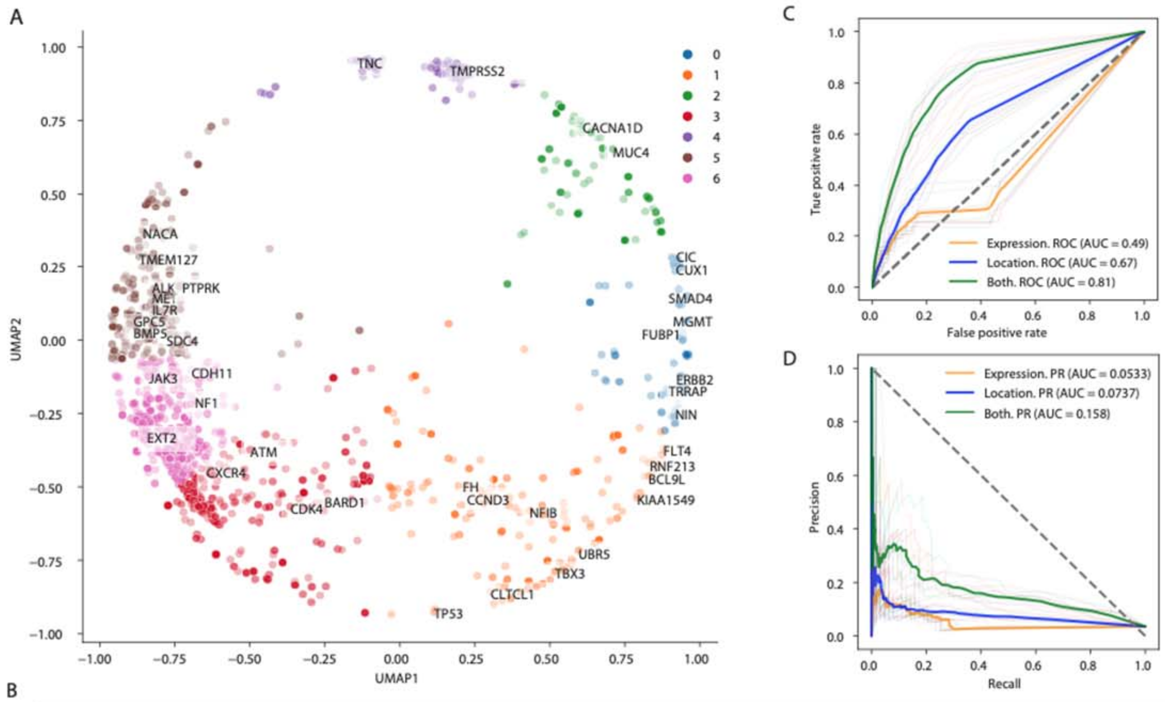

B

Cluster	Sig. enriched locations	Sig. depleted locations	COSMIC genes
0		Protein-containing complex, endomembrane system, organelle membrane, vesicle	CIC, CUX1, SMAD4, MGMT, FUBP1, ERBB2, TRRAP, NIN
1	Transcription regulator complex, transferase complex, nuclear protein-containing complex	Plasma membrane, integral component of membrane	FH, CCND3, NFIB, UBR5, TBX3, CLTCL1, TP53
2			CACNA1D, MUC4
3†#	Heterochromatin, sarcoplasmic reticulum, sarcolemma, proteasome regulatory particle, calcium channel complex		CXCR4, ATM, CDK4, BARD1
4	Integral component of membrane		TNC, TMPRSS2
5†	Receptor complex, integral component of plasma membrane	Membrane-bound organelle, intracellular organelle	NACA, TMEM127, ALK, MEG, PTPRK, IL7R, GPC5, BMP5, SDC4
6†	Lipid droplet, peroxisome, vesicle coat, dynein complex, integral component of ER membrane	Nucleus	JAK3, CDH11, NF1, EXT2

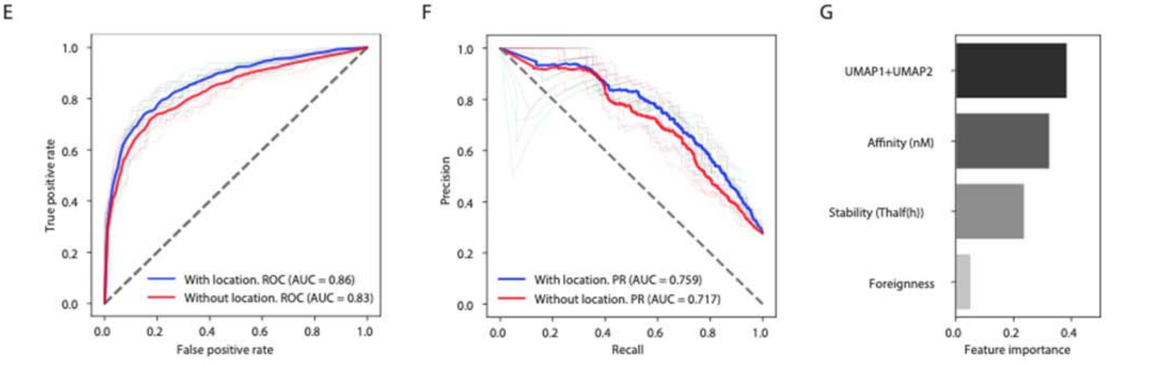

Updated Figure 1.

• The reduction of false positives is not evaluated in depth, and not shown in all datasets: The are statements about the differences of the datasets that are not followed up closely enough.

1. There are differences in the distribution of MHC affinity and stability of immunogenic peptides between the datasets that are extensive. An investigation about the level of affinity within the immunogenic IEDB data subset would be insightful (see also Figure 8a).

We performed additional investigation into the affinity differences between the immunogenic peptides in the Wells vs IEDB datasets. We find that while the most commonly evaluated allele in both the IEDB and Wells datasets is HLA-A02:01 (orange), the frequency of alleles used in assays to evaluate peptide immunogenicity differs between the groups (**Figure R6A-B**, shared alleles bolded and colored), and that the alleles within each dataset show variable affinity (**Figure R6C-D**) and stability (**Figure R6E-F**) across the peptides assayed for each. We have included this extended analysis as Supplementary Figure 10 and added additional commentary to emphasize the different motivations for the two datasets.

- Initial analysis revealed differences in the distribution of MHC affinity and stability of immunogenic peptides between the IEDB and Wells datasets (**Supplementary Figure 9**), which may be attributable to overall differences in MHC allele frequencies between the two datasets, and inherent differences in affinity and stability across MHC alleles themselves (Paul *et al*, 2013) (**Supplementary Figure 10**). We also found that the parent proteins of immunogenic peptides identified in Wells *et al*., originated from locations that were infrequently observed in the IEDB (**Supplementary Figure 10G**) likely reflecting the use of different criteria for selecting proteins/peptides in the Wells study versus studies in IEDB.

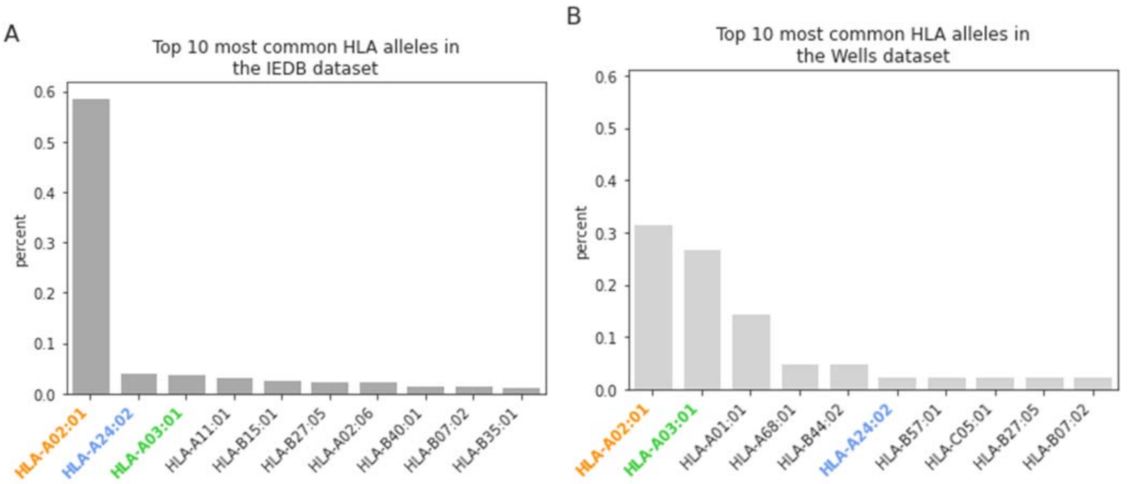

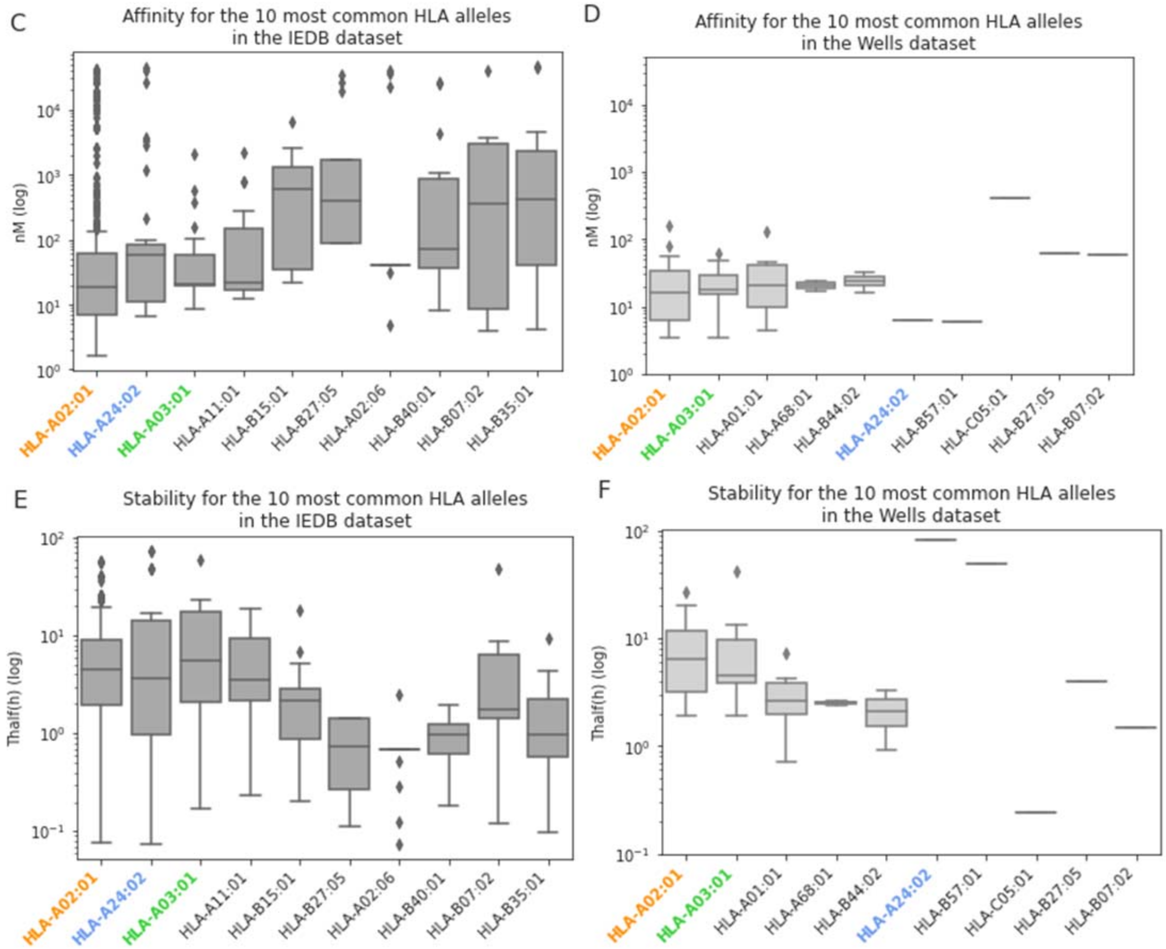

Figure R6. Overview of MHC characteristics for immunogenic peptides in the IEDB (left column) and Wells datasets (right column). Barplots denoting the frequencies of the 10 most common HLA alleles in the (A) IEDB and (B) Wells dataset that present immunogenic peptides. Boxplots showing the distribution of affinity (log nM) for these top 10 HLA alleles in the (C) IEDB and (D) Wells dataset. Boxplots showing the distribution of stability (log Thalf(h)) for these top 10 HLA alleles in the (E) IEDB and (D) Wells dataset.

2. There is the observation that the origin of the peptides identified in Wells et al were infrequently observed in IEDB. The overlap and gap in numbers of proteins would be interesting, and how these facts impact the results. The analyses would also provide more insights with a more extensive description about distributions and overlaps, and analyses about the observed effects. The circumstance of reducing false positives within the Wells test as test set would be important to evaluate also in other datasets.

We thank the reviewer for this suggestion. We evaluated how the overlap in source proteins compares to the overlap in source protein origins for the two datasets, finding overlaps of 37% and 14% respectively for protein origins and similarly 45% and 15% for the proteins themselves

(**Figure R7**). The low overlap in proteins evaluated likely reflects the different motivations for selecting proteins in the studies compiled by the IEDB and for the Wells study specifically where the goal was to predict immunogenic peptides from a set of 9 human tumors using peptide and MHC features. We analyzed the two datasets for cancer gene bias using the list of COSMIC Cancer Genes and found that the Wells neopeptides tended to come from cancer genes more frequently than the IEDB neopeptides, though the results were not quite statistically significant (Fisher's exact OR: 1.38, p=0.09).

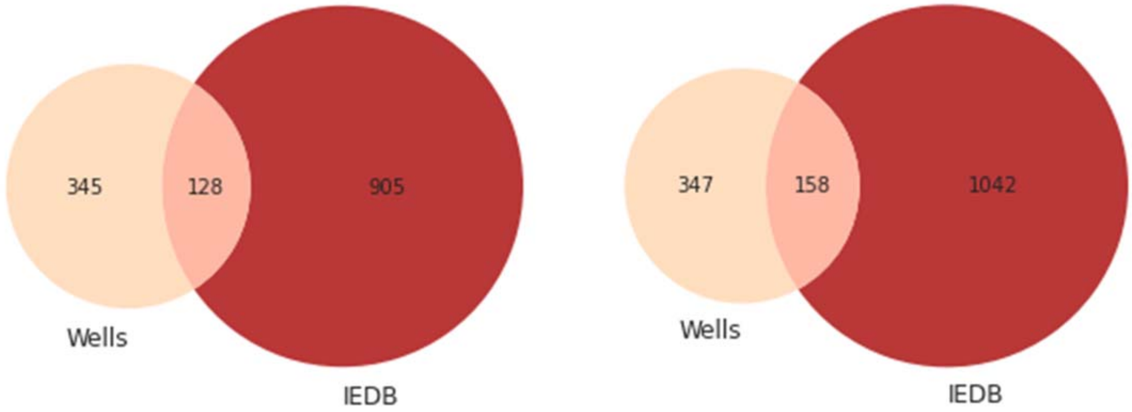

Figure R7. Venn diagrams showing the overlap between (Left) source protein location origins and (Right) proteins between the Wells and IEDB peptide datasets.

We sought to evaluate whether the reduction of false positives in the Wells and IEDB also translated to reduction of false positives in the immunotherapy treated cohorts. We speculated that responders should generally have a higher burden of actual immunogenic peptides than non-responders (though a lower burden of neoantigens may not be the sole reason for non-response). If so, then the increase in effect size observed for responders versus non-responders would translate to a larger reduction in the number of putative neoantigens in non-responders relative to responders (*i.e.* a reduction in false positives). Indeed, we found that both filtering and predictive modeling consistently removed a higher median fraction of mutations from nonresponders than responders, even though the differences between distributions were not statistically significant (**Figure R8**). We have added this figure as Supplementary Figure 16.

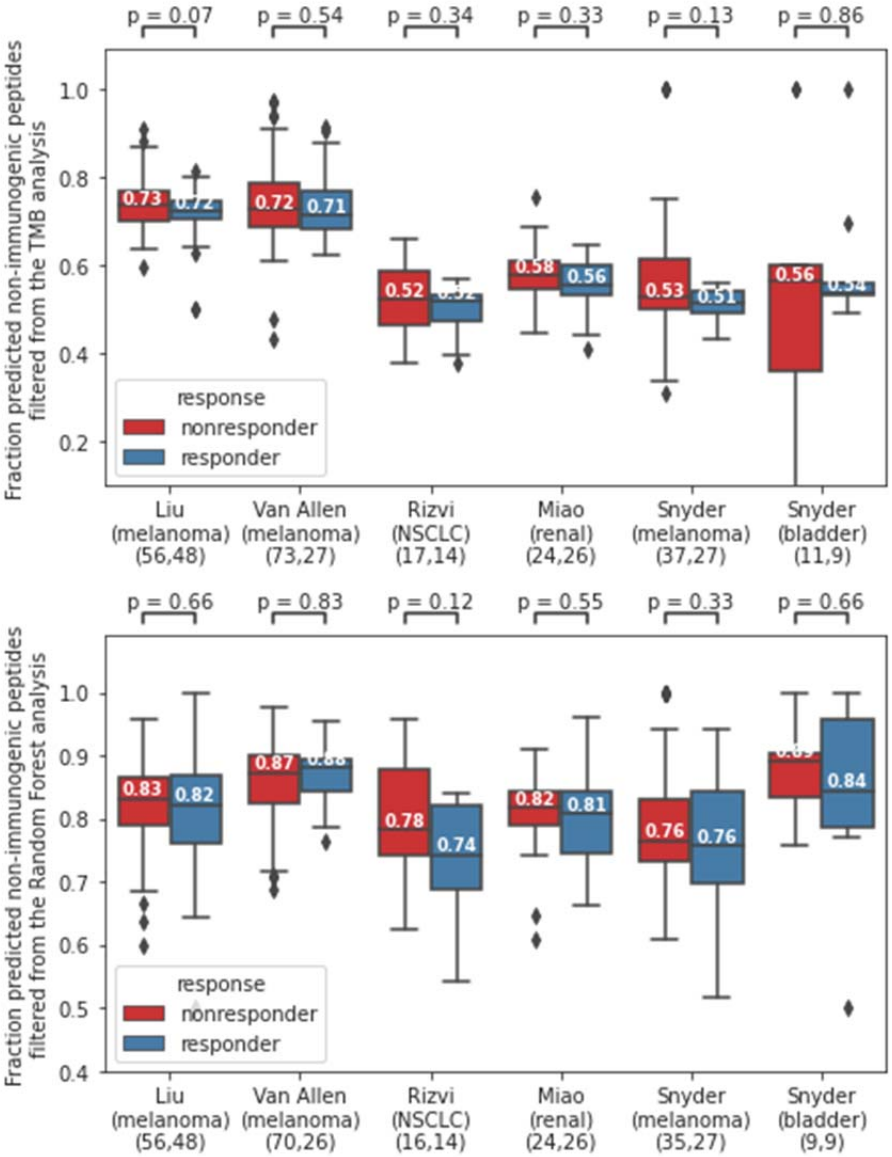

Figure R8. (Top) Boxplot comparing the distributions of the fraction of neoepitopes whose source proteins were not observed to come from previously observed immunogenic locations, and were filtered out between responders and non-responders. (Bottom) Boxplot comparing the distributions of the fraction of Random Forest predicted non-immunogenic peptides that were filtered out between responders and non-responders. The Mann-Whitney U test was used to calculate statistical significance between responders and non-responders. Multiple testing correction was not performed.

• The Investigation of the association between neoepitope parent protein and response (in the last part of the study) has been executed in only two studies- and while both studies have been performed in melanoma cohorts, an association could be found only in one study. Further analyses possibly including more cohorts should be done here to come to a clearer conclusion. After this insight about the different outcome, further analyses on the second cohort are

described that include procedures such as the removal of predictions by affinity scores which was not part of the procedure before. The reason for this procedure is not clearly described and needs to be justified clearer.

We thank the reviewer for this comment. Regarding the removal of predictions by affinity scores, we have updated the text to clarify that we ultimately evaluated two methods: (1) filtering raw nonsynonymous TMB to only consider mutations affecting proteins from previously observed immunogenic locations and (2) predicting immunogenic peptides using the Random Forest model trained on immunogenicity assay data from the IEDB, Wells, and Liu (OV). We compared these methods to baseline approaches, namely raw nonsynonymous TMB and TMB with poor affinity (>500nM) neopeptides removed.

We have now incorporated additional cohorts across different tumor types, including lung, bladder, and renal cancers (**Table R3**). We performed the two filtering analyses described above and found that these approaches tended to improve stratification across different cohorts and tumor types (**Figure R9**). To better understand why filtering based on location did not improve stratification in certain datasets, we evaluated the overlap between observed parent protein locations between the training versus immunotherapy validation sets. Interestingly, we observe more concordance in datasets with greater overall overlap in parent protein locations, particularly for the positive (immunogenic) class (**Table R4**, bolded rows). Altogether, our findings suggest that as the dataset of experimentally validated neoantigens increases, so should the power to effectively identify and filter out non-immunogenic neopeptides. We have included these expanded datasets in revised Figure 3 (also included below).

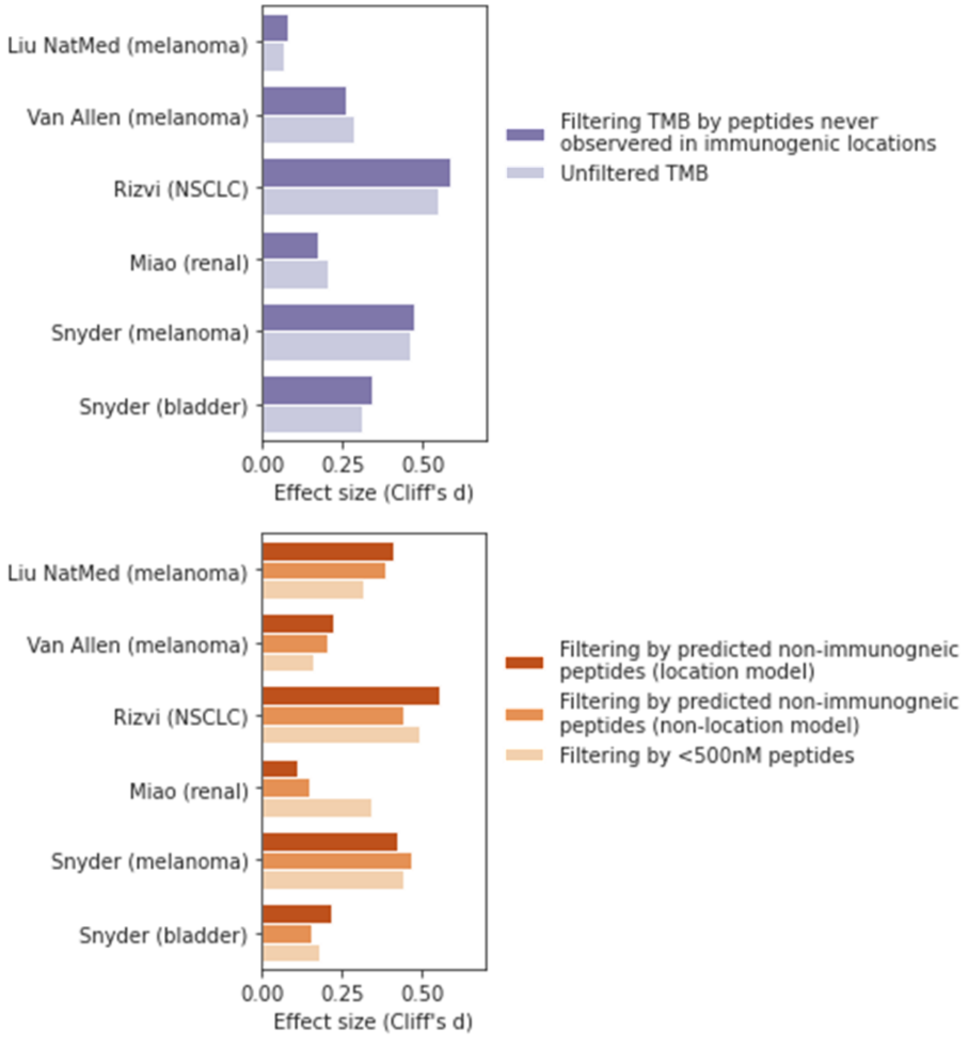

Figure R9. (Top) Barplot of effect sizes when filtering TMB based on locations previously observed to be immunogenic in the training dataset. (Bottom) Barplot of effect sizes when filtering TMB based on peptides predicted to be immunogenic.

Table R3. Additional immunotherapy cohorts considered or evaluated

**Dataset**	**Tumor type**	**Number of responders/nonresponders**
Snyder 2014 NEJM	Melanoma	27 responders, 37 non-responders
Rizvi 2015 Science	NSCLC	14 responders, 17 non-responders
Snyder 2017 PLOS Med	Bladder	9 responders, 16 non-responders
Miao 2018 Science	Renal	26 responders, 24 non-responders (20 intermediate)
Hugo 2016 Cell	Melanoma	7 complete responders, 14 partial responders, 17 progressive disease

		*Excluded due to reported high cross-sample contamination (Freeman *et al*, 2022)
Cristescu 2018 Science	Head/neck, melanoma. bladder, breast, colorectal, lung	*Labels not published in original manuscript - dataset not evaluated

Table R4. Summary of overlap between training data parent protein locations versus respective immunotherapy cohort neopeptides of interest

**Dataset**	**% locations overlapping with training data**	**% locations overlapping with positive label training data**
Snyder 2014 NEJM	18.2%	5.1%
**Rizvi 2015 Science**	**22.0%**	**7.8%**
**Snyder 2017 PLOS Med**	**22.8%**	**7.2%**
Miao 2018 Science	18.1%	5.8%
Van Allen 2015 Science	17.1%	4.4%
Liu 2019 Nature Med	15.15%	3.7%

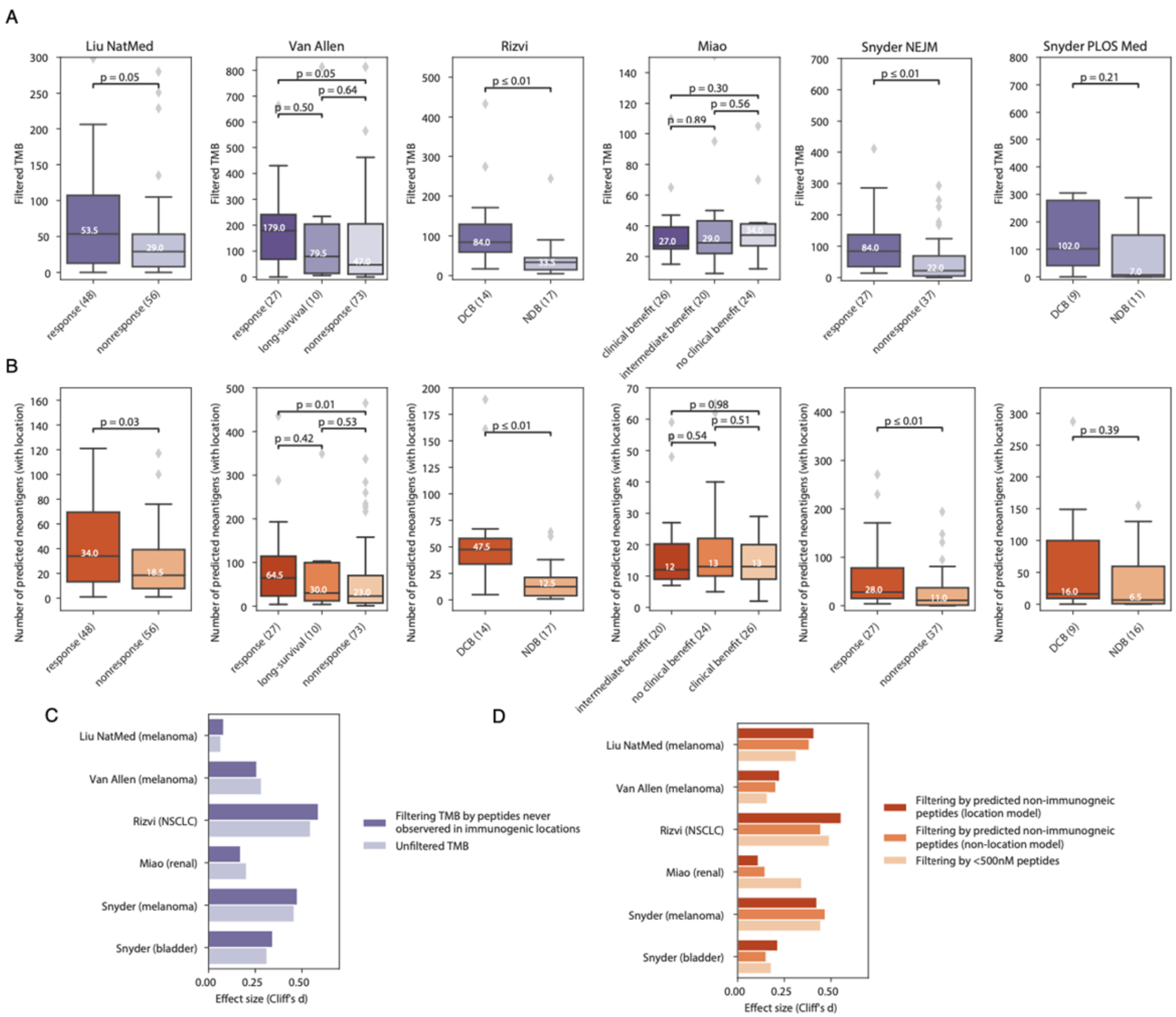

Updated Figure 3.

<u>Minor concerns that should be addressed:</u>

• In the introduction, there is the following statement about other studies: "with non-standardized, and sometimes controversial, incorporation of other peptide characteristics such as peptide-MHC stability"- A more detailed explanation might be given why these characteristics are controversial and in which way these described features such as foreignness or mutation positions could be standardized according to the authors opinion. While mentioned in the introduction as problem, the authors do not address this in the following analyses.

We have updated the text to include additional examples and references for the controversy statement (below). In our study, we focused on the features identified and validated by Wells *et al.* as the four independent features that enrich for immunogenicity in the largest study of immunogenicity available, namely peptide-MHC binding affinity, abundance in tumor cells, peptide-MHC stability, and foreignness.

- A number of features have been identified as informative for prioritizing immunogenic neopeptides (*i.e.* neopeptides that are both displayed and recognized by T cells as foreign) including peptide-MHC stability, agretopicity (Ghorani *et al*, 2018), foreignness (Łuksza *et al*, 2017), hydrophobicity (Chowell *et al*, 2015; Zhou *et al*, 2019; Borden *et al*, 2022; Wells *et al*, 2020), mutation position within the neopeptide (Schmidt *et al*, 2021), and neopeptide RNA abundance (Wells *et al*, 2020; Borden *et al*, 2022)(Wells *et al*, 2020) These features capture the potential for a peptide to be effectively presented by the MHC on the cell surface and address characteristics of the neopeptide itself. While initial studies tie these features to immunogenicity, their utility for predicting immunogenicity varies across experiments and cohorts; for example, hydrophobicity was initially associated with increasing T cell epitope prediction (Chowell *et al*, 2015), but later found to be unimportant for peptide filtering (Wells *et al*, 2020) Ultimately, current tools still yield many false positive neoantigen predictions (Yadav *et al*, 2014; Castro *et al*, 2021) (Yadav *et al*, 2014; Castro *et al*, 2021) capture other factors that contribute to T cell recognition of peptide-bound MHC.

• Similar, "the utility of these features for predicting immunogenicity varies across experiments and cohorts and ultimately"-It is stated that the presented features often vary if applied to other studies. As this is also the case in this study, this should be addressed in more detail (e.g., for the mentioned Wells vs IEDB distributions, or the reduction of false negatives).

We have now expanded our analysis to study a total of six immunotherapy treated cohorts and described differences in the Wells and IEDB datasets in more detail. These analyses demonstrate cross-cohort variability in the benefit of using location. While in the majority of cases we see benefit, there are nonetheless cohorts where using source protein location to filter TMB or predict immunogenic neoantigens does not seem to improve stratification. We also noted that for the latter, the representation of proteins and locations in the training set for the immunogenicity prediction model is a performance limiting factor. We have incorporated this into the discussion as follows:

- We used location to revise the effective neoantigen burden in tumors and better stratify potential for immunotherapy response, though the best performance was observed in tumor types similar to the training data, namely melanoma, lung, and bladder, as well as datasets with higher overlap in the locations of the source proteins studied.

• There is the interesting observation in the enrichment analyses that genes from enriched locations showed increased expression- but most of highly expressed genes were not significantly enriched in eluted pMHC. It could be insightful to investigate these circumstances in more detail.

As suggested by Reviewer 1, we have performed additional analyses to disentangle gene expression from location. We found that location is more informative than expression in predicting gene elution (0.67 ROC AUC vs 0.49 ROC AUC) and that the combination of location and expression together gives even better results (81% ROC AUC).

When revisiting highly expressed genes following this reviewer's suggestion, we identified an error in the estimate of the fraction of highly expressed genes that were not enriched in eluted peptides. We have corrected the error and report now that 22% of highly expressed genes are not eluted from the significantly enriched locations highlighted in Supplementary Figure 1B, which is more consistent with the findings in Pearson *et al.* (Pearson *et al*, 2016). We note that this does not affect the other expression analyses, namely the elution/immunogenicity predictions that incorporate expression and the correlation of expression with location. We analyzed this correct set of highly expressed genes that are not enriched in the eluted fractions and found that they tend to be associated with the caveola (on the surface of plasma membrane), the rough ER, and other membrane related terms.

• In the first part of the study, it is stated that for the Wells dataset, false positive predictions could be significantly reduced with location. It should be investigated in more detail what the differences are between the datasets regarding this outcome.

We have clarified in the text that we are referring to fewer false positives in Figure 2C (also included below for reference). i.e. Fewer non-immunogenic peptides (green points) are falling above the Youden index threshold for the model with location (vertical dashed line) than in the model without location (horizontal dashed line). The two models included peptide-MHC affinity, stability and foreignness, and only differed with respect to incorporation of location values or not.

- To address systematic differences in the feature sets, we trained a new model combining the IEDB with the Wells discovery set, and were able to achieve a higher recall on the test set (AUROC of 92%) while retaining the benefit for reducing false positives (69% AUPRC), shown by fewer non-immunogenic peptides (green points) falling above the Youden index threshold for the model with location (vertical dashed line) than in the model without location (horizontal dashed line) **(Figure 2A-C).**

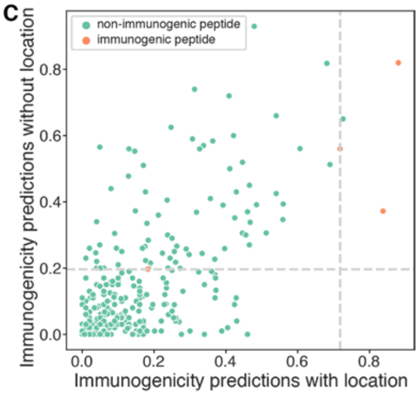

Figure 2C.

• The features differ between models (such as agretopicity is sometimes but not always included) without clear explanations about the differences and when the respective feature has been used.

We have updated the text to clarify that we focused on the four independent features that were associated with immunogenicity in the Wells analysis, specifically peptide-MHC binding affinity, peptide-MHC stability, foreignness, and tumor abundance (when matched expression data was available). We mistakenly listed agretopicity as a feature in the Figure 3 legend and have removed it.

- We performed 10-fold cross validation using a random forest classifier on the IEDB dataset (Methods) with and without adding location to a feature set that comprised peptide-MHC binding affinity (nM) (Jurtz *et al*, 2017), peptide-MHC stability (Rasmussen *et al*, 2016), and foreignness (Łuksza *et al*, 2017; Wells *et al*, 2020), all independent features that have been identified by Wells *et al* as enriching for immunogenicity.

• The thread is not always clear. Sometimes an impact of one feature to another is described, following a statement about the reverse direction being not necessarily the same, or the evaluation has been adapted. In these cases, an explanation would provide clarity. There are many side steps that could be explained better (reasoning of methods and sometimes details of results) which makes it hard to read and easily understand the text.

We have reorganized parts of the main text to improve flow and added more motivation for the key aspects to improve clarity, especially in our description of the use of embeddings to describe protein location. We also removed sentences that provided information that was not directly relevant to the results and discussion.

References

Abelin JG, Keskin DB, Sarkizova S, Hartigan CR, Zhang W, Sidney J, Stevens J, Lane W, Zhang GL, Eisenhaure TM, *et al* (2017) Mass Spectrometry Profiling of HLA-Associated Peptidomes in Mono-allelic Cells Enables More Accurate Epitope Prediction. *Immunity* 46: 315–326

Almagro Armenteros JJ, Sønderby CK, Sønderby SK, Nielsen H & Winther O (2017) DeepLoc: prediction of protein subcellular localization using deep learning. *Bioinformatics* 33: 3387–3395

Borden ES, Ghafoor S, Buetow KH, LaFleur BJ, Wilson MA & Hastings KT (2022) NeoScore Integrates Characteristics of the Neoantigen:MHC Class I Interaction and Expression to Accurately Prioritize Immunogenic Neoantigens. *J Immunol* 208: 1813–1827

Castro A, Zanetti M & Carter H (2021) Neoantigen Controversies.

Chowell D, Krishna S, Becker PD, Cocita C, Shu J, Tan X, Greenberg PD, Klavinskis LS, Blattman JN & Anderson KS (2015) TCR contact residue hydrophobicity is a hallmark of

immunogenic CD8+ T cell epitopes. *Proc Natl Acad Sci U S A* 112: E1754–62

Drikos I, Boutou E, Kastritis PL & Vorgias CE (2021) BRCA1-BRCT Mutations Alter the Subcellular Localization of BRCA1 In Vitro. *Anticancer Res* 41: 2953–2962

Freeman SS, Sade-Feldman M, Kim J, Stewart C, Gonye ALK, Ravi A, Arniella MB, Gushterova I, LaSalle TJ, Blaum EM, *et al* (2022) Combined tumor and immune signals from genomes or transcriptomes predict outcomes of checkpoint inhibition in melanoma. *Cell Rep Med* 3: 100500

Ghorani E, Rosenthal R, McGranahan N, Reading JL, Lynch M, Peggs KS, Swanton C & Quezada SA (2018) Differential binding affinity of mutated peptides for MHC class I is a predictor of survival in advanced lung cancer and melanoma. *Ann Oncol* 29: 271–279

Goodman AM, Castro A, Pyke RM, Okamura R, Kato S, Riviere P, Frampton G, Sokol E, Zhang X, Ball ED, *et al* (2020) MHC-I genotype and tumor mutational burden predict response to immunotherapy. *Genome Med* 12: 45

Jurtz V, Paul S, Andreatta M, Marcatili P, Peters B & Nielsen M (2017) NetMHCpan-4.0: Improved Peptide-MHC Class I Interaction Predictions Integrating Eluted Ligand and Peptide Binding Affinity Data. *J Immunol* 199: 3360–3368

Liu D, Schilling B, Liu D, Sucker A, Livingstone E, Jerby-Arnon L, Zimmer L, Gutzmer R, Satzger I, Loquai C, *et al* (2019) Integrative molecular and clinical modeling of clinical outcomes to PD1 blockade in patients with metastatic melanoma. *Nat Med* 25: 1916–1927

Łuksza M, Riaz N, Makarov V, Balachandran VP, Hellmann MD, Solovyov A, Rizvi NA, Merghoub T, Levine AJ, Chan TA, *et al* (2017) A neoantigen fitness model predicts tumour response to checkpoint blockade immunotherapy. *Nature* 551: 517–520

Miao D, Margolis CA, Gao W, Voss MH, Li W, Martini DJ, Norton C, Bossé D, Wankowicz SM, Cullen D, *et al* (2018) Genomic correlates of response to immune checkpoint therapies in clear cell renal cell carcinoma. *Science* 359: 801–806

Paul S, Weiskopf D, Angelo MA, Sidney J, Peters B & Sette A (2013) HLA class I alleles are associated with peptide-binding repertoires of different size, affinity, and immunogenicity. *J Immunol* 191: 5831–5839

Pearson H, Daouda T, Granados DP, Durette C, Bonneil E, Courcelles M, Rodenbrock A, Laverdure J-P, Côté C, Mader S, *et al* (2016) MHC class I-associated peptides derive from selective regions of the human genome. *J Clin Invest* 126: 4690–4701

Popoff A (2018) PoincareKMeans: K-Means algorithm in the Poincare Disk Model Github

Rasmussen M, Fenoy E, Harndahl M, Kristensen AB, Nielsen IK, Nielsen M & Buus S (2016) Pan-Specific Prediction of Peptide–MHC Class I Complex Stability, a Correlate of T Cell Immunogenicity. *The Journal of Immunology* 197: 1517–1524

Rizvi NA, Hellmann MD, Snyder A, Kvistborg P, Makarov V, Havel JJ, Lee W, Yuan J, Wong P, Ho TS, *et al* (2015) Cancer immunology. Mutational landscape determines sensitivity to PD-1 blockade in non-small cell lung cancer. *Science* 348: 124–128

Schmidt J, Smith AR, Magnin M, Racle J, Devlin JR, Bobisse S, Cesbron J, Bonnet V, Carmona

SJ, Huber F, *et al* (2021) Prediction of neo-epitope immunogenicity reveals TCR recognition determinants and provides insight into immunoediting. *Cell Rep Med* 2: 100194

Snyder A, Makarov V, Merghoub T, Yuan J, Zaretsky JM, Desrichard A, Walsh LA, Postow MA, Wong P, Ho TS, *et al* (2014) Genetic basis for clinical response to CTLA-4 blockade in melanoma. *N Engl J Med* 371: 2189–2199

Snyder A, Nathanson T, Funt SA, Ahuja A, Buros Novik J, Hellmann MD, Chang E, Aksoy BA, Al-Ahmadie H, Yusko E, *et al* (2017) Contribution of systemic and somatic factors to clinical response and resistance to PD-L1 blockade in urothelial cancer: An exploratory multi-omic analysis. *PLoS Med* 14: e1002309

Uhlén M, Fagerberg L, Hallström BM, Lindskog C, Oksvold P, Mardinoglu A, Sivertsson Å, Kampf C, Sjöstedt E, Asplund A, *et al* (2015) Proteomics. Tissue-based map of the human proteome. *Science* 347: 1260419

Van Allen EM, Miao D, Schilling B, Shukla SA, Blank C, Zimmer L, Sucker A, Hillen U, Foppen MHG, Goldinger SM, *et al* (2015) Genomic correlates of response to CTLA-4 blockade in metastatic melanoma. *Science* 350: 207–211

Vieyra D, Senger DL, Toyama T, Muzik H, Brasher PMA, Johnston RN, Riabowol K & Forsyth PA (2003) Altered subcellular localization and low frequency of mutations of ING1 in human brain tumors. *Clin Cancer Res* 9: 5952–5961

Wells DK, van Buuren MM, Dang KK, Hubbard-Lucey VM, Sheehan KCF, Campbell KM, Lamb A, Ward JP, Sidney J, Blazquez AB, *et al* (2020) Key Parameters of Tumor Epitope Immunogenicity Revealed Through a Consortium Approach Improve Neoantigen Prediction. *Cell* 183: 818–834.e13

Yadav M, Jhunjhunwala S, Phung QT, Lupardus P, Tanguay J, Bumbaca S, Franci C, Cheung TK, Fritsche J, Weinschenk T, *et al* (2014) Predicting immunogenic tumour mutations by combining mass spectrometry and exome sequencing. *Nature* 515: 572–576

Zhou C, Wei Z, Zhang Z, Zhang B, Zhu C, Chen K, Chuai G, Qu S, Xie L, Gao Y, *et al* (2019) pTuneos: prioritizing tumor neoantigens from next-generation sequencing data. *Genome Med* 11: 67

2nd Revision - Editorial Decision 16th Sep 2022

Dear Andrea,

Congratulations on a great revision! Overall, the referee we went back to was positive, however there remain a few editorial items to take care of in a revised version.

When you submit your revised version, please add these changes to your point-by-point response:

1. Please ensure that all file figures have Figure legends on the uploaded files, these are still missing

2. Thank you for updating the callouts to figures in the manuscript, but there is still one callout for Suppl. Data S1B missing, please correct.

3. Please view our new policy on conflict of interests on the EMBO author guide website and update the title of this section to: Disclosure and competing interests statement.

4. Please remove the Author Credit section from the manuscript.

Thank you for the opportunity to consider your work for publication. I look forward to your revision.

Yours sincerely,

Kelly

Kelly M Anderson, PhD
Editor
The EMBO Journal
k.anderson@embojournal.org

Referee #1:

The authors have addressed all of my prior concerns. This manuscript represents a significant advance in the field of neoantigen prediction by demonstrating the importance of source protein localization.

The authors performed the requested editorial changes.

2nd Revision - Editorial Decision 26th Sep 2022

Dear Andrea,

Congratulations on an excellent manuscript, I am pleased to inform you that your manuscript has been accepted for publication in the EMBO Journal. Thank you for your comprehensive response to the referee concerns. It has been a pleasure to work with you to get this to the acceptance stage.

I will begin the final checks on your manuscript before submitting to the publisher next week. Once at the publisher, it will take about 3 weeks for your manuscript to be published online. As a reminder, the entire review process, including referee concerns and your point-by-point response, will be available to readers.

I will be in touch throughout the final editorial process until publication. In the meantime, I hope you find time to celebrate!

Yours sincerely,

Kelly

Kelly M Anderson, PhD
Editor
The EMBO Journal
k.anderson@embojournal.org

EMBO Press Author Checklist

Corresponding Author Name: Hannah Carter	
Journal Submitted to: The EMBO Journal	
Manuscript Number: EMBOJ-2022-111071	

USEFUL LINKS FOR COMPLETING THIS FORM
The EMBO Journal - Author Guidelines
EMBO Reports - Author Guidelines
Molecular Systems Biology - Author Guidelines
EMBO Molecular Medicine - Author Guidelines

Reporting Checklist for Life Science Articles (updated January 2022)
This checklist is adapted from Materials Design Analysis Reporting (MDAR) Checklist for Authors. MDAR establishes a minimum set of requirements in transparent reporting in the life sciences (see Statement of Task: 10.31222/osf.io/9sm4x). Please follow the journal's guidelines in preparing your manuscript.
Please note that a copy of this checklist will be published alongside your article.

Abridged guidelines for figures
1. Data
The data shown in figures should satisfy the following conditions:
- the data were obtained and processed according to the field's best practice and are presented to reflect the results of the experiments in an accurate and unbiased manner.
- ideally, figure panels should include only measurements that are directly comparable to each other and obtained with the same assay.
- plots include clearly labeled error bars for independent experiments and sample sizes. Unless justified, error bars should not be shown for technical replicates.
- if n<5, the individual data points from each experiment should be plotted. Any statistical test employed should be justified.
- Source Data should be included to report the data underlying figures according to the guidelines set out in the authorship guidelines on Data Presentation.

2. Captions
Each figure caption should contain the following information, for each panel where they are relevant:
- a specification of the experimental system investigated (eg cell line, species name).
- the assay(s) and method(s) used to carry out the reported observations and measurements.
- an explicit mention of the biological and chemical entity(ies) that are being measured.
- an explicit mention of the biological and chemical entity(ies) that are altered/varied/perturbed in a controlled manner.
- the exact sample size (n) for each experimental group/condition, given as a number, not a range;
- a description of the sample collection allowing the reader to understand whether the samples represent technical or biological replicates (including how many animals, litters, cultures, etc.).
- a statement of how many times the experiment shown was independently replicated in the laboratory.
- definitions of statistical methods and measures:
  - common tests, such as t-test (please specify whether paired vs. unpaired), simple χ2 tests, Wilcoxon and Mann-Whitney tests, can be unambiguously identified by name only, but more complex techniques should be described in the methods section;
  - are tests one-sided or two-sided?
  - are there adjustments for multiple comparisons?
  - exact statistical test results, e.g., P values = x but not P values < x;
  - definition of 'center values' as median or average;
  - definition of error bars as s.d. or s.e.m.

Please complete ALL of the questions below.
Select "Not Applicable" only when the requested information is not relevant for your study.

Materials

Newly Created Materials	Information included in the manuscript?	In which section is the information available? (Reagents and Tools Table, Materials and Methods, Figures, Data Availability Section)
New materials and reagents need to be available; do any restrictions apply?	Not Applicable	N/A

Antibodies	Information included in the manuscript?	In which section is the information available? (Reagents and Tools Table, Materials and Methods, Figures, Data Availability Section)
For **antibodies** provide the following information:<br>- Commercial antibodies: RRID (if possible) or supplier name, catalogue number and or/clone number<br>- Non-commercial: RRID or citation	Not Applicable	N/A

DNA and RNA sequences	Information included in the manuscript?	In which section is the information available? (Reagents and Tools Table, Materials and Methods, Figures, Data Availability Section)
**Short novel DNA or RNA including primers, probes**: provide the sequences.	Not Applicable	N/A

Cell materials	Information included in the manuscript?	In which section is the information available? (Reagents and Tools Table, Materials and Methods, Figures, Data Availability Section)
**Cell lines:** Provide species information, strain. Provide accession number in repository **OR** supplier name, catalog number, clone number, and/**OR** RRID.	Yes	Methods
**Primary cultures:** Provide species, strain, sex of origin, genetic modification status.	Not Applicable	N/A
Report if the cell lines were recently **authenticated** (e.g., by STR profiling) and tested for mycoplasma contamination.	Not Applicable	N/A

Experimental animals	Information included in the manuscript?	In which section is the information available? (Reagents and Tools Table, Materials and Methods, Figures, Data Availability Section)
**Laboratory animals or Model organisms:** Provide species, strain, sex, age, genetic modification status. Provide accession number in repository **OR** supplier name, catalog number, clone number, **OR** RRID.	Not Applicable	N/A
**Animal observed in or captured from the field:** Provide species, sex, and age where possible.	Not Applicable	N/A
Please detail **housing and husbandry conditions**.	Not Applicable	N/A

Plants and microbes	Information included in the manuscript?	In which section is the information available? (Reagents and Tools Table, Materials and Methods, Figures, Data Availability Section)
**Plants:** provide species and strain, ecotype and cultivar where relevant, unique accession number if available, and source (including location for collected wild specimens).	Not Applicable	N/A
**Microbes:** provide species and strain, unique accession number if available, and source.	Not Applicable	N/A

Human research participants	Information included in the manuscript?	In which section is the information available? (Reagents and Tools Table, Materials and Methods, Figures, Data Availability Section)
If collected and within the bounds of privacy constraints report on age, sex and gender or ethnicity for all study participants.	Not Applicable	N/A

Core facilities	Information included in the manuscript?	In which section is the information available? (Reagents and Tools Table, Materials and Methods, Figures, Data Availability Section)
If your work benefited from core facilities, was their service mentioned in the acknowledgments section?	Not Applicable	N/A

Design

Study protocol	Information included in the manuscript?	In which section is the information available? (Reagents and Tools Table, Materials and Methods, Figures, Data Availability Section)
If study protocol has been **pre-registered, provide DOI in the manuscript**. For clinical trials, provide the trial registration number **OR** cite DOI.	Not Applicable	N/A
Report the **clinical trial registration number** (at ClinicalTrials.gov or equivalent), where applicable.	Not Applicable	N/A

Laboratory protocol	Information included in the manuscript?	In which section is the information available? (Reagents and Tools Table, Materials and Methods, Figures, Data Availability Section)
Provide DOI OR other citation details if **external detailed step-by-step protocols** are available.	Yes	Methods

Experimental study design and statistics	Information included in the manuscript?	In which section is the information available? (Reagents and Tools Table, Materials and Methods, Figures, Data Availability Section)
Include a statement about **sample size** estimate even if no statistical methods were used.	Yes	Main text, methods
Were any steps taken to minimize the effects of subjective bias when allocating animals/samples to treatment (e.g. **randomization procedure**)? If yes, have they been described?	Not Applicable	N/A
Include a statement about **blinding** even if no blinding was done.	Yes	Methods
Describe **inclusion/exclusion criteria** if samples or animals were excluded from the analysis. Were the criteria pre-established?<br><br>If sample or data points were omitted from analysis, report if this was due to attrition or intentional exclusion and provide justification.	Not Applicable	
For every figure, are **statistical tests** justified as appropriate? Do the data meet the assumptions of the tests (e.g., normal distribution)? Describe any methods used to assess it. Is there an estimate of variation within each group of data? Is the variance similar between the groups that are being statistically compared?	Yes	Methods

Sample definition and in-laboratory replication	Information included in the manuscript?	In which section is the information available? (Reagents and Tools Table, Materials and Methods, Figures, Data Availability Section)
In the figure legends: state number of times the experiment was **replicated** in laboratory.	Not Applicable	N/A
In the figure legends: define whether data describe **technical or biological replicates**.	Not Applicable	N/A

Ethics

Ethics	Information included in the manuscript?	In which section is the information available? (Reagents and Tools Table, Materials and Methods, Figures, Data Availability Section)
Studies involving **human participants**: State details of **authority granting ethics approval** (IRB or equivalent committee(s), provide reference number for approval.	Not Applicable	N/A
Studies involving **human participants**: Include a statement confirming that **informed consent** was obtained from all subjects and that the experiments conformed to the principles set out in the WMA Declaration of Helsinki and the Department of Health and Human Services Belmont Report.	Not Applicable	N/A
Studies involving **human participants:** For publication of **patient photos**, include a statement confirming that consent to publish was obtained.	Not Applicable	N/A
Studies involving experimental **animals**: State details of **authority granting ethics approva**l (IRB or equivalent committee(s), provide reference number for approval. Include a statement of compliance with ethical regulations.	Not Applicable	N/A
Studies involving **specimen and field samples:** State if relevant **permits** obtained, provide details of authority approving study; if none were required, explain why.	Not Applicable	N/A

Dual Use Research of Concern (DURC)	Information included in the manuscript?	In which section is the information available? (Reagents and Tools Table, Materials and Methods, Figures, Data Availability Section)
Could your study fall under dual use research restrictions? Please check biosecurity documents and list of **select agents and toxins** (CDC): https://www.selectagents.gov/sat/list.htm	Not Applicable	N/A
If you used a select agent, is the security level of the lab appropriate and reported in the manuscript?	Not Applicable	N/A
If a study is subject to dual use research of concern regulations, is the name of the **authority granting approval and reference number** for the regulatory approval provided in the manuscript?	Not Applicable	N/A

Reporting

The MDAR framework recommends adoption of discipline-specific guidelines, established and endorsed through community initiatives. Journals have their own policy about requiring specific guidelines and recommendations to complement MDAR.

Adherence to community standards	Information included in the manuscript?	In which section is the information available? (Reagents and Tools Table, Materials and Methods, Figures, Data Availability Section)
State if relevant guidelines or checklists (e.g., **ICMJE, MIBBI, ARRIVE, PRISMA**) have been followed or provided.	Not Applicable	N/A
For **tumor marker prognostic studies,** we recommend that you follow the **REMARK** reporting guidelines (see link list at top right). See author guidelines, under 'Reporting Guidelines'. Please confirm you have followed these guidelines.	Not Applicable	N/A
For **phase II and III randomized controlled trials**, please refer to the **CONSORT** flow diagram (see link list at top right) and submit the CONSORT checklist (see link list at top right) with your submission. See author guidelines, under 'Reporting Guidelines'. Please confirm you have submitted this list.	Not Applicable	N/A

Data Availability

Data availability	Information included in the manuscript?	In which section is the information available? (Reagents and Tools Table, Materials and Methods, Figures, Data Availability Section)
Have **primary datasets** been deposited according to the journal's guidelines (see 'Data Deposition' section) and the respective accession numbers provided in the Data Availability Section?	Not Applicable	All data is available in the supplementary
Were **human clinical and genomic datasets** deposited in a public access-controlled repository in accordance to ethical obligations to the patients and to the applicable consent agreement?	Not Applicable	
Are **computational models** that are central and integral to a study available without restrictions in a machine-readable form? Were the relevant accession numbers or links provided?	Yes	Upon publication, the github mentioned in the Methods will be available publically
If publicly available data were reused, provide the respective **data citations in the reference list**.	Yes	References
